# Multi-omics systems toxicology study of mouse lung assessing the effects of aerosols from two heat-not-burn tobacco products and cigarette smoke

**DOI:** 10.1016/j.csbj.2020.04.011

**Published:** 2020-04-25

**Authors:** Bjoern Titz, Justyna Szostak, Alain Sewer, Blaine Phillips, Catherine Nury, Thomas Schneider, Sophie Dijon, Oksana Lavrynenko, Ashraf Elamin, Emmanuel Guedj, Ee Tsin Wong, Stefan Lebrun, Grégory Vuillaume, Athanasios Kondylis, Sylvain Gubian, Stephane Cano, Patrice Leroy, Brian Keppler, Nikolai V. Ivanov, Patrick Vanscheeuwijck, Florian Martin, Manuel C. Peitsch, Julia Hoeng

**Affiliations:** a PMI R&D, Philip Morris Products S.A., Quai Jeanrenaud 5, CH-2000 Neuchâtel, Switzerland; b Philip Morris International Research Laboratories Pte. Ltd., Science Park II, Singapore; c Metabolon Inc., Research Triangle Park, NC, USA

**Keywords:** CHTP, Carbon Heated Tobacco Product, cMRTP, candidate modified risk tobacco product, COPD, chronic obstructive pulmonary disease, CS, cigarette smoke, LC, liquid chromatography, MOFA, Multi-Omics Factor Analysis, MS, mass spectrometry, PCSF, prize-collecting Steiner forest, ROS, reactive oxygen species, sGCCA, sparse generalized canonical correlation analysis, THS, Tobacco Heating System, Multi-omics, Systems toxicology, Cigarette smoking, Modified risk tobacco product (MRTP), Inhalation toxicology

## Abstract

Cigarette smoke (CS) causes adverse health effects and, for smoker who do not quit, modified risk tobacco products (MRTPs) can be an alternative to reduce the risk of developing smoking-related diseases. Standard toxicological endpoints can lack sensitivity, with systems toxicology approaches yielding broader insights into toxicological mechanisms. In a 6-month systems toxicology study on ApoE^−/−^ mice, we conducted an integrative multi-omics analysis to assess the effects of aerosols from the Carbon Heated Tobacco Product (CHTP) 1.2 and Tobacco Heating System (THS) 2.2—a potential and a candidate MRTP based on the heat-not-burn (HnB) principle—compared with CS at matched nicotine concentrations. Molecular exposure effects in the lungs were measured by mRNA/microRNA transcriptomics, proteomics, metabolomics, and lipidomics. Integrative data analysis included Multi-Omics Factor Analysis and multi-modality functional network interpretation. Across all five data modalities, CS exposure was associated with an increased inflammatory and oxidative stress response, and lipid/surfactant alterations. Upon HnB aerosol exposure these effects were much more limited or absent, with reversal of CS-induced effects upon cessation and switching to CHTP 1.2. Functional network analysis revealed CS-induced complex immunoregulatory interactions across the investigated molecular layers (e.g., itaconate, quinolinate, and miR-146) and highlighted the engagement of the heme–Hmox–bilirubin oxidative stress axis by CS. This work exemplifies how multi-omics approaches can be leveraged within systems toxicology studies and the generated multi-omics data set can facilitate the development of analysis methods and can yield further insights into the effects of toxicological exposures on the lung of mice.

## Highlights

• Multi-omics systems toxicology study, comprising five omics data modalities.
• Multi-Omics Factor Analysis and multi-modality functional network interpretation.
• Cigarettes smoke (CS) induced complex immunoregulatory interactions across molecular layers.
• Aerosols from two heat-not-burn tobacco products had less impact on lungs than CS.

## Graphical abstract

**Figure:**
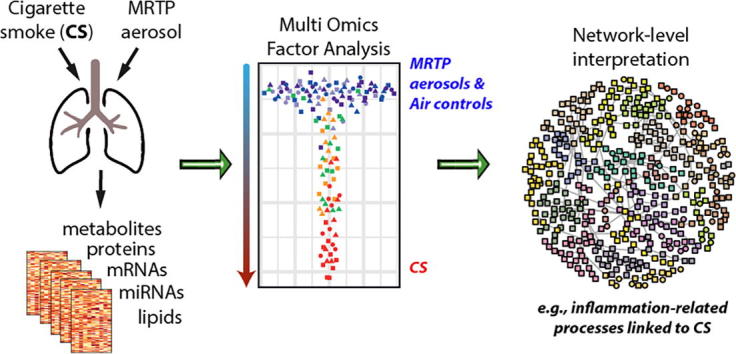

## 1 Introduction

Smoking is causally linked to several diseases, especially those of the respiratory and cardiovascular systems. Tobacco smoke exposure is a major risk factor for chronic obstructive pulmonary disease (COPD), a major and increasing global health problem [1]. While smoking cessation is the most effective measure for reducing the risk of smoking-related diseases [2], switching to less harmful products can be an alternative for smokers who would otherwise not quit.

The US Family Smoking Prevention and Tobacco Control Act defines a modified risk tobacco product (MRTP) as “any tobacco product that is sold or distributed for use to reduce harm or the risk of tobacco related disease associated with commercially marketed tobacco products” [3]. The Carbon Heating Tobacco System (CHTP) 1.2 and Tobacco Heating System (THS) 2.2 are potential and candidate MRTPs, respectively, developed by Philip Morris International based on the heat-not-burn principle [4], [5]. Tobacco is heated in a controlled fashion to release nicotine and volatiles that contribute to tobacco flavors, but combustion is prevented. To this end, THS 2.2 leverages electronic heating, while CHTP 1.2 has a carbon-heated tip from which heat is transferred to a tobacco plug. Thermal decomposition of organic tobacco compounds at elevated temperatures results in both pyrogenesis and pyrosynthesis of many harmful or potentially harmful constituents (HPHC). Therefore, preventing combustion produces an aerosol with lower number and levels of HPHC compared to cigarette smoke (CS) [5], [6].

Standard toxicological endpoints can lack sensitivity and only yield limited insights into toxicological mechanisms [7], [8]. By employing “omics” methods, systems toxicology complements these standard endpoints with comprehensive molecular analyses to increase sensitivity and coverage for detection of exposure effects [9], [10]. CS exposure broadly affects lung biology, with detrimental effects on lung lipids [11], [12], metabolites [13], [14], proteins [15], and transcriptional programs [16], [17]. Integrative analyses of multi-omics data can capture multilayer toxicological effects, as demonstrated in case of renal cisplatin toxicity [18], lung nanoparticle toxicity [19], cardiotoxicity of doxorubicin [20], and MRTP assessment [21]. On the basis of such studies, the need to further expand the use of such integrative multi-omics analyses has been emphasized in recent review articles [9], [22]. Here, we present results from a multi-omics analysis investigating the lung effects of aerosols from potential MRTPs, compared with CS, in a 6-month inhalation toxicity study on ApoE^−/−^ mice.

## 2 Material and methods

### 2.1 Overview of experimental design

Within the 6-month inhalation toxicity study on ApoE^−/−^ mice [23], [24], female ApoE^−/−^ mice were randomized into six groups: Sham, exposed to filtered air; 3R4F, exposed to CS from the 3R4F reference cigarette (600 µg total particulate matter [TPM]/L aerosol; target exposure concentration equivalent to 28 µg nicotine/L); CHTP 1.2, exposed to aerosol from CHTP 1.2 (nicotine levels matched to those of 3R4F CS equivalent to 28 µg nicotine/L); THS 2.2, exposed to aerosol from THS 2.2 (nicotine levels matched to those of 3R4F CS equivalent to 28 µg nicotine/L); Cessation, 3 months of exposure to 3R4F CS (600 µg TPM/L aerosol) followed by exposure to filtered air; and Switch to CHTP 1.2 aerosol, 3 months of exposure to 3R4F CS (600 µg TPM/L aerosol) followed by exposure to aerosol from CHTP 1.2 (nicotine levels matched to those of 3R4F CS equivalent to 28 µg nicotine/L). The maximum exposure duration was 6 months.

### 2.2 Animals

All procedures involving animals were performed in a facility accredited by the Association for Assessment and Accreditation of Laboratory Animal Care International and licensed by the Agri-Food and Veterinary Authority of Singapore, with approval from an Institutional Animal Care and Use Committee (IACUC protocol #15015) and in compliance with the National Advisory Committee for Laboratory Animal Research Guidelines on the Care and Use of Animals for Scientific Purposes (NACLAR, 2004).

More details on the animals are provided in the summary manuscript on this 6-month inhalation toxicity study [23] and on INTERVALS [24]. Briefly, female B6.129P2-Apoe^tm1Unc^ N11 ApoE^−/−^ mice bred under specific-pathogen-free conditions were obtained from Taconic Biosciences (Germantown, NY, USA). The mice were approximately 6 to 8 weeks old on arrival and 8 to 10 weeks old at the start of exposure. The mice were housed and exposed under specific hygienic conditions with filtered, conditioned, fresh air at 22 ± 2 °C and 55% ± 15% humidity. The light/dark cycle was 12 h/12 h. A maximum of eight mice were housed per cage.

### 2.3 Aerosol generation, characterization, and animal exposure

More details on the exposure and exposure characterization are provided in the summary manuscript on this 6-month inhalation toxicity study [23] and on INTERVALS [24]. Briefly, the mice were whole-body exposed to diluted mainstream CS from 3R4F cigarettes (target concentration 600 µg TPM/L, equivalent to 28 µg nicotine/L), CHTP 1.2 aerosol (nicotine-matched to 3R4F, 28 µg/L), THS 2.2 aerosol (nicotine-matched to 3R4F, 28 µg/L), or filtered air for 3 h per day, 5 days per week, for up to 6 months. Intermittent daily exposure to fresh, filtered air for 30 min after the first hour of smoke exposure and for 60 min after the second hour of exposure was provided to avoid build-up of excessive carboxyhemoglobin concentrations in the 3R4F group.

3R4F reference cigarettes [25] were purchased from the University of Kentucky. CHTP 1.2 uses a pressed carbon heat source to heat a tobacco plug in a specially designed stick to produce a nicotine-containing aerosol [5]. THS 2.2 consists of a single-use disposable stick containing a tobacco plug inserted into a holder—containing a battery, electronics for temperature control, a heating element, and a stick extractor [4], [6]—that heats the tobacco electrically in a controlled way to ensure that combustion temperatures are not reached. In both CHTP 1.2 and THS 2.2, the controlled heating of the tobacco generates an aerosol containing mainly water, glycerin, nicotine, and tobacco flavors. For detailed descriptions of CHTP 1.2 and THS 2.2, see [5], [6]. CHTP 1.2 and THS 2.2 sticks, as well as the holders, were provided by Philip Morris International (Neuchâtel, Switzerland).

Mainstream CS from 3R4F cigarettes was generated on 30-port rotary smoking machines, as described previously [26], while aerosols from CHTP 1.2 and THS 2.2 sticks were generated on modified 30-port rotary smoking machines equipped with the respective stick holders [5], [16]. Two modified smoking machines per chamber were required to achieve the target CHTP 1.2 and THS 2.2 aerosol concentrations. 3R4F cigarettes were smoked, and the aerosols from CHTP 1.2 and THS 2.2 sticks were generated in accordance with the Health Canada intense smoking protocol [27]. Several minor deviations from this protocol were necessary for technical reasons [26]. For example, butt length and static burning rate—typical smoking parameters—were measured only for the 3R4F machines or cigarettes, because they are only relevant for cigarettes. The puff count ranged from 10 to 11 puffs per cigarette (average 10.4 ± 0.3) for the 3R4F sticks. The CHTP 1.2 and THS 2.2 machines were always set for 12 puffs because of the device configuration. The 3R4F cigarettes were smoked to a butt length range of 34–36 mm (average 34.6 ± 0.4 mm), and the static burning rate was 467 s per 40 mm.

### 2.4 Exposure markers in urine

More details on the exposure characterization are provided in the summary manuscript on this 6-month inhalation toxicity study [23] and on INTERVALS [24]. Briefly, urine was collected for a 24-h period, including the three 1-h exposure periods. Biomarkers were determined by ABF GmbH (Munich, Germany) (N = 10–12); for details, see [16], [26].

### 2.5 Tissue preparation for omics analyses

Molecular analysis (transcriptomics, proteomics, miRNAs, genomics, and metabolomics) were performed after 3, 4, and 6 months of exposure. Tissues were collected 16–24 h after exposure (separate samples from the same tissues/organs for transcriptomics, proteomics, lipidomics, and genomics analyses) from eight to ten mice per group and processed as described previously [16]. For transcriptomics, proteomics, and lipidomics analyses, the left lung lobe was frozen on dry ice and stored at −80 °C. The lung lobe was subsequently cryosectioned, and slices were collected in alternating order for the different omics analyses.

### 2.6 Transcriptomics

Total RNA was isolated from tissues by using the miRNeasy Mini Kit (Qiagen, Hilden, Germany) and quality-checked by using an Agilent 2100 Bioanalyzer (Agilent Technologies, Santa Clara, CA, USA) (N = 9) [23]. Samples were processed and analyzed in randomized order. Total RNA (100 ng) was reverse-transcribed, amplified, purified, and hybridized on MG430 2.0 GeneChips (Affymetrix, Santa Clara, CA, USA) and evaluated by using standard procedures (for details, see Phillips et al. [16] and the protocol on INTERVALS [28]). miRNA was analyzed by using microarrays and a previously described method [14] (see protocol on INTERVALS [29]).

For statistical analysis, a linear model was fitted for each exposure condition and the respective Sham group, *p* values were calculated from moderated *t*-statistics with the empirical Bayes approach [30], and genes with a Benjamini–Hochberg FDR-adjusted *p* value <0.05 were considered differentially expressed.

Transcriptomic data were also analyzed in the context of hierarchically structured network models describing the molecular mechanisms underlying essential biological processes in non-diseased lungs [31], [32]. Leveraging the “cause-and-effect” network models together with NPA algorithms, the gene expression fold changes were translated into differential values for each network node [33], [34]. These were, in turn, summarized into a quantitative NPA measure, and NPA values were aggregated into a biological impact factor; details have been described elsewhere [26], [35].

### 2.7 Proteomics

Proteome alterations were assessed by isobaric tag-based quantification with the iTRAQ® approach (N = 8) (see [7], [36], [23] and protocol on INTERVALS [37]). Samples were processed and analyzed in randomized order. Frozen lung tissue slices were homogenized with a bead-assisted procedure in a Tissue Lyser II (Qiagen, Hilden, Germany) in tissue lysis buffer (BioRad Laboratories, Hercules, CA, USA) before acetone precipitation. Protein precipitates were resuspended in 0.5 M triethylammonium bicarbonate (Sigma-Aldrich, St. Louis, MO, USA), 1 M urea (Sigma-Aldrich), and 0.1% sodium dodecyl sulfate (Sigma-Aldrich). Next, 50-μg aliquots of the suspension were processed by using the iTRAQ® 8-plex labeling procedure in accordance with the manufacturer’s instructions (AB Sciex, Framingham, MA, USA). A trypsin–Lys C mix (Promega, Madison, WI, USA) was added to the samples at a 1:10 ratio (w/w). This was followed by overnight digestion at 37 °C. Trypsin-digested samples were labeled with reporter-ion tags for different exposure groups.

Sample replicates were assigned to different iTRAQ® labeling sets, as described previously [21] – essentially, following a randomized complete block design. For this, separately for each analysis time point, iTRAQ® analysis sets with randomized set and reporter ion channel assignments were defined: Each 8-plex labeling replicate set included one sample of each exposure group and one pooled reference mix combining all samples (note that per default processing, scaling of the reporter ion set was done by the median rather than reference mix value, because of the improved variance properties [38], [39]). All labeled samples that belonged to one iTRAQ® replicate set were pooled and dried in a SpeedVac concentrator (RVC 2–25 CD Plus; Martin Christ, Osterode am Harz, Germany). The samples were desalted by using 0.5-mL bed detergent-removal columns (Pierce, Rockford, IL, USA) and then with 1-cc C18 reversed-phase Sep-Pak columns (Waters, Milford, MA, USA) in accordance with the manufacturers’ protocols. The samples were dried in a SpeedVac evaporator and resuspended in nanoLC buffer A (5% acetonitrile and 0.2% formic acid; Sigma-Aldrich). They were analyzed in random order by using an Easy nanoLC 1000 instrument (Thermo Fisher Scientific, Waltham, MA, USA) connected online to a Q Exactive™ mass analyzer (Thermo Fisher Scientific). Peptides were separated on a 50-cm Acclaim™ PepMap™ 100 C18 LC column (2-μm particle size; Thermo Fisher Scientific) at a flow rate of 200 nL/min, with a 200-min gradient from nanoLC buffer A to 40% acetonitrile with 0.2% formic acid. Each sample was injected twice, with two different analytical methods on the same column (one fast and one sensitive method), as previously described [40]. The outputs of both MS runs were combined as merged mass-lists and interrogated against the mouse reference proteome set (UniProt, version July 2014, canonical isoforms only) by using Proteome Discoverer, version 1.4 (Thermo Fisher Scientific). SequestHT (implemented in Proteome Discoverer) was used as the search tool, and iTRAQ® reporter-ion intensities were determined from Proteome Discoverer. The Percolator node of Proteome Discoverer was used to estimate peptide-level FDR-adjusted *p* values (*q* values).

iTRAQ® peptide-level quantification data were exported and further processed in the R statistical environment [41]. Quantification data were filtered for *q* values <0.01 and “unique” quantification results, as defined by Proteome Discoverer. A global variance-stabilizing normalization was performed with the corresponding Bioconductor package in R [42], [43]. Each iTRAQ® reporter-ion set was normalized to its median, and protein expression values were calculated as the medians of these normalized peptide-level quantification values [39].

For the statistical analysis, a linear model was fitted for each exposure condition and the respective Sham group, *p* values were calculated from moderated *t*-statistics with the empirical Bayes approach [30], and proteins with a Benjamini–Hochberg FDR-adjusted *p* value <0.05 were considered differentially expressed.

### 2.8 Lipidomics

Lipidomics profiles were assessed by using a high-resolution MS/MS shotgun lipidomics protocol (N = 9) (see protocol on INTERVALS [44]).

All samples were analyzed in random order and split into batches of up to 32 samples each. All batches included blank and QC samples (quantified female mouse plasma) to monitor the performance of the quantification workflow.

Frozen lung tissue slices were homogenized by using a Branson W-450D digital sonifier (Branson, Danbury, CT, USA) in 150 mM ammonium bicarbonate buffer. The total protein content of tissue lysates was determined by the Bradford assay. Aliquots of tissue lysates were spiked with an internal standard mixture (PC 15:0/18:1(d7), PE 15:0/18:1(d7), PS) from Avanti Polar Lipids (Alabama, USA) and extracted by the BUME method [45]. High-resolution MS-MS/MS was performed both in positive and negative modes on a Q Exactive™ Plus Orbitrap (Thermo Scientific, Germany) equipped with a Triversa NanoMate robotic interface (Advion, USA). NanoMate parameters were set to 1.25-psi gas pressure and 1.1-kV voltage over a 5-min delivery time. The MS source settings were fixed at a column temperature of 250 °C and S-lens RF level of 65.0. The MS method for the positive mode involved 1 min of full scan covering the *m*/*z* range from 550 to 1000 at 140,000 resolution, with 1E6 automated gain control, a maximum injection time of 50 ms, and a lock mass of 680.48022. The 1- to 5-min DIA MS/MS acquisition was triggered with first mass fixed at 250 *m*/*z*; resolution of 17,500; automated gain control of 1E5; and a maximum injection time of 64 ms at 20 NCE. An isolation window of 1 *m*/*z* was set, with the inclusion mass list starting from 550 to 1000 with a mass step of 1 Da. The MS source settings were fixed at a column temperature of 250 °C and S-lens RF level of 65.0. The MS method for the negative mode involved 1 min of full scan covering *m*/*z* range from 400 to 940 at 140,000 resolution, with 1E6 automated gain control, a maximum injection time of 50 ms, and a lock mass of 529.46262. Then for 1 to 5 min, DIA MS/MS acquisition was triggered with first mass fixed at 150 *m*/*z*; resolution of 17,500; automated gain control of 1E5; and a maximum injection time of 64 ms at 35 NCE, with the inclusion mass list starting from 400 to 940 with mass step of 1 Da.

Raw files from the positive and negative modes were converted to mzML by using PeakByPeak software (SpektroSwiss, Switzerland), which provides automated noise subtraction. The converted files were processed with Lipid Xplorer, v.1.2.7 [46]. Lipid identification was performed by MS/MS, and quantification of identified species was performed by using the MS level. Different lipid species of PC, PE, PS, phosphatidylinositol (PI), phosphatidic acid (PA), phosphatidylglycerol (PG), sphingomyelin (SM), DAG, and TAG were listed with the sum of fatty acyl groups (e.g., PC 32:0). Lyso-PC and lyso-PE are abbreviated as LPC and LPE, respectively, and sterol/cholesteryl esters are abbreviated SE. Ether-linked phospholipids are shown as PCO (alkyl) and PEO (alkyl).

The final lipid concentration was normalized per amount of total protein. A linear model was fitted for each exposure condition and the corresponding air-exposed group, and *p* values from a *t*-statistic were calculated for log_2_-transformed data [30]. The Benjamini–Hochberg FDR method was used to correct for multiple testing effects. Lipids with an adjusted *p* value <0.05 were considered differentially abundant.

### 2.9 Metabolomics

Metabolomics analysis was performed by Metabolon, Inc. (Research Triangle Park, NC, USA) (N = 9). Frozen right lung lobe tissue samples were analyzed by using the Metabolon global untargeted biochemical profiling platform. An earlier version of the profiling platform was described by Evans et al. [47]. Briefly, metabolites were extracted from each sample (normalized by tissue weight) with methanol and analyzed by using four different MS-based methods: two separate reverse-phase/ultra-performance LC-MS/MS (UPLC-MS/MS) methods with positive-ion mode electrospray ionization (ESI), one for analysis by reverse-phase/UPLC-MS/MS with negative-ion mode ESI, and one for analysis by hydrophilic interaction LC/UPLC-MS/MS with negative-ion mode ESI. Under a strict quality-controlled process, raw data were extracted, and peaks were identified by using Metabolon's proprietary software. The peaks were quantified based on the area under the curve. By following the common approach taken by Metabolon [48], [49], missing values were assumed to be missing because of low abundance and were imputed as the minimum value separately for each metabolite. A linear model was fitted for each exposure condition and the corresponding air-exposed group, and *p* values from a *t*-statistic were calculated [30]. The Benjamini–Hochberg FDR method was used to correct for multiple testing effects. Metabolites with an adjusted *p* value <0.05 were considered differentially abundant.

### 2.10 Multi-omics data analysis

MOFA was performed with the corresponding package (version 0.99.8) in the R statistical environment (version 3.5.1) by using the default model and train options [50]. The mixOmics R package (version 6.6.1) was used to perform sGCCA by using the block sparse partial least squares method in canonical mode, with a fully connected design matrix between the five omics data modalities [51]. The sparsity constraint was set to 30 variates per component.

For association network analysis, an aggregated network was compiled from the KEGG database (metabolite/enzyme links) [52], STRING database (gene/protein links, version 10.5) [53], and mirTarBase (micro-RNA/gene links, version July 2018) [54]. The STRING database network was filtered for high-confidence links (combined score >0.7), and accepted miRNA interactions had to be reported by at least two publications and two non-high-throughput methods. Edge weights for interactions were set to 0.1 for the KEGG database and mirTarBase and to (1 − combined score) + 0.1 for the STRING database.

The PCSF graph optimization approach [55] was used to derive the association network for LF 1 from the MOFA model (PCSF R package, version 0.99.0) by using the aggregated network defined above. A maximum of 200 terminal molecules for each data modality were considered and filtered for those with normalized absolute weights two-fold higher than expected by chance, with the normalized absolute weights set as prizes. A grid search was performed (fixed parameter µ = 0.0005; ω = 0.2–1; β = 100–2000) (Supplementary Fig. 7 [Supplementary file 1]), and the final network was selected on the basis of the saturation of percent covered with a decent number of trees (µ = 0.0005; ω = 0.6; β = 1000). To create the final network, 10 runs with noise to the edge costs were combined (r = 0.1). The network was clustered using the edge-betweenness clustering algorithm.

Gene set enrichment analysis was performed by using the fgsea algorithm (fgsea R package, version 1.8.0) [56], and gene set overrepresentation analysis was performed by using Fisher’s exact test, both with the piano package (version 1.22.0) for R [57]. Enrichment of gene sets from the Reactome database was evaluated (version April 2018) [58].

### 2.11 Availability of source code and requirements

The R script (Rmd file), R functions, and R data objects used for conducting the presented multi-omics analyses are available from a github repository:

• Project name: MouseLungMultiOmics
• Project home page: https://github.com/philipmorrisintl/MouseLungMultiOmics
• Operating system(s): Platform-independent (tested on UNIX only)
• Programming language: R
• Other requirements: KEGG database license
• License: GNU General Public License v2.0 or later (code), CC BY 4.0 (data)

In addition, the rendered script output in PDF format is available as Supplementary file 3.

### 2.12 Availability of supporting data and materials

The raw, processed, and contrast data for the analyzed omics datasets are available on INTERVALS (https://www.intervals.science/) [59], [24] (Table 1).

**Table 1:** Multi-omics datasets.

Data type	Time points [months]	N	INTERVALS	Other repository
Proteomics	3, 4, 6	8	processed [72] contrast [73]	Pride [60]: PXD010875
mRNA transcriptomics	3, 4, 6	9	raw [74] processed [75] contrast [76]	ArrayExpress [61]: E-MTAB-7444
miRNA transcriptomics	3, 4, 6	9	raw [77] processed [75] contrast [76]	ArrayExpress [61]: E-MTAB-7892
Metabolomics	3, 6	9	processed [78] contrast [79]	MetaboLights [62]: MTBLS158
Lipidomics	3, 4, 6	9	raw [80] processed [81] contrast [82]	–

The mass spectrometry proteomic data are available from the ProteomeXchange Consortium through the PRIDE partner repository (http://www.ebi.ac.uk/pride/archive/) [60], with the identifier PXD010875.

The transcriptomics data are available from ArrayExpress (www.ebi.ac.uk/arrayexpress) [61], with accession numbers E-MTAB-7444 (mRNA) and E-MTAB-7892 (miRNA).

The metabolomics data have been deposited in the MetaboLights repository (https://www.ebi.ac.uk/metabolights/) [62], with the identifier MTBLS158.

The lipidomics data are available on INTERVALS (https://www.intervals.science/) [24], [59].

## 3 Results

### 3.1 Overview on generated multi-omics datasets to assess lung exposure effects

Here, we present the data and analysis results of a multi-omics systems toxicology study to investigate the impact of aerosols from CHTP 1.2 and THS 2.2, compared with CS, on the lungs of ApoE^−/−^ mice. Our multi-omics analysis included mRNA and microRNA (miRNA) transcriptomics, proteomics, metabolomics, and lipidomics studies. The ApoE^−/−^ mouse model is commonly used for atherogenesis [63], especially for investigating smoking-related atherosclerosis [64], [65], [66], [67], as well as CS-induced lung inflammation and emphysema [67], [68], [69], [70].

With this, this report expands and complements our overview publication on this study, which contains the full exposure characterization and additional non-omics endpoints, including assessment of cardiovascular effects [23].

**Fig. 1:**
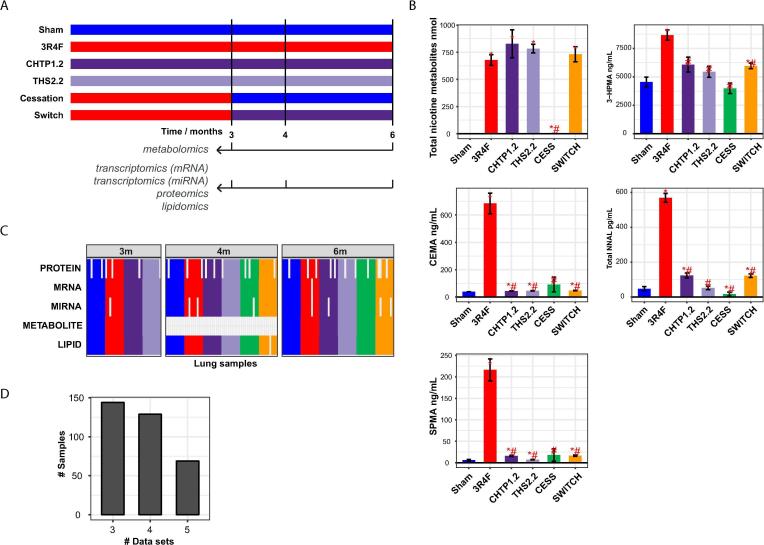
Multi-omics analysis of lung exposure effects. (A) Design of a 6-month inhalation study on ApoE^−/−^ mice. Other endpoints and additional details on this systems toxicology study are reported in Phillips et al. [23]. (B) Exposure markers measured in urine: 3-hydroxypropylmercapturic acid (3-HPMA), a biomarker of acrolein uptake; 2-cyanoethylmercapturic acid (CEMA), a biomarker of acrylonitrile uptake; total 4-(methylnitrosamino)-1-(3-pyridyl)-1-butanol (NNAL), a biomarker of 4-(methylnitrosamino)-1-(3-pyridyl)-1-butanone uptake; and S-phenylmercapturic acid (SPMA), a biomarker of benzene uptake. Note that the high basal levels of 3-HPMA, including in the Sham group, can be explained by endogenous acrolein production [71]. Data are shown as mean ± standard error of the mean. Statistical comparisons: *p value versus Sham <0.05; #p value versus 3R4F <0.05; N = 10–12. (C) Lung samples obtained and analyzed from the same animals across the five omics analyses. A colored cell in the matrix indicates that a lung sample from an animal was analyzed successfully for the given omics dataset. Colors of sample groups as in panel B. Analyses performed with a planned N = 9 for mRNA/miRNA transcriptomics, lipidomics, and metabolomics and with a planned N = 8 for proteomics. Four-month samples were not submitted for metabolomics analysis. (D) Distribution plot showing the number of shared samples across a given number of omics datasets.

In this study, groups of mice were exposed for up to 6 months to filtered air (Sham), 3R4F CS, CHTP 1.2 aerosol, or THS 2.2 aerosol (Fig. 1A; for further details see [23], [24]). In addition, the mice were exposed to 3R4F CS for 3 months before changing the exposure conditions to filtered air (Cessation) or CHTP 1.2 aerosol (Switch). The exposure conditions were matched by nicotine concentration in CS and aerosols (28 µg nicotine/L), reflected by similar levels of total nicotine metabolites in urine, whereas HPHC markers were substantially lower in groups exposed to the candidate MRTP aerosols than in those exposed to CS (Fig. 1B) [23].

For multi-omics analysis, lung samples were obtained from dedicated groups of mice after 3, 4, and 6 months of exposure. To support comprehensive integrative analysis of animal-level matched samples, left lung slices were assigned for transcriptomics (mRNA and miRNA), proteomics, and lipidomics analyses, and a right lung lobe was assigned for metabolomics analysis. Transcriptomics analysis was performed by using Affymetrix microarrays, and quantitative proteomics analysis was performed by isobaric-tag labeling (iTRAQ®) and liquid chromatography (LC) coupled with tandem mass spectrometry (MS/MS) [36]. Metabolomics analysis was also LC-MS/MS-based [47], and shotgun lipidomics analysis was conducted by using a direct-injection high-resolution MS/MS method. In total, we captured approximately 17,500 mRNAs, 5000 proteins, 670 metabolites, 400 lipids, and 360 miRNAs in the lung tissues.

Fig. 1C summarizes the successfully analyzed lung samples for each omics modality. Note that metabolomics analysis was conducted only for the 3- and 6-month time points in order to limit the number of samples for this analysis, which was performed by an external provider. Proteomics analysis was conducted in eight and the other omics analyses in nine replicates. In addition, a few individual samples were excluded during quality control (QC). Overall, we generated a comprehensive multi-omics dataset for the lungs, which includes up to five data modalities for up to 144 samples: Three modalities were measured for 144 samples, four for 129 samples, and all five modalities for 69 samples (Fig. 1D, Table 1).

Beyond the analysis of this multi-omics dataset presented here, we expect that these data can support the development of novel data integration approaches in the future, and the data can be mined for additional biological insights (e.g., on lung exposure and stress responses).

### 3.2 CS exposure substantially affects the lungs across the five molecular layers

**Fig. 2:**
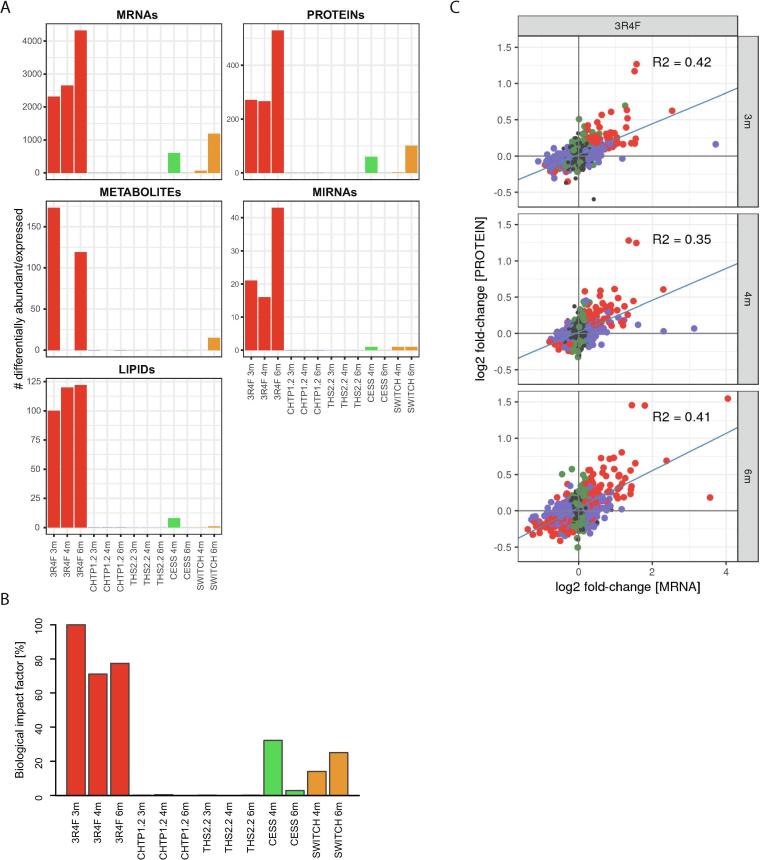
Molecular exposure responses in the lungs across the five data modalities. (A) Numbers of differentially expressed/abundant mRNAs, proteins, metabolites, miRNAs, and lipids per group relative to Sham exposure (FDR-adjusted p value <0.05). (B) Evaluation of biological impact on lung tissue by using a causal network enrichment approach based on transcriptomics data. RBIFs are represented for each group versus Sham comparisons. See Supplementary Fig. 1 for individual causal network responses [Supplementary File 1]. (C) Correlation between gene and protein expression/abundance fold changes for 3R4F versus Sham at the 3-, 4-, and 6-month time points. Dots indicate the significance of differential expression: red, both protein and mRNA; blue, mRNA only; green, protein only; grey, not significant. Blue line represents fit from linear model; R2, coefficients of determination. (For interpretation of the references to color in this figure legend, the reader is referred to the web version of this article.)

To obtain a high-level overview of the exposure impact across the five assessed molecular layers, we calculated differential abundance profiles, comparing the abundance for each quantified biomolecule in the exposure groups with that in the corresponding Sham groups (false discovery rate [FDR]-adjusted *p* value <0.05; Fig. 2A; Supplementary file 2). Supporting a substantial effect on all assessed molecular layers, the groups exposed to 3R4F CS showed a marked differential expression/abundance response across the five omics data modalities, with a maximum of 4324 affected mRNAs, 529 affected proteins, 173 affected metabolites, 43 affected miRNAs, and 122 affected lipids. In contrast, exposure to CHTP 1.2 and THS 2.2 aerosols at matched nicotine concentration to 3R4F CS yielded only a single differentially expressed biomolecule: *C1qtnf4* mRNA was downregulated following THS 2.2 exposure at the 6-month time point (*C1qtnf4* not detected by proteomics analyses.). Both the Cessation and Switch (to CHTP 1.2 aerosol) groups demonstrated differentially abundant biomolecules across all five omics datasets. However, compared with 3R4F CS, these were less numerous, and a discrepancy in the time course between cessation and switching was noted when comparing the number of differentially expressed/abundant mRNAs and proteins at the 4- and 6-month time points.

To complement these differential expression profiles—which are sensitive to the FDR threshold—we quantitatively evaluated the perturbation of the mRNA transcriptome by using a previously published causal network enrichment approach (see [83], [33] for a more detailed explanation of this approach). For a collection of causal biological network models relevant to lung biology [31], the degree of network perturbation caused by a given exposure—or network perturbation amplitude (NPA)—is calculated on the basis of measured transcriptomic changes (Supplementary Fig. 1 [Supplementary file 1]). Subsequently, NPAs are aggregated to derive the overall relative biological impact factors (RBIF) [84] (Fig. 2B). Overall, this evaluation confirmed the trends observed for the differential expression/abundance profiles, with a predicted biological impact of 3R4F CS, reduced impact upon cessation and switching, and low to absent perturbation upon exposure to CHTP 1.2 and THS 2.2 aerosols.

To evaluate how the different data modalities relate to each other, we first correlated the mRNA and protein responses to 3R4F CS exposure (Fig. 2C). The coefficients of determination (R2) ranged from 0.35 to 0.42, indicating a clear association but inability of the different measurements to fully determine others. These R2 values were in the expected range, based on previous comparisons, and reflect the different regulatory mechanisms acting at the protein and mRNA levels as well as technical variabilities [85]. Importantly, with respect to data integration, this observation supports that these two data modalities can confirm and complement each other.

To further assess the multivariate molecular response captured by each data modality, we evaluated the main directions in the data by principal component analysis (PCA; Supplementary Fig. 2 [Supplementary file 1]). For both mRNA transcriptomics and proteomics analyses, the first principal component clearly captured the biological response to 3R4F CS exposure (11.1% and 18.3% explained variance, respectively), whereas the separation on the second principal component was not associated with specific exposure groups. Metabolomics analysis also appeared to capture the 3R4F exposure effects on the first principal component but with less separation. Lipidomics analysis captured the 3R4F CS exposure effect *only* on the second principal component, whereas no clear group separation was apparent for miRNA transcriptomics analysis on the first and second principal components. Thus, the PCA findings for the individual datasets support the idea that 3R4F CS exposure drives a clear separation of the samples on the principal multivariate directions; however, joint evaluation and further interpretation across all five data modalities require an integrative multivariate approach.

### 3.3 Integrative multi-omics response profiles

Multi-omics factor analysis (MOFA) was developed as a framework for the unsupervised integration of multi-omics datasets [50]. Briefly, data matrices with shared samples are decomposed into one latent factor (LF) matrix and weight matrices for each data modality. Similar to PCA for single-omics data, MOFA aims to identify an interpretable low-dimensional representation of the data, with LFs capturing the major sources of variation across the data modalities. A benefit of the MOFA implementation is that it efficiently handles missing values, making it especially applicable to the multi-omics lung dataset (Fig. 1C).

**Fig. 3:**
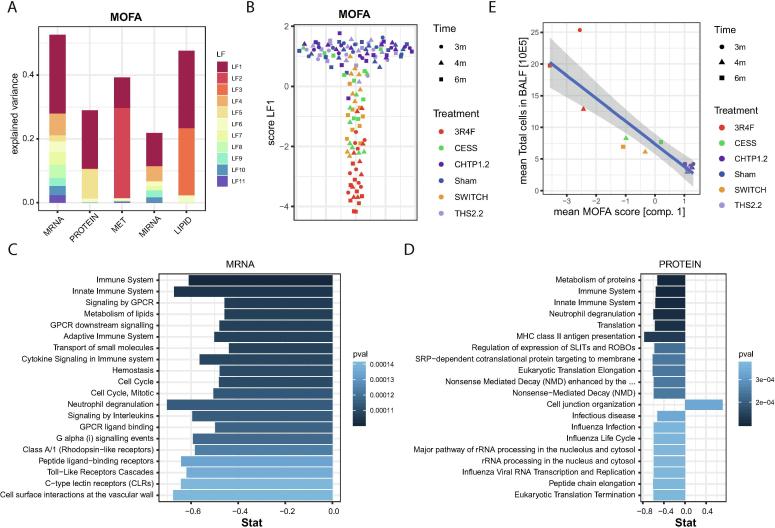
Integrative multi-omics LF model. An integrative LF model for all five data modalities was derived by MOFA [50]. (A) Fraction of explained variance for each data modality and each discovered LF of the MOFA model. (B) Score plot for LF 1. The LF 1 score for each sample is given on the y-axis. (Random spread on x-axis for visualization purposes.) The shape of the data points indicates the time point, and the colors indicate the treatment or exposure times (see key). (C, D) Top 20 enriched gene sets (by p value) for the weight vectors of LF 1 for the mRNA transcriptomics (C) and proteomics (D) datasets. (E) Comparison between the mean MOFA score of LF 1 and the total number of (immune) cells in BALF for each treatment and time-point group (see key). Linear fit with the 95% confidence interval band is shown (blue line, grey area). (For interpretation of the references to color in this figure legend, the reader is referred to the web version of this article.)

LF 1 substantially explained the observed variance across all five data modalities and was especially associated with the majority of overall explained variance for the proteomics data (Fig. 3A). In contrast, LF 2 and LF 3 mostly explained the observed variance in the metabolomics and lipidomics data, respectively. Pairwise score plots for LFs 1–10, which represent most of the variance explained by the MOFA model, revealed that only LF 1 was directly associated with the exposure groups in the experimental design, reflecting the multi-omics exposure response to 3R4F CS (Supplementary Fig. 3 [Supplementary file 1]). Fig. 3B shows the exposure group separation on LF 1, where 3R4F samples are clearly separated from the cluster of overlapping samples from the Sham, CHTP 1.2, and THS 2.2 groups, whereas samples from the Cessation and Switch groups had intermediate LF 1 scores. Overall, this multi-variate and multi-omics perspective is consistent with the trends observed for the individual modalities, supporting low to absent molecular exposure effects for CHTP 1.2 and THS 2.2 aerosols compared with 3R4F CS, and intermediate (remaining) molecular alterations for cessation and switching to CHTP 1.2.

To further support the results obtained with the MOFA approach, we complemented it with another multivariate, multi-omics data reduction approach, sparse generalized canonical correlation analysis (sGCCA) [86]. This method factorizes each data matrix into a separate score and loading matrix, identifying factors with maximal covariance across the considered data modalities. In agreement with the MOFA model, sGCCA identified a first factor that demonstrated a similar group separation as LF 1 across all five data modalities (Supplementary Fig. 4A–C [Supplementary file 1]). Of note, the selected variables for the first factor generally were also highly ranked in the MOFA model (Supplementary Fig. 4D [Supplementary file 1]).

Toward functional interpretation of LF 1 of the MOFA model, we first conducted gene set enrichment analysis on the corresponding weight vectors for the transcriptomics (Fig. 3C) and proteomics (Fig. 3D) data by using the Reactome pathway collection [58]. Strikingly, the majority of gene sets most significantly associated with LF 1 were immune-related. CS is known to affect lung immune responses [87], which have been associated with COPD development [87], [88], and strong immune responses to 3R4F CS have been observed in previous mouse and rat inhalation studies (e.g., [16], [89]). In the current study, 3R4F CS also induced a substantial immune response in the lungs, as assessed by the quantification of immune cells and immunoregulatory proteins in bronchoalveolar lavage fluid (BALF) and histopathological observations in the lungs [23]. In a direct comparison between cellular and molecular assessments, we observed a tight association between the results (Fig. 3E), further confirming the group differences and supporting that the lung immune response critically contributes to LF 1.

### 3.4 Complex interconnected molecular response

**Fig. 4:**
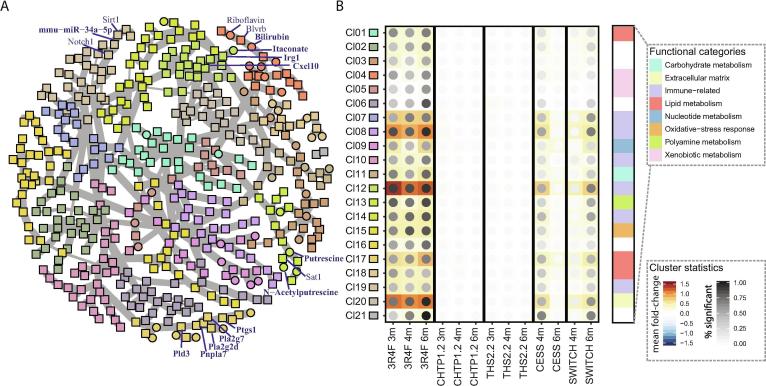
Multi-omics response network. (A) Aggregated gene–protein–metabolite–miRNA network for LF 1 constructed by using the PCSF algorithm [55]. Nodes represent molecules, and edges represent their interactions. Node colors reflect the identified clusters/communities in the network (see cluster key in panel B). Select molecules are labeled. See the interactive graph in Supplementary Fig. 5 for details [Supplementary File 1]. (B) Expression profiles for identified clusters (Cl). Mean log_2_ fold changes are color-coded, and the percentage of molecules with significant differential expression/abundance in a cluster is indicated by the darkness of the dots (see key). In addition, the broad functional classifications for the clusters are indicated (color bar on right side).

To expand the functional analysis and identify direct molecular associations across the data modalities, we conducted a biological network analysis for LF 1. For this, we leveraged an aggregated gene/protein–metabolite–miRNA network and the Prize-Collecting Steiner Forest (PCSF) algorithm [55] to derive a biological association network between the most influential molecules for LF 1 (Fig. 4A; see Supplementary Fig. 5 for an interactive version [Supplementary file 1]). Network cluster analysis and annotation indicated that this network covered diverse biological functions, prominently including clusters linked to immune response, xenobiotic/oxidative stress response, lipid metabolism, and extracellular matrix (Supplementary Table 2 [Supplementary file 1]). Fig. 4B visualizes the exposure response profiles for the identified clusters. As expected, these profiles were consistent with the aggregated trend observed for LF 1, but they also highlighted the fraction of individually significantly differentially expressed/abundant molecules in each cluster.

**Table 2:** Metabolites and miRNAs implicated in exposure-related immune responses.

Molecule	Exposure association	Immune association [examples]
Itaconate	DA, LF1, BN	Immune response regulator in macrophages [92], [93], [94], [95]
Polyamines (putrescine, acetyl-putrescine, acetyl-spermidine)	DA, LF1, BN	Immune regulatory functions, including in lymphocyte and macrophage activation [98], [99], [107]
Dihydrobiopterin, biopterin	DA, LF1	Dihydrobiopterin levels increased in activated inflammatory macrophages [108]
Quinolinate	DA, LF1	Kynurenine pathway metabolite associated with immune activation [109], [110]
Methylsuccinate	DA, LF1	Likely itaconate product [111]
Prostaglandin D2	LF1, BN	Lipid mediator involved in immune activation [112]
mmu-miR-146a	DE, LF1, BN	Negative regulation of immune activation, role in myeloid cells [100]
mmu-miR-21a	DE, LF1, BN	Upregulated in allergic airway inflammation [113]
mmu-miR-2137	DE, LF1	Possible anti-inflammatory role in macrophages [106]

DA, differential abundance; DE, differential expression; LF1, latent factor 1 of the multi‐omics factor analysis model; BN, biological association network.

To gain further insights into the exposure response, we explored the identified functional categories in more detail, including the highlighted interactions across molecular layers (Fig. 4A), as detailed in the following sections.

### 3.5 3R4F CS-induced immune response encompasses metabolite and miRNA changes

We focused on the immune response first. In both humans and rodents, CS triggers an extensive immune response in the lungs [16], [87], [88], [89], and the identified LF 1 of the MOFA model appeared to capture this immune response in the current study (Fig. 3, Fig. 4). In previous studies, we highlighted several aspects of this immune response, including macrophage polarization and lipid signaling [21], [90], [91]. In the context of this multi-omics study, we specifically focused on the involvement of the additional molecular layers.

**Fig. 5:**
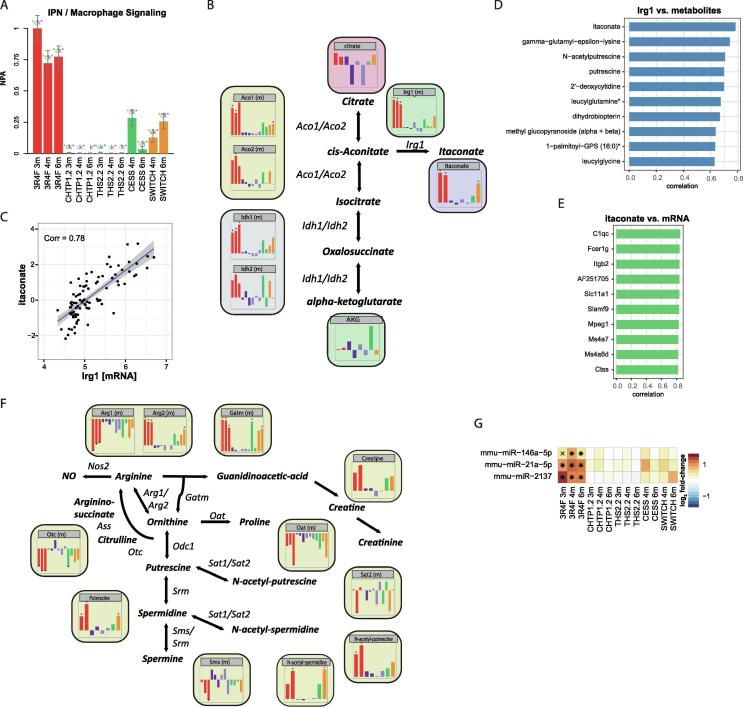
Metabolite and miRNA changes associated with 3R4F CS-induced immune response. (A) Network enrichment analysis of the macrophage signaling network. The bars show the overall NPA based on transcriptomics data; error bars show the 95% confidence interval. Three statistical measures are shown: the red star indicates statistical significance with respect to biological replicates; the green star (o statistic) indicates significance with respect to permutation of genes downstream of the network nodes; and the blue star (k statistic) indicates significance with respect to permutation of the network topology (p < 0.05). (B) Itaconate metabolic pathway. Bar charts show log_2_ fold-change responses versus Sham, ordered and colored as in panel A. (C) Correlation between Irg1 mRNA expression and itaconate abundance. Blue line shows fit from the linear model, with the 95% confidence interval band in grey. The correlation coefficient (Corr) is indicated. (D) Top 10 correlations of Irg1 mRNA expression against all metabolites. (E) Top 10 correlations of itaconate abundance against all mRNAs. (F) Polyamine pathway, as in panel B. (G) Expression profiles for selected immune-related miRNAs. Log_2_ fold-changes versus Sham are color-coded, and statistical significance is indicated: *FDR-adjusted p value <0.01; ^X^FDR-adjusted p value <0.05. (For interpretation of the references to color in this figure legend, the reader is referred to the web version of this article.)

The network enrichment approach based on transcriptomics data supported perturbation of the macrophage activation network upon CS exposure [31] (Fig. 5A), consistent with the observed increase in macrophage numbers in BALF and lung tissues upon CS exposure in this study [23]. In the molecular association network, the metabolite itaconate was linked to aconitate decarboxylase (Irg1; also known as Acod1) in an immune-response-associated cluster comprising many chemokines (Fig. 4A). Itaconate has been implicated in the immune response of macrophages [92], [93], [94], with Irg1 catalyzing its synthesis from *cis*-aconitate [95]. Here, we observed a strong increase in the abundance of itaconate and Irg1 upon 3R4F CS exposure (Fig. 5B). Furthermore, 3R4F CS exposure was associated with a significant increase in the abundance of aconitases (Aco1 and Aco2) and isocitrate dehydrogenase 1 (Idh1), supporting the potential for increased flux through this part of the citric acid cycle. Itaconate and *Irg1* mRNA abundance/expression were clearly correlated (Fig. 5C), with itaconate being the metabolite with the highest correlation with Irg1 (Fig. 5D). Of note, the mRNAs that showed the highest correlation with itaconate included several other immune- and macrophage-related genes, such as the complement protein gene *C1qc* [96], macrophage expressed gene 1 (*Mpeg1*), and *Cd300c* (GenBank: AF251705) [97] (Fig. 5E).

In the molecular association network, the polyamines putrescine and N-acetyl-putrescine were linked in a cluster that also included arginase (Arg2) (Fig. 4A). Polyamines, including spermidine and spermine (and their acetylated variants), are synthesized from ornithine (Fig. 5F). Polyamines have been implicated in immune regulatory functions, including lymphocyte and macrophage activation [98], [99]. Here, we observed a significant increase in the abundance of putrescine, N-acetyl-putrescine, and N-acetyl-spermidine upon 3R4F CS exposure. 3R4F CS exposure increased the abundance of mitochondrial arginase (Arg2) and glycine amidinotransferase (Gatm), which likely contributes to the flux toward polyamine synthesis. It also caused a decreased abundance of cytoplasmic arginase (Arg1) and no significant differences in the abundance of ornithine decarboxylase (Odc1), spermidine synthase (Srm), and spermine synthase (Sms). However, decreased abundance of ornithine carbamoyltransferase (Otc) and ornithine aminotransferase (Oat) could limit alternative fluxes of ornithine toward proline and citrulline, respectively, further promoting polyamine synthesis upon 3R4F CS exposure.

In addition to metabolites, the molecular association network also implicated miRNAs in immune-related functions. The miRNA mmu-miR-146a was found to be associated with immune-related signaling molecules Traf6 and Irak1 and showed strong upregulation upon 3R4F CS exposure (Fig. 5G). In fact, mmu-miR-146a has been implicated in the regulation of immune responses [100] and has recently been linked to the observed imbalance of Th1/Th2 lymphocytes in the lungs of mice exposed to airborne fine particulate matter [101]. The miRNA mmu-miR-21a was also strongly associated with LF 1 and found associated with programmed cell death 4 (Pdcd4) and SMAD family member 7 (Smad7) in the network. Pdcd4 and Smad7, as signal transduction regulators, are not only linked to immune cells but also have reported roles in immune regulatory processes, including via the interleukin (IL)-6/Stat3 [102] and transforming growth factor beta pathways [103]. Indeed, mmu-miR-21a has been identified as a negative immune regulator in mouse liver regeneration (via nuclear factor kappa B [NF-κB] inhibition) [104] and macrophage response in peritonitis [105]. Finally, mmu-miR-2137 was strongly induced upon 3R4F CS exposure; it was the second most strongly associated miRNA (after mmu-miR-146a) with LF 1. Compared with mmu-miR-146a and mmu-miR-21a, mmu-miR-2137 is less studied and had no identified association in the molecular association network in our study. However, an mmu-miR-2137 inhibitor was recently found to increase tumor necrosis factor alpha (TNFα) secretion and decrease IL-10 secretion in macrophages in the context of *Porphyromonas gingivalis* infection, suggesting an anti-inflammatory role for mmu-miR-2137 [106].

Overall, the integrative analysis yielded further insights into the complex lung immune response to 3R4F CS exposure that occurs simultaneously on several molecular layers (Table 2). In contrast, there were no signs of an immune response against CHTP 1.2 and THS 2.2 aerosol exposure.

### 3.6 Cellular stress responses

**Fig. 6:**
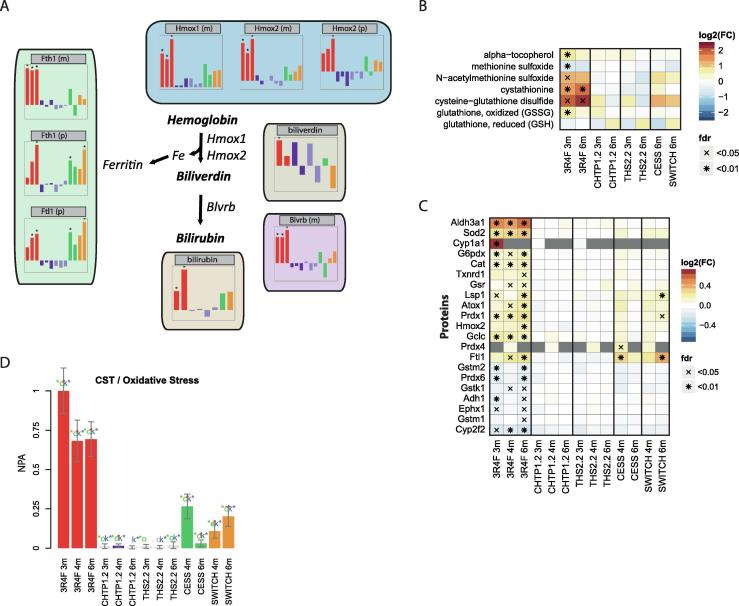
Effects of the 3RF reference cigarette and heat-not-burn tobacco products on oxidative stress. (A) Activation of the hemoglobin–biliverdin–bilirubin pathway. Representation as in Fig. 5B. (B) Oxidative-stress-related metabolites. Log_2_ FC versus Sham are color coded, and statistical significance is indicated: *FDR-adjusted p value <0.01; ^X^FDR-adjusted p value <0.05. (C) Expression of oxidative-stress-related proteins. (D) Perturbation of the oxidative stress network. The bars show the overall NPA based on transcriptomics data. See Fig. 5A for details.

CS exposure is associated with extensive cellular stress responses in the lungs, including oxidative stress response caused by direct exposure and induced generation of reactive oxygen species (ROS) [21], [90], [114], [115], [116]. Likely related to oxidative stress response, the molecular association network highlighted the exposure effect on bilirubin and its synthesizing enzyme, biliverdin reductase (Blvrb), both of which were significantly increased in the 3R4F CS-exposure group but not in the other exposure groups (Fig. 6A). Biliverdin, an intermediate in the bilirubin synthesis pathway, did not show a clear response pattern across the groups (tendency of increase at 4 months), whereas the abundance of heme oxygenases Hmox1 and Hmox2 was increased by 3R4F CS. Hmox enzymes oxidize and cleave hemoglobin to biliverdin, and the released iron is captured by the iron-storage protein ferritin (composed of Fth1 and Ftl1 subunits), which was also increased in abundance after 3R4F CS exposure. Several studies have described the roles of the heme–biliverdin–bilirubin pathway in oxidative stress responses, including production of the antioxidants biliverdin and bilirubin and depletion of the oxidant heme [117].

**Fig. 7:**
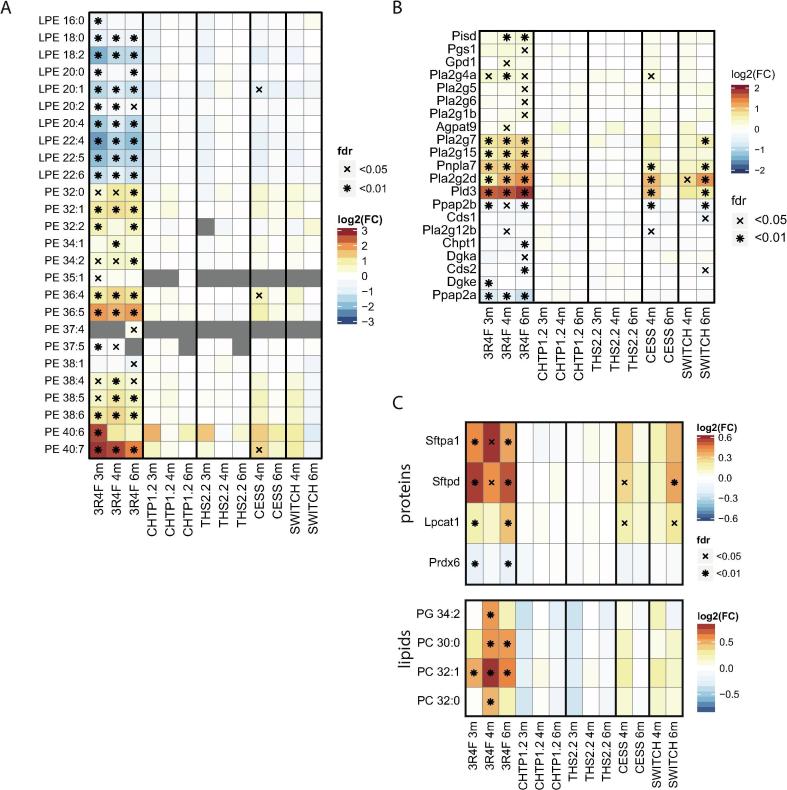
Effects of the 3RF reference cigarette and heat-not-burn tobacco products on lipid metabolism. (A) Abundance of PEs and LPEs. Log_2_ FC versus Sham are color coded, and statistical significance is indicated: *FDR-adjusted p value <0.01; ^X^FDR-adjusted p value <0.05. (B) Expression of genes involved in glycerophospholipid metabolism. (C) Abundance profiles of surfactant proteins and candidate surfactant lipids. PC, phosphatidylcholine; PG, phosphatidylglycerol.

In addition to the heme–biliverdin–bilirubin pathway, other metabolites linked to oxidative stress responses were affected in the 3R4F CS-exposed groups (Fig. 6B). They included a prominent increase in cysteine-glutathione disulfide and cystathionine levels [118]. Furthermore, (acetylated) oxidized methionine, oxidized glutathione, and the antioxidant alpha-tocopherol were significantly affected by 3R4F CS exposure only at the 3-month time point.

An oxidative stress response was also observed in the proteome (Fig. 6C) and transcriptome (Fig. 6D). The protein response included increased abundance of superoxide dismutase 2 (Sod2), catalase (Cat), thioredoxin reductase 1 (Txnrd1), antioxidant 1 copper chaperone (Atox1), and X-linked glucose-6-phosphate dehydrogenase (G6pdx). The latter indicated flux through the pentose-phosphate pathway toward nicotinamide adenine dinucleotide phosphate generation [21]. The transcriptome response was evaluated in the context of the oxidative stress network model (Fig. 6D), which further supported the conclusions about oxidative stress response from other data modalities.

Overall, the multiple molecular layers supported a general oxidative response profile in the lungs, with strong induction by 3R4F CS exposure, low to absent levels for CHTP 1.2 and THS 2.2, and intermediate (remaining) levels for cessation and switching to CHTP 1.2.

### 3.7 Effects on lipid metabolism

Consistent with the findings of our previous ApoE^−/−^ mouse study [21], 3R4F CS exposure substantially affected the lung lipidome (Fig. 2A). The biological network analysis highlighted three lipid-related clusters affected by the exposure: One cluster contained genes/proteins involved in lipid biosynthesis (cluster #1), one cluster contained phosphoglycerolipids and associated enzymes (cluster #17), and one cluster was related to cholesterol metabolism (cluster #18) (Fig. 4A; Supplementary Fig. 5 [Supplementary file 1]).

The broad effects of 3R4F CS exposure on the lung lipidome were evident at the lipid-class level, with significant changes in several lipid classes including glycerolipids (e.g., diacylglycerol [DAG] and triacylglycerol [TAG]) and glycerophospholipids (e.g., phosphatidylcholine [PC] and phosphatidylethanolamine [PE]) (Supplementary Fig. 6 [Supplementary file 1]). Interestingly, whereas diacylated glycerophospholipids (e.g., PC and PE) showed generally increased levels upon 3R4F CS exposure, the levels of monoacylated glycerophospholipids (e.g., lyso-PC [LPC] and lyso-PE [LPE]) were generally decreased. This was especially apparent for PE and LPE lipids, which, irrespective of their conjugated fatty acids, were significantly increased/decreased in concentration in the 3R4F CS-exposure groups at all three time points (Fig. 7A). Concomitantly, 3R4F CS affected the expression of several genes involved in glycerophospholipid metabolism (Fig. 7B), supporting a coordinated lipidome response across the different molecular layers. For several of these genes, the biological network approach suggested possible functional links. Phosphatidylserine decarboxylase (Pisd) converts phosphatidylserine (PS) to PE and is the key enzyme that maintains the (elevated) PE levels of the inner mitochondrial membrane [119]. Patatin-like phospholipase domain containing 7 (Pnpla7) is a lysophospholipase, which localizes to the endoplasmic reticulum and lipid droplets. Pnpla7 was specifically associated with LPC lipolysis but also acts on LPE and lyso-PS [120]. Phospholipase D3 (Pld3) is an intriguing case, because the network approach suggested a link to PC lipids, but the functional role of the protein encoded by *Pld3* is still unknown. However, recently, Gavin et al. found that Pld3 possesses 5′ exonuclease activity and found *Pld3* expression by macrophages controls toll-like receptor (Tlr) 9 activation, possibly by degrading nucleic acids [121].

Pulmonary surfactant is a lipid–protein complex crucial for lung homeostasis. It stabilizes the alveolar structure by reducing surface tension and also acts as a component of the innate lung immune response [122]. Consistent with our previous study [21], surfactant metabolism was affected by 3R4F CS exposure at both the protein and lipid levels, whereas no significant effects were detected for the CHTP 1.2 and THS 2.2 groups, and lower effects were observed upon cessation and switching to CHTP 1.2 (Fig. 7C). 3R4F CS exposure increased the abundance of the surfactant proteins Sftpa1 and Sftpd, which are involved in immune defense and surfactant homeostasis [123], whereas no change in abundance was detected for the structural surfactant proteins Sftpb and Sftpc. Lysophosphatidylcholine acyltransferase (Lpcat1), a critical enzyme for synthesis of surfactant lipids in mice [124], [125], also increased in abundance, together with candidate surfactant lipids [21], whereas the abundance of Prdx6, which acts as a lysosomal-type phospholipase A2 for surfactant lipids, decreased significantly [126].

Taken together, perturbation of the lung lipidome by 3R4F CS exposure was supported across multiple molecular layers and included differential effects on mono- and diacylated glycerophospholipids and induction of lung surfactant-related processes. In contrast, the CHTP 1.2 and THS 2.2 groups as well as the Cessation and Switch groups were associated with weaker to absent effects on the lung lipidome.

### 3.8 Discussion

Standard toxicological endpoints can lack sensitivity and only yield limited insights into toxicological mechanisms [7], [8]; therefore, systems toxicology approaches that complement apical measurements with high-resolution measurements using molecular profiling (omics) methods have been developed [9], [10]. To derive relevant toxicological insights from these data, robust computational analysis approaches are essential. For transcriptomic data, we have developed, and employed in this study, a causal biological network enrichment approach that quantitatively and statistically evaluates the perturbation of context-relevant causal network models [33], [34], [83], [127]. However, because toxicological effects encompass multiple molecular layers simultaneously, multi-omics measurements can support deeper insights into toxicological mechanisms and strengthen conclusions. For example, cigarette smoke (CS) exposure has detrimental effects on lung lipids [11], [12], metabolites [13], [14], proteins [15], and transcriptional programs [16], [17]. Several previous studies have demonstrated the benefit of such multi-omics approaches in toxicology studies, including the investigation of renal cisplatin toxicity [18], lung nanoparticle toxicity [19], and doxorubicin cardiotoxicity [20] and assessment of candidate MRTPs [21]. Moreover, recent review articles have emphasized the need for such integrative multi-omics analyses [9], [22].

To achieve such a multi-omics view within the current potential MRTP assessment study, we complemented standard lung endpoint measurements with five omics data layers for lung tissues: mRNA and miRNA transcriptomics, proteomics, metabolomics, and lipidomics. Individually, all five molecular layers supported the substantial effects of 3R4F CS exposure on the lungs of ApoE^−/−^ mice, whereas CHTP 1.2 and THS 2.2 aerosol exposure was associated with fewer to absent effects. However, the real strength of multi-omics approaches comes from data integration. To create such an integrative multi-omics view of the exposure effects, we leveraged the recently developed MOFA model [50] and, for biological interpretation, derived a functional association network for the main multi-omics exposure direction using the PCSF algorithm [55]. MOFA decomposes omics data matrices into one LF matrix and a weight matrix for each data modality. Similar to PCA for single-omics data, MOFA aims to identify an interpretable low-dimensional representation of the data, with LFs capturing the major sources of variation across the data modalities. For our data, the first LF clearly captured the main exposure effect across all five data modalities, further supporting a clear 3R4F CS effect on the lung. Conversely, CHTP 1.2 and THS 2.2 aerosol- and Sham-exposed samples remained adjacent in the multi-omics space, with intermediate (remaining) effects for the Cessation and Switch groups. Gene set enrichment analysis of the mRNA transcriptomics and proteomics data demonstrated that inflammatory processes had a major role in this effect. The biological network approach directly tied together the effects across the different molecular layers by embedding the molecules associated with the main exposure effect in the context of their known functional associations. In addition to inflammation, this analysis revealed xenobiotic/oxidative stress responses, lipid metabolism, and extracellular matrix as functional categories associated with the exposure response. Importantly, the molecular network directly highlighted molecular links across data modalities involved in these functional processes.

As mentioned, the identified multi-omics exposure response, which was driven predominantly by the 3R4F CS effect, was most clearly associated with lung immune response. Indeed, the scores of LF 1 correlated well with the total number of (immune) cells in BALF as a standard endpoint for the lung immune response. CS is known to trigger an extensive immune response in the lungs in both humans and rodents [16], [87], [88], [89], and chronic lung inflammation is an essential component of the pathomechanism of COPD [128], [129]. The current study further highlighted the highly interconnected immune response across the different molecular layers, representing both immune activating/effector mechanisms as well as an immunosuppressive/negative feedback mechanism (Table 2).

The metabolite itaconate and its enzyme Irg1 (Acod1) were strongly increased in abundance upon 3R4F CS exposure. Itaconate has been implicated as an immune-response regulator in macrophages [92], [93], [94], [95], but its role in lung immune response has not been characterized. Notably, a recently proposed anti-inflammatory mechanism suggested that itaconate acts via posttranslational modification of Keap1, leading to stabilization of the anti-oxidant and anti-inflammatory transcription factor Nrf2 (Nfe2l2) [92]. Thus, the increased abundance of the Irg1/itaconate module upon 3R4F CS exposure may not only prevent overshooting macrophage activation in the lungs but may also help counteract the oxidative challenge from the activated immune system as well as directly from CS exposure.

Polyamines (putrescine, N-acetyl-putrescine, and N-acetyl-spermidine) are other metabolites with potential immunoregulatory roles which were significantly increased upon 3R4F CS exposure. For example, polyamines have been implicated in the regulation of lymphocyte and macrophage activation [98], [99], [107]. Hardbower et al. [99] discovered that putrescine can temper the activation of proinflammatory M1 macrophages by controlling M1 transcriptional programs via histone modifications. Further, Fang et al. [107] found that ornithine decarboxylase (ODC1), the rate-limiting enzyme for polyamine synthesis, inhibits the inflammatory response and ROS-induced apoptosis in macrophages. In the context of lung diseases, Jain et al. [130] reviewed the possible roles of elevated polyamine levels found in asthma, which might involve activation of eosinophils, induction of oxidative bursts in neutrophils, induction of histamine release by mast cells, and M1 to M2 polarization in macrophages.

Quinolinate is a metabolite of the kynurenine pathway, by which tryptophan is metabolized to nicotinamide adenine dinucleotide. Several metabolites of this pathway, including quinolinate, are implicated in immunoregulatory functions [109], [110]. Of note, increased kynurenine levels have been observed in smokers [131], further supporting a likely functional role of these metabolites in CS exposure response.

miRNAs are important components of cellular response programs, including inflammatory responses in the lungs [132]. In the current study, 3R4F CS upregulated the expression of mmu-miR-146a, mmu-miR-21a, and mmu-miR-2137, all of which have been implicated in the regulation of immune responses. Mmu-miR-146a has been linked to the observed imbalance of Th1/Th2 lymphocytes in the lungs of mice exposed to airborne fine particulate matter [101]. mmu-miR-21a has been identified as a negative immune regulator, including in mouse liver regeneration (via NF-κB inhibition) [100], and an inhibitor of mmu-miR-2137 was found to increase TNFα secretion and decrease IL-10 secretion in macrophages in the context of *P. gingivalis* infection [106].

In addition to metabolites and miRNAs, and consistent with previous studies [12], [21], [133], [134], the multi-omics approach also associated lipid-related changes with the lung immune response upon 3R4F CS exposure. 3R4F CS exposure was associated with marked effects on the lung lipidome, encompassing a wide range of lipid classes, whereas CHTP1.2 and THS2.2 aerosol exposure had only very limited effects—with reversion of the 3R4F CS-induced effects upon cessation or switching to CHTP1.2. In addition, to their role in stabilization of alveoli, lipid–protein surfactant complexes are also essential components of the innate lung immune response [122]. Together with candidate surfactant lipids, only 3R4F CS exposure, not the MRTP aerosols, increased the abundance of Sftpa1 and Sftpd, the surfactant components involved in immune defense and surfactant homeostasis [123]. With this, consistent with our previous study [21], the current data support low to absent effects of the MRTP aerosols on lung lipid metabolism and pulmonary surfactant, which is in contrast to the recently reported observation that exposure to an e-cigarette aerosol –while not causing lung inflammation nor emphysema – altered lipid and surfactant-related processes in the lung of mice [135].

We also identified the increased abundance of phospholipase D3 (Pld3) upon 3R4F CS exposure as a component of the lipid metabolism cluster. Recently, an immune regulatory role was identified for Pld3, which can control Tlr9 activation of macrophages, likely via its 5′ exonuclease activity on nucleic acids [121].

Inflammatory and oxidative stress responses are tightly interconnected [12], [87], which is also exemplified by the abovementioned stabilizing effect of itaconate on the anti-oxidant and anti-inflammatory transcription factor Nrf2 (Nfe2l2) [92]. In the current study, the transcriptomics, proteomics, and metabolomics data all supported induction of oxidative stress response by 3R4F CS, whereas no signs of oxidative stress response were observed after CHTP 1.2 and THS 2.2 aerosol exposure, and both cessation and switching resulted in reversal of 3R4F CS-induced effects. This is consistent with the previously reported extensive cellular stress responses triggered by CS exposure in the lungs, and the lower to absent effects upon exposure to candidate MRTP aerosols [7], [14], [21], [90], [91], [114], [115], [116]. In particular, the multi-omics approach revealed induction of the heme–biliverdin–bilirubin pathway by 3R4F CS. This pathway counteracts oxidative stress both by producing antioxidants (biliverdin and bilirubin) and limiting the accumulation of the oxidant heme [117], [136], [137]. Especially interesting for our integrative network view of the effects of CS, engagement of this pathway has been linked with reduction in inflammatory markers in human subjects with Gilbert’s syndrome [138].

*Study strengths and limitations:* This study was based on a robustly generated systems toxicology data set for lung that comprises measurements across five molecular layers (mRNAs, miRNAs, proteins, lipids, and (other) metabolites). Beyond the analyses presented here, these data can support testing of novel multi-omics data analysis approaches and can be leveraged to gain additional insights into pulmonary responses to toxicant exposures. By combining latent factor identification (using the MOFA approach [50]) and multi-omics network analysis (supported by the Prize-Collecting Steiner Forest algorithm [55]), we demonstrate how multi-omics data can help to pinpoint specific molecular mechanisms – e.g., the engagement of specific immunoregulatory interactions and oxidative stress responses. These results can motivate further research into the complex molecular interactions during toxicological responses, as well as serve as a blueprint for the investigation of molecular mechanisms in other multi-omics studies. However, limiting the mechanistic conclusions that can be drawn from our study, the multi-omics measurements were conducted for bulk tissue rather than for individual cell types or single cells. Especially, single-cell mRNA sequencing is emerging rapidly as a powerful technique and already yielded important insights into lung cell types [139]. Lipidomics already has been applied to investigate lipid profiles across pulmonary cell types [140]. While it would be challenging to provide cell/cell type resolved data in the context of such a large-scale multi-omics study, having this information available would certainly support interpretation, especially of the observed immuno-regulatory processes. In addition, especially, interpretation of the metabolic changes would have benefited from a more dynamic view, e.g., using flux-based approaches rather than focusing on the steady-state conditions [141], [142].

## 4 Conclusions

This work exemplifies how multi-omics approaches can be leveraged within systems toxicology studies and the generated multi-omics data set can facilitate the development of analysis methods and can yield further insights into the effects of toxicological exposures on the lung of mice.

## 5 Authors’ contributions

B.T., J.S., A.S., B.P., T.S., O.L., E.T.W., M.T., N.V.I., P.V., F.M., M.V.P., and J.H. contributed to the conception or design of the work. B.P., C.N., T.S., S.D., O.L., A.E., E.G., E.T.W., and S.L. contributed to data collection. B.T., J.S., A.S., B.P., G.V., A.K., P.L., B.K., F.M., and J.H. contributed to data analysis and interpretation. B.T., S.G., S.C. contributed to data/code sharing. B.T., J.S., A.S., F.M., and J.H. drafted the article. All authors critically revised and approved the article.

## 6 Competing interests and funding

The work reported in this publication involved candidate/potential modified risk tobacco products developed by Philip Morris International. 10.13039/100014729Philip Morris International is the sole source of funding and sponsor of this research. Except B.K., all authors are employees of PMI R&D or had worked for PMI R&D under contractual agreements. B.K. is an employee of Metabolon Inc.
