# Supplementary material for: Multi-omics systems toxicology study of mouse lung assessing the effects of aerosols from two heat-not-burn tobacco products and cigarette smoke
Source: Comput Struct Biotechnol J. 2020 Apr 25;18:1056–73. doi: 10.1016/j.csbj.2020.04.011 (PMC7218232; doi:10.1016/j.csbj.2020.04.011)
Supplement: Supplementary data 1 [file mmc1.docx]

# *Supplementary Figures and Tables*

**Multi-omics systems toxicology study of mouse lung tissue assessing the biological effects of aerosols from two heat-not-burn tobacco products and cigarette smoke**

Bjoern Titz^1,*^, Justyna Szostak^1^, Alain Sewer^1^, Blaine Phillips^2^, Catherine Nury^1^, Thomas Schneider^1^, Sophie Dijon^1^, Oksana Lavrynenko^1^, Ashraf Elamin^1^, Emmanuel Guedj^1^, Ee Tsin Wong^2^, Stefan Lebrun^1^, Grégory Vuillaume^1^, Athanasios Kondylis^1^, Sylvain Gubian^1^, Stephane Cano^1^, Patrice Leroy^1^, Brian Keppler^3^, Nikolai V. Ivanov^1^, Patrick Vanscheeuwijck^1^, Florian Martin^1^, Manuel C. Peitsch^1^, and Julia Hoeng^1,*^

^1^ PMI R&D, Philip Morris Products S.A., Quai Jeanrenaud 5, CH-2000 Neuchâtel, Switzerland

^2^ Philip Morris International Research Laboratories Pte. Ltd., Science Park II, Singapore

^3^ Metabolon Inc., Research Triangle Park, NC, USA

**^#^ Corresponding authors:**

Julia Hoeng, Ph.D., MBA, PMI R&D, Philip Morris Products S.A., Quai Jeanrenaud 5, CH-2000 Neuchatel, Switzerland, julia.hoeng@pmi.com, Telephone: +41 (58) 242 2892

Bjoern Titz, Ph.D., PMI R&D, Philip Morris Products S.A., Quai Jeanrenaud 5, CH-2000 Neuchatel, Switzerland, bjorn.titz@pmi.com, Telephone: +41 (58) 242 2312

# Supplementary figures

**Supplementary figure 1. NPA heatmap.** NPA heatmap for the lungs. The heatmap shows NPAs for each network in the collection, across all conditions. Statistical significance is indicated (see key) where the NPA score is significant with respect to experimental variation (CI) and where two companion statistics (O and K) are significant (*p* values <0.05).

**Supplementary figure 2. Principal component score plots.** Score plots for the five data modalities and the first two components.


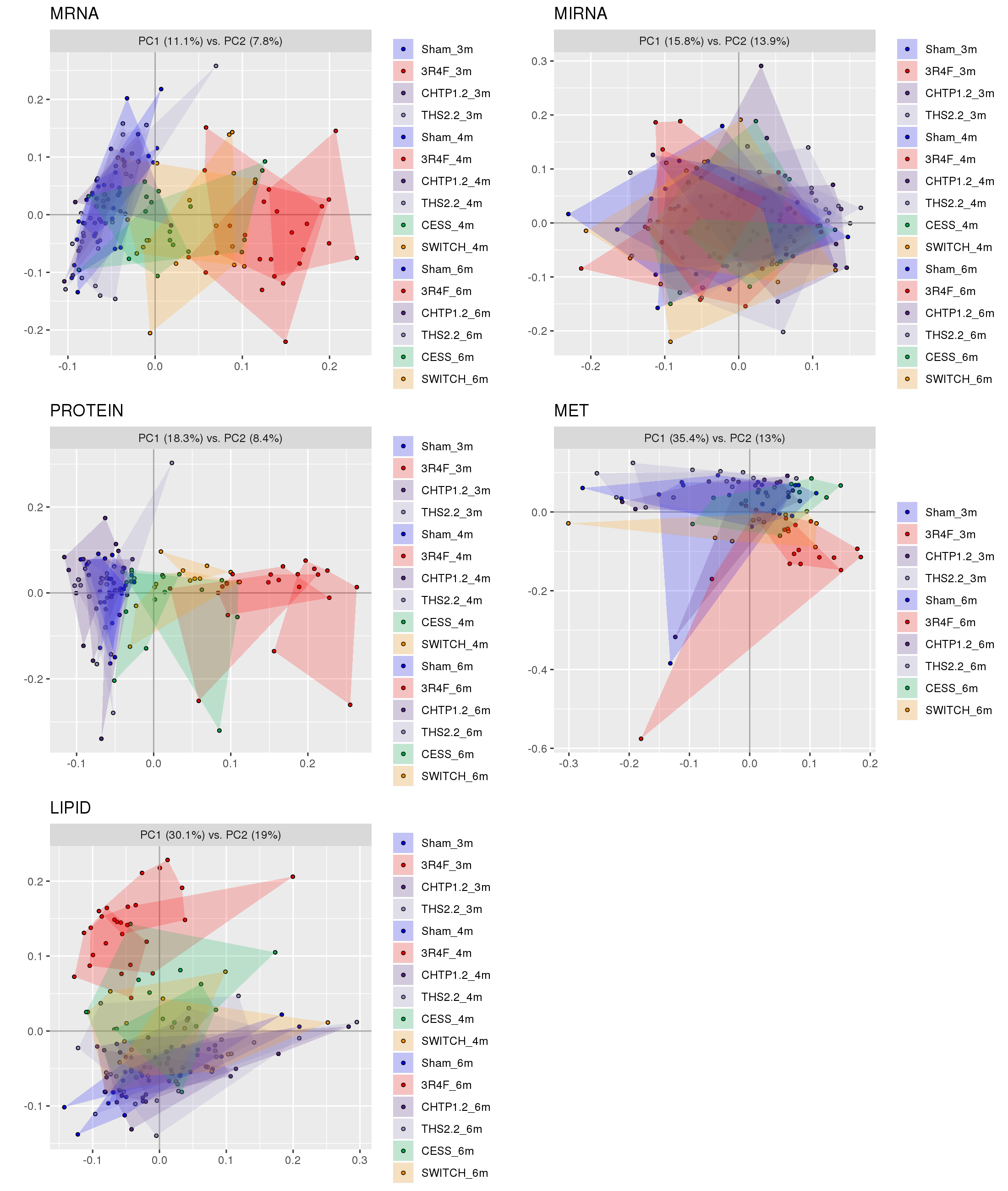


**Supplementary figure 3. Pair-wise MOFA score plots.** Score plots for latent factors (LF) 1–10. Each off-diagonal plot compares the sample scores for two latent factors, with the x-axis representing the LF corresponding to the plot column and the y-axis representing the LF corresponding to the plot row. The plots on the diagonal show the score distributions for each LF (colored by exposure type).

**Supplementary figure 4. sGCCA model. (A)** Fraction of explained variance for each data modality for the sGCCA model. **(B)** Score plot for components 1 and 2 for each data modality. **(C)** Score plots for component 1 only. **(D)** Rank comparison of the loading weights of component/LF 1 for the sGCCA and MOFA models across the five data modalities.


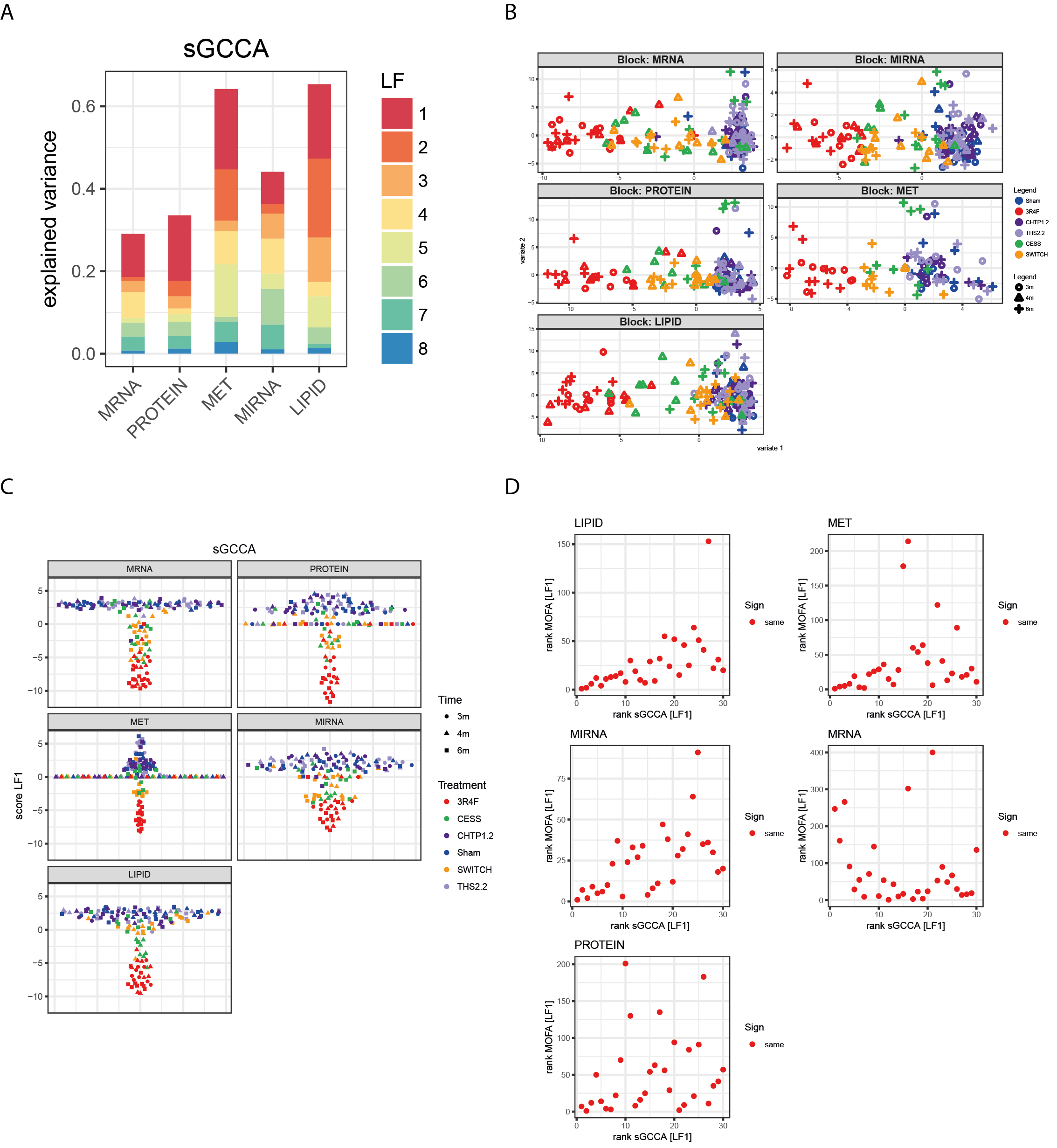


**Supplementary figure 5. Multi-omics response network (interactive).** [Unzip and open html file in browser].

**Supplementary figure 6. Lipid class heatmap.** Abundance profiles for lipid classes. Log_2_ fold changes versus Sham are color-coded, and statistical significance is indicated: FDR-adjusted *p* value <0.01; X, FDR-adjusted *p* value <0.05.

**Supplementary figure 7. Grid-search results for PCSF-based network identification.**


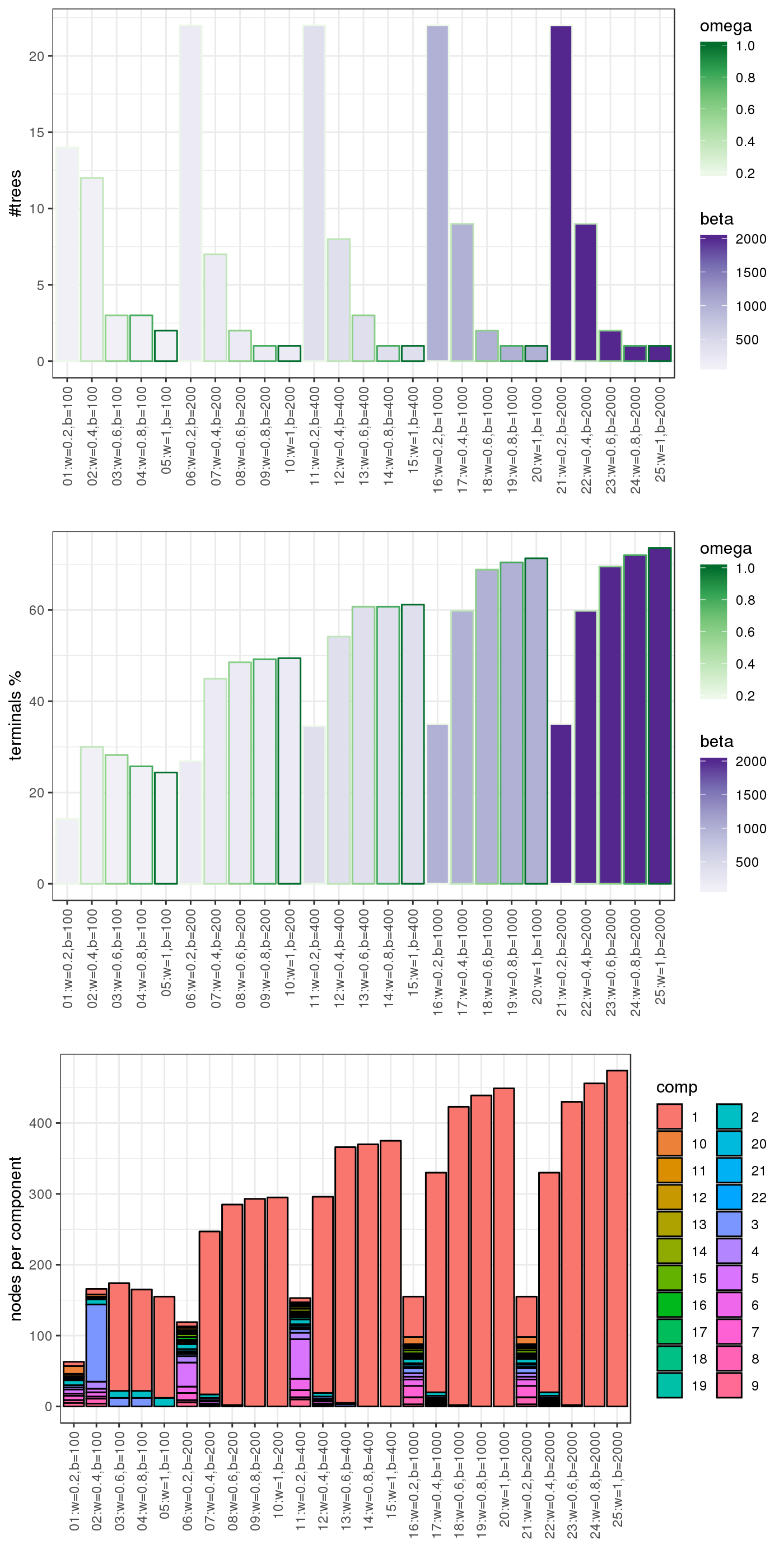


# Supplementary tables

**Supplementary table 1. Differentially abundant/expressed molecules for the five data modalities.**

*[See separate xlsx file]*

**Supplementary table 2. Identified network clusters.** Clusters, contained nodes, and enriched gene sets with FDR-adjusted *p* values.

| Cluster | Nodes | Gene sets |
| --- | --- | --- |
| 1 | AA467197;Abcd3;Acaa1a;Acaca;Acsl4;Hexadecanoic acid;HCO3-;Car4;Cav1;Dbi;Dlat;Fabp5;Fasn;Fdps;Gpnmb;Hpgd;Hsd17b4;Me1;Pdha1;Ppard;Ppt1;Ptk6;Ptrf;Scp2;Srebf2;Tpp1 | Fatty acid metabolism (1.85E-09)\|Metabolism of lipids (1.85E-09)\|Metabolism (3.09E-06)\|TYSND1 cleaves peroxisomal proteins (1.44E-04)\|Pyruvate metabolism (1.69E-04)\|Metabolism of steroids (2.43E-04)\|Fatty acyl-CoA biosynthesis (2.87E-04)\|Signaling by Retinoic Acid (4.65E-04)\|alpha-linolenic (omega3) and linoleic (omega6) acid metabolism (4.65E-04)\|alpha-linolenic acid (ALA) metabolism (4.65E-04)\|Activation of gene expression by SREBF (SREBP) (4.86E-04)\|Pyruvate metabolism and Citric Acid (TCA) cycle (7.22E-04)\|Regulation of pyruvate dehydrogenase (PDH) complex (7.22E-04)\|Regulation of cholesterol biosynthesis by SREBP (SREBF) (1.02E-03)\|Peroxisomal lipid metabolism (3.87E-03)\|PTK6 promotes HIF1A stabilization (1.06E-02)\|ChREBP activates metabolic gene expression (1.33E-02)\|Beta-oxidation of pristanoyl-CoA (1.61E-02)\|Beta-oxidation of very long chain fatty acids (2.33E-02)\|Peroxisomal protein import (3.00E-02)\|Signaling by Nuclear Receptors (4.09E-02)\|The citric acid (TCA) cycle and respiratory electron transport (4.17E-02) |
| 2 | Ablim3;Ager;Arhgdib;Cldn18;Cyba;Cybb;Dcn;Erbb2;Fgf1;Fgf23;Fgfr1;Galnt3;Gm6115;H1f0;Hcls1;Igf1;Map3k1;Muc1;Myo1f;Ncf1;Noxo1;Pdgfc;Rac1;Rac2;Sh3bp2;Sh3pxd2b;Slc11a1;Tjp1;Vav1 | RHO GTPases Activate NADPH Oxidases (4.89E-10)\|Signaling by Receptor Tyrosine Kinases (2.12E-07)\|Constitutive Signaling by Aberrant PI3K in Cancer (3.70E-04)\|Cross-presentation of particulate exogenous antigens (phagosomes) (3.70E-04)\|ROS, RNS production in phagocytes (4.21E-04)\|FGFR1c ligand binding and activation (6.25E-04)\|FGFR3 ligand binding and activation (6.25E-04)\|FGFR3c ligand binding and activation (6.25E-04)\|PI3K/AKT Signaling in Cancer (6.25E-04)\|PI5P, PP2A and IER3 Regulate PI3K/AKT Signaling (6.25E-04)\|Signaling by activated point mutants of FGFR1 (6.25E-04)\|VEGFA-VEGFR2 Pathway (6.25E-04)\|Signaling by Rho GTPases (6.38E-04)\|Negative regulation of the PI3K/AKT network (6.67E-04)\|Signaling by VEGF (6.67E-04)\|FGFR1 ligand binding and activation (7.31E-04)\|IGF1R signaling cascade (7.31E-04)\|IRS-related events triggered by IGF1R (7.31E-04)\|Phospholipase C-mediated cascade: FGFR1 (7.31E-04)\|Signaling by Type 1 Insulin-like Growth Factor 1 Receptor (IGF1R) (7.31E-04)\|PI-3K cascade:FGFR1 (1.56E-03)\|SHC-mediated cascade:FGFR1 (1.56E-03)\|FRS-mediated FGFR1 signaling (1.99E-03)\|RHO GTPase Effectors (3.47E-03)\|Downstream signaling of activated FGFR1 (4.40E-03)\|FGFR1 mutant receptor activation (4.40E-03)\|GPVI-mediated activation cascade (4.77E-03)\|Negative regulation of FGFR1 signaling (4.77E-03)\|Signaling by FGFR (4.77E-03)\|FGFR1b ligand binding and activation (5.54E-03)\|Detoxification of Reactive Oxygen Species (6.31E-03)\|Signaling by FGFR1 in disease (6.62E-03)\|Signaling by FGFR3 (7.50E-03)\|PI3K Cascade (7.84E-03)\|IRS-mediated signalling (1.01E-02)\|Signal Transduction (1.30E-02)\|Signaling by FGFR1 (1.30E-02)\|Insulin receptor signalling cascade (1.35E-02)\|CD28 dependent Vav1 pathway (1.77E-02)\|FGFR3 mutant receptor activation (1.77E-02)\|Signaling by activated point mutants of FGFR3 (1.77E-02)\|Rho GTPase cycle (1.81E-02)\|FGFR2c ligand binding and activation (1.94E-02)\|Phospholipase C-mediated cascade; FGFR3 (1.94E-02)\|DCC mediated attractive signaling (2.12E-02)\|FGFR4 ligand binding and activation (2.12E-02)\|Signaling by FGFR in disease (2.12E-02)\|PIP3 activates AKT signaling (2.22E-02)\|Phospholipase C-mediated cascade; FGFR4 (2.34E-02)\|MAPK family signaling cascades (2.67E-02)\|Activated point mutants of FGFR2 (2.90E-02)\|PI-3K cascade:FGFR3 (3.02E-02)\|Phospholipase C-mediated cascade; FGFR2 (3.02E-02)\|SHC-mediated cascade:FGFR3 (3.02E-02)\|Signaling by Insulin receptor (3.02E-02)\|Intracellular signaling by second messengers (3.08E-02)\|FGFR2 ligand binding and activation (3.43E-02)\|FRS-mediated FGFR3 signaling (3.43E-02)\|PI-3K cascade:FGFR4 (3.43E-02)\|SHC-mediated cascade:FGFR4 (3.43E-02)\|Signal transduction by L1 (3.72E-02)\|FRS-mediated FGFR4 signaling (3.84E-02)\|G alpha (12/13) signalling events (3.84E-02)\|Signaling by FGFR3 in disease (3.84E-02)\|Signaling by FGFR3 point mutants in cancer (3.84E-02)\|PI-3K cascade:FGFR2 (4.07E-02)\|SHC-mediated cascade:FGFR2 (4.07E-02)\|Innate Immune System (4.10E-02)\|Sema4D in semaphorin signaling (4.30E-02)\|Disease (4.38E-02)\|Downstream signaling of activated FGFR3 (4.48E-02)\|FRS-mediated FGFR2 signaling (4.48E-02)\|TRAF6 mediated NF-kB activation (4.77E-02)\|Antigen processing-Cross presentation (4.95E-02)\|Downstream signaling of activated FGFR4 (4.95E-02)\|VEGFR2 mediated vascular permeability (4.95E-02)\|Diseases of signal transduction (4.95E-02) |
| 3 | Ace2;Akr1b10;Anpep;L-Cysteine;Dihydrobiopterin;Tetrahydrobiopterin;D-Sorbitol;3-(4-Hydroxyphenyl)pyruvate;L-Cystathionine;Cbs;Fabp3;Gclc;Got1;Got2;Mme;Pah;Pank3;Pank4;Pcbd1;Ppcs;Qdpr;Tat;Tfpi;Vnn1;Vnn3 | Phenylalanine and tyrosine catabolism (3.82E-06)\|Vitamin B5 (pantothenate) metabolism (1.37E-05)\|Coenzyme A biosynthesis (1.47E-04)\|Metabolism (1.83E-04)\|Histidine, lysine, phenylalanine, tyrosine, proline and tryptophan catabolism (3.63E-04)\|Metabolism of amino acids and derivatives (5.18E-04)\|Metabolism of Angiotensinogen to Angiotensins (7.56E-04)\|Sulfur amino acid metabolism (2.50E-03)\|Metabolism of vitamins and cofactors (2.80E-03)\|Metabolism of water-soluble vitamins and cofactors (9.28E-03) |
| 4 | Acp5;Akr1a1;Aldh1a3;Aldh1b1;Aldh7a1;Aldh9a1;Asmt;Blvrb;D-Glucuronate;Riboflavin;Bilirubin;Estradiol-17beta;Imidazole-4-acetate;5-Hydroxyindoleacetate;Methylimidazoleacetic acid;Cyp1b1;Cyp2f2;Cyp4b1;Ephx1;Ugt1a10 | Biological oxidations (3.66E-06)\|Metabolism (2.44E-05)\|Phase I - Functionalization of compounds (5.66E-05)\|Cytochrome P450 - arranged by substrate type (2.44E-02)\|Fatty acids (4.79E-02) |
| 5 | Acss1;Acss2;Adh5;Aldh1a1;Aldh1a7;Aldh3a1;Aspa;Formate;N-Acetyl-L-aspartate;beta-Citryl-L-glutamate;Cyp2b13;Esd;Fmo3;Rimklb | Biological oxidations (1.02E-09)\|Phase I - Functionalization of compounds (1.02E-09)\|Ethanol oxidation (1.04E-07)\|Metabolism (3.08E-05) |
| 6 | Acta2;Actn3;Cnn1;Exoc4;Exoc7;Hopx;Itga8;Lcp1;Mb;mmu-miR-133a-3p;Myh10;Myh3;Myh6;Myl3;Myl4;Myo5a;Rilpl2;Runx2;S100a4;Spp1;Srf;Tagln;Tnfaip2;Tnnc1;Tnni3;Tnnt2;Tpm2;Zyx | Striated Muscle Contraction (7.13E-14)\|Muscle contraction (2.00E-08)\|Insulin processing (2.23E-02) |
| 7 | Adora2a;Cd79a;Cd83;Cd86;Csf2ra;Csf2rb;Csf2rb2;Fcgr2b;Gpr176;Igj;Inpp5d;Itgax;Pigr;Sfn;Sftpd;Tubb4a;Ywhag;Ywhaz | Defective CSF2RA causes pulmonary surfactant metabolism dysfunction 4 (SMDP4) (8.49E-05)\|Defective CSF2RB causes pulmonary surfactant metabolism dysfunction 5 (SMDP5) (8.49E-05)\|Surfactant metabolism (8.49E-05)\|Diseases associated with surfactant metabolism (1.36E-04)\|Interleukin-3, 5 and GM-CSF signaling (1.43E-04)\|Chk1/Chk2(Cds1) mediated inactivation of Cyclin B:Cdk1 complex (2.16E-04)\|Activation of BAD and translocation to mitochondria (2.94E-04)\|Interleukin receptor SHC signaling (1.63E-03)\|Activation of BH3-only proteins (2.01E-03)\|Diseases of metabolism (4.48E-03)\|Interleukin-2 family signaling (4.48E-03)\|Intrinsic Pathway for Apoptosis (4.48E-03)\|Signaling by Interleukins (4.48E-03)\|Translocation of GLUT4 to the plasma membrane (6.51E-03)\|RHO GTPases activate PKNs (1.15E-02)\|G2/M DNA damage checkpoint (2.03E-02)\|Cytokine Signaling in Immune system (2.72E-02)\|TP53 Regulates Metabolic Genes (2.72E-02) |
| 8 | AF251705;Cd200;Cd200r1;Cd200r4;Cd209a;Cd68;Clec4n;Clec5a;Emr1;Fcer1g;Fcgr3;Gnao1;Icam2;Itgam;Itgb2;Lgals3;Lsp1;Lyz2;Mrc1;Ms4a6d;Pik3r5;Rgs1;Rgs16;Trem2;Tyrobp | Immune System (2.54E-04)\|Immunoregulatory interactions between a Lymphoid and a non-Lymphoid cell (2.54E-04)\|Neutrophil degranulation (2.54E-04)\|Innate Immune System (5.83E-04)\|DAP12 interactions (4.12E-02) |
| 9 | Aicda;Thymidine;dCMP;Deoxyuridine;Deoxycytidine;Xanthosine;Deoxyinosine;Dck;Egr2;Ezh2;Hprt;Jarid2;Mafb;mmu-miR-155-5p;Pmaip1;Pnp;Rheb;Suz12;Tk2;Tymp | Nucleotide salvage (5.93E-08)\|Metabolism of nucleotides (7.00E-05)\|Pyrimidine salvage (1.28E-04)\|Purine salvage (1.66E-04)\|Activation of HOX genes during differentiation (9.04E-04)\|Activation of anterior HOX genes in hindbrain development during early embryogenesis (9.04E-04)\|PRC2 methylates histones and DNA (3.72E-03)\|Regulation of PTEN gene transcription (1.01E-02) |
| 10 | Aif1;Apoa1;Apob;C1qa;C1qb;C1qc;Calr;Cd36;Cd47;Cryab;Elane;Erp29;Ganab;Grpel1;Hp;Hsf1;Hspa1b;Hspa5;Lhfpl2;Manf;Marco;Mlec;Msr1;Olr1;P4hb;Pdia4;Prkcsh;Scgb1a1;Scgb3a2;Serpina3n;Sftpa1;Slpi;Thbs1 | Scavenging by Class A Receptors (2.24E-08)\|Binding and Uptake of Ligands by Scavenger Receptors (1.92E-06)\|Platelet degranulation (5.03E-05)\|Response to elevated platelet cytosolic Ca2+ (5.03E-05)\|Scavenging by Class B Receptors (1.77E-04)\|Innate Immune System (6.16E-04)\|Chylomicron assembly (7.51E-04)\|N-glycan trimming in the ER and Calnexin/Calreticulin cycle (7.53E-04)\|Neutrophil degranulation (1.70E-03)\|Platelet activation, signaling and aggregation (1.77E-03)\|Hemostasis (3.63E-03)\|Plasma lipoprotein assembly (3.63E-03)\|Immune System (3.67E-03)\|Calnexin/calreticulin cycle (7.87E-03)\|VLDL assembly (1.03E-02)\|HSF1-dependent transactivation (1.90E-02)\|Scavenging by Class F Receptors (1.90E-02)\|Cellular response to heat stress (2.18E-02)\|Post-translational protein phosphorylation (2.68E-02)\|ATF6 (ATF6-alpha) activates chaperone genes (3.29E-02)\|Chylomicron remodeling (3.29E-02)\|Regulation of Insulin-like Growth Factor (IGF) transport and uptake by Insulin-like Growth Factor Binding Proteins (IGFBPs) (4.05E-02)\|ATF6 (ATF6-alpha) activates chaperones (4.20E-02)\|Regulation of Complement cascade (4.20E-02) |
| 11 | Akr1b8;Aldoc;Bst1;NADP+;alpha-D-Ribose 1-phosphate;Quinolinate;G6pdx;Hk3;Nmnat1;Nmnat3;Pgd;Pgm1;Pygl;Qprt;Tkt | Pentose phosphate pathway (hexose monophosphate shunt) (2.02E-06)\|Metabolism (2.28E-06)\|Metabolism of carbohydrates (2.37E-06)\|Nicotinate metabolism (1.15E-05)\|Metabolism of vitamins and cofactors (3.79E-04)\|Metabolism of water-soluble vitamins and cofactors (2.10E-03)\|Neutrophil degranulation (2.49E-02)\|Glycogen breakdown (glycogenolysis) (2.90E-02) |
| 12 | Anxa1;Anxa2;Itaconate;C3;C3ar1;Ccl12;Ccl22;Ccl3;Ccl6;Ccl7;Ccl8;Ccl9;Ccr1;Cfb;Cxcl1;Cxcl10;Cxcl2;Cxcl5;Cxcl9;Hebp1;Irg1;Lgals1;P2ry6 | Peptide ligand-binding receptors (5.56E-10)\|Class A/1 (Rhodopsin-like receptors) (1.62E-09)\|GPCR ligand binding (4.62E-08)\|Chemokine receptors bind chemokines (8.68E-08)\|G alpha (i) signalling events (1.64E-07)\|Interleukin-10 signaling (6.48E-06)\|GPCR downstream signalling (3.31E-04)\|Signaling by GPCR (4.85E-04)\|Signaling by Interleukins (1.99E-03)\|Activation of C3 and C5 (8.36E-03)\|Formyl peptide receptors bind formyl peptides and many other ligands (1.82E-02)\|Cytokine Signaling in Immune system (2.06E-02) |
| 13 | Aoc1;Arg1;Arg2;Glycine;Putrescine;Tryptamine;N-Acetylputrescine;Cndp2;Gatm;Gm853;Inmt;Lap3;Retnla;Sat1 | Metabolism of polyamines (7.44E-04)\|Metabolism of amino acids and derivatives (4.38E-03)\|Urea cycle (1.59E-02) |
| 14 | Arf1;Asah1;Bcl2;Bcl2l1;Bid;Sphingomyelin;Ctsb;Ctsc;Ctsd;Ctsh;Ctss;Ctsz;Cyth4;Galc;Gba;Gla;Gns;H2-Aa;H2-Ab1;Hexb;Lamp1;Lamp2;Lgmn;mmu-miR-195a-5p;mmu-miR-503-5p;Mpeg1;Naga;Psap;Smpd1;Tpt1 | MHC class II antigen presentation (4.78E-09)\|Glycosphingolipid metabolism (1.10E-08)\|Neutrophil degranulation (1.35E-08)\|Innate Immune System (3.51E-07)\|Sphingolipid metabolism (5.73E-07)\|Immune System (2.55E-06)\|BH3-only proteins associate with and inactivate anti-apoptotic BCL-2 members (2.83E-04)\|Trafficking and processing of endosomal TLR (8.38E-04)\|Metabolism of lipids (9.16E-03)\|Lysosome Vesicle Biogenesis (1.48E-02)\|Adaptive Immune System (1.76E-02)\|Intrinsic Pathway for Apoptosis (2.46E-02) |
| 15 | Atox1;Atp7a;Cd177;F10;F7;Gapdh;Mcoln3;Prdx5;Procr;Rnase1;Rnh1;Rrm2;Sod2;Sod3;Thbd;Txn1 | Detoxification of Reactive Oxygen Species (1.39E-08)\|Formation of Fibrin Clot (Clotting Cascade) (1.69E-06)\|Common Pathway of Fibrin Clot Formation (1.31E-05)\|Cellular responses to stress (5.54E-03)\|Extrinsic Pathway of Fibrin Clot Formation (7.06E-03)\|Cellular responses to external stimuli (1.02E-02)\|Gamma-carboxylation of protein precursors (1.76E-02)\|Removal of aminoterminal propeptides from gamma-carboxylated proteins (1.76E-02)\|Transport of gamma-carboxylated protein precursors from the endoplasmic reticulum to the Golgi apparatus (1.76E-02)\|Gamma-carboxylation, transport, and amino-terminal cleavage of proteins (1.93E-02) |
| 16 | Atp5a1;Atp5c1;Atp5g1;Atp6v0d1;Atp6v0d2;Atp6v1a;Atp6v1e1;Atp6v1h;Basp1;Cox5a;Dhx9;Ehd2;Ehd4;Eif4a1;Eif4ebp1;Lamtor1;mmu-miR-21a-5p;mmu-miR-375-3p;Pdcd4;Ppa1;Rab11b;Rab11fip2;Rab5a;Scarb2;Smad7;Tcirg1;Trf;Yap1;Ybx1 | Transferrin endocytosis and recycling (1.11E-09)\|Insulin receptor recycling (2.52E-08)\|Iron uptake and transport (4.59E-08)\|ROS, RNS production in phagocytes (4.92E-08)\|Signaling by Insulin receptor (8.14E-06)\|Ion channel transport (1.19E-03)\|Formation of ATP by chemiosmotic coupling (2.71E-03)\|Cristae formation (1.28E-02)\|Signaling by Receptor Tyrosine Kinases (1.33E-02)\|Respiratory electron transport, ATP synthesis by chemiosmotic coupling, and heat production by uncoupling proteins. (4.00E-02)\|Transport of small molecules (4.00E-02) |
| 17 | Phosphatidylcholine;Phosphatidylglycerol;Phosphatidylethanolamine;Prostaglandin D2;Phosphatidylserine;1-Acyl-sn-glycero-3-phosphoethanolamine;Lcat;Lpcat1;Lpcat2b;Pisd;Pla2g2d;Pla2g7;Pld3;Pnpla7;Ptgds;Ptgs1;Tbxas1 | Metabolism of lipids (1.10E-04)\|Synthesis of Prostaglandins (PG) and Thromboxanes (TX) (3.04E-04)\|Phospholipid metabolism (6.60E-04)\|Glycerophospholipid biosynthesis (2.56E-03)\|Arachidonic acid metabolism (8.48E-03)\|Acyl chain remodelling of PG (4.10E-02)\|Metabolism (4.47E-02) |
| 18 | Cholesterol;beta-Sitosterol;Desmosterol;4-(N-Nitrosomethylamino)-1-(3-pyridyl)-1-butanone;4-(Methylnitrosamino)-1-(3-pyridyl)-1-butanol;Cbr2;Ch25h;Cyp7b1;Dhcr24;Hsd11b1;Hsd3b1;Hsd3b2;Hsd3b5;Lipa | Metabolism of steroids (5.74E-06)\|Glucocorticoid biosynthesis (5.30E-03)\|Metabolism of lipids (5.30E-03)\|Metabolism of steroid hormones (4.93E-02)\|Synthesis of bile acids and bile salts (4.93E-02) |
| 19 | Ccl2;Cd14;Csf2;Fhl1;Hdac5;Il1a;Il1r1;Il1rn;Il2;Irak1;Ly96;Malt1;mmu-miR-146a-5p;mmu-miR-2861;mmu-miR-34a-5p;Myd88;Notch1;Otub1;Rbpj;Rnf128;S100a8;S100a9;Sirt1;Stat5b;Tlr13;Tlr2;Tnfrsf9;Tollip;Traf6 | Toll-Like Receptors Cascades (8.15E-08)\|Regulation of TLR by endogenous ligand (1.96E-07)\|Immune System (1.11E-06)\|Interleukin-1 signaling (1.11E-06)\|Signaling by Interleukins (1.11E-06)\|MyD88 deficiency (TLR2/4) (2.44E-06)\|Cytokine Signaling in Immune system (2.89E-06)\|IRAK4 deficiency (TLR2/4) (2.89E-06)\|Toll Like Receptor 4 (TLR4) Cascade (2.89E-06)\|Interleukin-1 family signaling (4.53E-06)\|MyD88:Mal cascade initiated on plasma membrane (1.01E-05)\|Toll Like Receptor 2 (TLR2) Cascade (1.01E-05)\|Toll Like Receptor TLR1:TLR2 Cascade (1.01E-05)\|Toll Like Receptor TLR6:TLR2 Cascade (1.01E-05)\|Interleukin-10 signaling (1.08E-05)\|Diseases associated with the TLR signaling cascade (3.23E-05)\|Diseases of Immune System (3.23E-05)\|TRAF6 mediated IRF7 activation in TLR7/8 or 9 signaling (1.92E-04)\|TRAF6 mediated induction of NFkB and MAP kinases upon TLR7/8 or 9 activation (2.05E-04)\|MyD88 dependent cascade initiated on endosome (2.06E-04)\|Toll Like Receptor 7/8 (TLR7/8) Cascade (2.06E-04)\|MyD88-independent TLR4 cascade (2.30E-04)\|TRIF(TICAM1)-mediated TLR4 signaling (2.30E-04)\|Toll Like Receptor 3 (TLR3) Cascade (2.30E-04)\|Toll Like Receptor 9 (TLR9) Cascade (2.30E-04)\|p75NTR recruits signalling complexes (2.30E-04)\|IRAK2 mediated activation of TAK1 complex upon TLR7/8 or 9 stimulation (4.01E-04)\|TICAM1,TRAF6-dependent induction of TAK1 complex (4.01E-04)\|p75NTR signals via NF-kB (4.01E-04)\|TRAF6-mediated induction of TAK1 complex within TLR4 complex (4.70E-04)\|IKK complex recruitment mediated by RIP1 (1.17E-03)\|Innate Immune System (1.51E-03)\|ER-Phagosome pathway (2.42E-03)\|TAK1 activates NFkB by phosphorylation and activation of IKKs complex (2.43E-03)\|Metal sequestration by antimicrobial proteins (4.08E-03)\|Antigen processing-Cross presentation (4.35E-03)\|Ovarian tumor domain proteases (4.57E-03)\|Regulation of gene expression in late stage (branching morphogenesis) pancreatic bud precursor cells (5.26E-03)\|Interleukin-2 family signaling (6.58E-03)\|Interleukin-3, 5 and GM-CSF signaling (6.58E-03)\|NOTCH1 Intracellular Domain Regulates Transcription (7.82E-03)\|TRIF-mediated programmed cell death (8.13E-03)\|IRAK1 recruits IKK complex (9.69E-03)\|IRAK1 recruits IKK complex upon TLR7/8 or 9 stimulation (9.69E-03)\|Constitutive Signaling by NOTCH1 HD+PEST Domain Mutants (1.22E-02)\|Constitutive Signaling by NOTCH1 PEST Domain Mutants (1.22E-02)\|Signaling by NOTCH1 HD+PEST Domain Mutants in Cancer (1.22E-02)\|Signaling by NOTCH1 PEST Domain Mutants in Cancer (1.22E-02)\|Signaling by NOTCH1 in Cancer (1.22E-02)\|Interleukin-2 signaling (1.25E-02)\|NF-kB is activated and signals survival (1.39E-02)\|NICD traffics to nucleus (1.39E-02)\|Notch-HLH transcription pathway (1.39E-02)\|RUNX3 regulates NOTCH signaling (1.59E-02)\|Signaling by NOTCH1 (2.15E-02)\|Activation of IRF3/IRF7 mediated by TBK1/IKK epsilon (2.28E-02)\|JNK (c-Jun kinases) phosphorylation and activation mediated by activated human TAK1 (2.47E-02)\|Ligand-dependent caspase activation (2.47E-02)\|activated TAK1 mediates p38 MAPK activation (2.71E-02)\|Disease (3.00E-02)\|MyD88 cascade initiated on plasma membrane (3.02E-02)\|Toll Like Receptor 10 (TLR10) Cascade (3.02E-02)\|Toll Like Receptor 5 (TLR5) Cascade (3.02E-02)\|Antimicrobial peptides (3.96E-02)\|PI5P, PP2A and IER3 Regulate PI3K/AKT Signaling (4.02E-02)\|p75 NTR receptor-mediated signalling (4.08E-02)\|NOTCH3 Intracellular Domain Regulates Transcription (4.15E-02)\|TRAF6 mediated NF-kB activation (4.42E-02)\|Interleukin receptor SHC signaling (4.63E-02)\|Negative regulation of the PI3K/AKT network (4.63E-02)\|Caspase activation via extrinsic apoptotic signalling pathway (4.91E-02) |
| 20 | Col12a1;Col6a1;Col6a3;Cstb;Ctsk;Ctsl;Cxcl13;Igf2;Igfbp6;Itih4;Lcn2;Lgals3bp;Mmp12;Mmp13;Mmp9;Npy;Orm1;Orm2;Pf4;Saa3;Timp1 | Activation of Matrix Metalloproteinases (1.29E-06)\|Degradation of the extracellular matrix (1.29E-06)\|Extracellular matrix organization (1.29E-06)\|Platelet degranulation (1.29E-06)\|Response to elevated platelet cytosolic Ca2+ (1.29E-06)\|Collagen degradation (2.96E-06)\|Collagen formation (3.35E-06)\|Assembly of collagen fibrils and other multimeric structures (1.51E-05)\|Platelet activation, signaling and aggregation (6.86E-05)\|Interleukin-4 and 13 signaling (9.76E-03)\|Collagen chain trimerization (1.24E-02)\|RUNX1 regulates transcription of genes involved in differentiation of keratinocytes (1.44E-02)\|Hemostasis (2.93E-02)\|Collagen biosynthesis and modifying enzymes (3.20E-02)\|Trafficking and processing of endosomal TLR (3.20E-02)\|Peptide ligand-binding receptors (4.59E-02) |
| 21 | Fth1;Ftl1 |  |
