# Supplementary material for: Multi-omics systems toxicology study of mouse lung assessing the effects of aerosols from two heat-not-burn tobacco products and cigarette smoke
Source: Comput Struct Biotechnol J. 2020 Apr 25;18:1056–73. doi: 10.1016/j.csbj.2020.04.011 (PMC7218232; doi:10.1016/j.csbj.2020.04.011)
Supplement: Supplementary data 3 [file mmc3.pdf]

Multi-omics systems toxicology study of mouse lung tissue assessing the biological effects of aerosols from two heat-not-burn tobacco products and cigarette smoke

Bjoern Titz [1] Justyna Szostak [1] Alain Sewer [1] Blaine Phillips [2] Catherine Nury [1] Thomas Schneider [1] Sophie Dijon [1] Oksana Lavrynenko [1] Ashraf Elamin [1] Emmanuel Guedj [1] Ee Tsin Wong [2] Stefan Lebrun [1] Grégory Vuillaume [1] Athanasios Kondylis [1] Sylvain Gubian [1] Stephane Cano [1] Patrice Leroy [1] Brian Keppler [3] Nikolai V. Ivanov [1] Patrick Vanscheeuwijck [1] Florian Martin [1] Manuel C. Peitsch [1] Julia Hoeng [1]

[1] *PMI R&D, Philip Morris Products S.A., Quai Jeanrenaud 5, CH-2000 Neuchâtel, Switzerland*

[2] *Philip Morris International Research Laboratories Pte. Ltd., Science Park II, Singapore*

[3] *Metabolon Inc., Research Triangle Park, NC, USA*

17 September, 2019

Abstract

Cigarette smoke (CS) causes adverse health effects and, for smoker who do not quit, modified risk tobacco products (MRTPs) can be an alternative to reduce the risk of developing smoking-related diseases. Standard toxicological endpoints can lack sensitivity, with systems toxicology approaches yielding broader insights into toxicological mechanisms. In a 6-month systems toxicology study on ApoE-/- mice, we conducted an integrative multi-omics analysis to assess the effects of aerosols from the Carbon Heated Tobacco Product (CHTP) 1.2 and Tobacco Heating System (THS) 2.2—a potential and a candidate MRTP based on the heat-not-burn (HnB) principle- compared with CS at matched nicotine concentrations. Molecular exposure effects in the lungs were measured by mRNA/microRNA transcriptomics, proteomics, metabolomics, and lipidomics. Integrative data analysis included Multi-Omics Factor Analysis and multi-modality functional network interpretation. Across all five data modalities, CS exposure was associated with an increased inflammatory and oxidative stress response, and lipid/surfactant alterations. Upon HnB aerosol exposure these effects were much more limited or absent, with reversal of CS-induced effects upon cessation and switching to CHTP 1.2. Functional network analysis revealed CS-induced complex immunoregulatory interactions across the investigated molecular layers (e.g., itaconate, quinolinate, and miR-146) and highlighted the engagement of the heme–Hmox–bilirubin oxidative stress axis by CS. This work exemplifies how multi-omics approaches can be leveraged within systems toxicology studies and the generated multi-omics data set can facilitate the development of analysis methods and can yield further insights into the effects of toxicological exposures on the lung of mice.

Contents

1 License 2

2 Background information 3

3 Setup 3

4 Import the data 5

5 Overview of individual omes 5

6 Prepare multi-omics data 7

7 Exposure markers 11

8 mRNA-protein correlations 13

9 Biological impact factor (BIF) for lung 16

10 Principal component analysis (individual omes) 21

11 Multi-omics factor analysis 21
11.1 Generate model . . . 21
11.2 Explained variance, score, and loading plots . . . 23
11.3 GSEA for biological interpretation of LF1 . . . 28
11.4 LF1 vs inflammatory cells in BALF . . . 29

12 sGCCA: complement MOFA approach 32

13 MOFA vs sGCCA 33

14 Network-assisted interpretation of LF1 36
14.1 Create network . . . 36
14.2 PCSF-derived network . . . 39
14.3 GSEA for functional interpretation of clusters . . . 45
14.4 Response profiles . . . 48

15 Metabolite and miRNA changes associated with 3R4F CS-induced immune response 51
15.1 Network enrichment analysis for the Macrophage Signaling network . . . 51
15.2 Itaconate metabolic pathway & correlations . . . 52
15.3 Polyamine pathway . . . 54
15.4 Expression profiles for selected immune-related miRNAs . . . 58

16 Oxidative stress-related effects 58
16.1 Activation of hemoglobin–biliverdin–bilirubin pathway . . . 58
16.2 Oxidative stress-related metabolites, proteins, and network . . . 61

17 Effects on lipid metabolism 63
17.1 Lipid abundance for PEs and LPEs, glycerophospholipid metabolism, and surfactant . . . 63

18 Additional 80

19 Competing Interests statement 80

20 Version information 80

1 License

Copyright 2019 Philip Morris Products SA

This program is free software; you can redistribute it and/or modify it under the terms of the GNU General Public License as published by the Free Software Foundation; either version 2 of the License, or (at your option) any later version.

This program is distributed in the hope that it will be useful, but WITHOUT ANY WARRANTY; without even the implied warranty of MERCHANTABILITY or FITNESS FOR A PARTICULAR PURPOSE. See the GNU General Public License for more details.

You should have received a copy of the GNU General Public License along with this program; if not, write to the Free Software Foundation, Inc., 51 Franklin Street, Fifth Floor, Boston, MA 02110-1301, USA.

Also, see Licenses.txt

For the shared data:

This work is licensed under the Creative Commons Attribution 4.0 International License. To view a copy of this license, visit http://creativecommons.org/licenses/by/4.0/ or send a letter to Creative Commons, PO Box 1866, Mountain View, CA 94042, USA.

2 Background information

This Rmd file performs the analyses and creates the figures presented in the manuscript *Multi-omics systems toxicology of lung tissue reveals reduced biological effects of two heat-not-burn tobacco products compared with cigarette smoke*. In a six-month systems toxicology study with ApoE-/- mice, we assessed the effects of aerosols from two candidate modified-risk tobacco products, the Carbon Heated Tobacco Product (CHTP) 1.2 and the Tobacco Heating System (THS) 2.2, compared with cigarette smoke (CS) at matched nicotine concentrations. Molecular exposure effects in the lung were measured across five data modalities: mRNA/microRNA transcriptomics, proteomics, metabolomics, and lipidomics.

Analyses include:

- Single-omics analyses, e.g. differential expression analysis, principle component analysis, and causal network enrichment
- Multi-omics analysis by Multi-Omics Factor Analysis (Argelaguet et al., 2018, 10.15252/msb.20178124) and network-based interpretation

3 Setup

This section defines the basic processing parameters and loads the required R packages.

```
# general options
X11.options(type = "Xlib", bg = "white")
options(stringsAsFactors = FALSE)
Sys.setenv(R_MAX_NUM_DLLS = 500)

# load R packages
library(knitr)
library(gridExtra)
library(RColorBrewer)
library(ggplot2)
library(egg)
library(ggpubr)
library(reshape2)
library(xlsx)
library(readxl)
library(openxlsx)
library(tools)
library(plotrix)
library(gdata)
library(plyr)
library(dplyr)
library(limma)
library(NPA)
library(MOFA)
library(mixOmics)
library(ggbeeswarm)
library(stringi)
library(visNetwork)
library(PCSF)

#source all functions
for (fn in list.files("../SCRIPTS/FUNCTIONS", "*.R",full.names = TRUE)) {
```

```r
    source(fn)
}

# study definitions
script.name <- "P15038_APOE_P2_MultiOmicsManuscript.Rmd"
study_short_name <- "APOE_P2"
tissue <- "Lung"
subset <- "Tissue"
studynumber <- "P15038"
species <- "mouse"
species0 <- as.character(c(rat = "Rn", mouse = "Ms", human = "Hs")[species])
studyName <- studyname <- paste(studynumber, study_short_name, tissue, subset, sep = "_")

# layout parameters
volcano_nrow <- 5
volcano_ncol <- 2
which_order_volcano <- c(1:6, -1, 7, -1, 8)
layout_mat_volcano <- getLayoutMatrix(which_order_volcano, norow = volcano_nrow, nocol = volcano_ncol)

colgrps <- matrix(c(
  "Sham", "Sham", "#0000FF",
  "3R4F", "3R4F", "#FF0000",
  "CHTP", "CHTP1.2", "#542788",
  "THS", "THS2.2", "#998EC3",
  "Cess", "CESS", "#00B050",
  "Switch_CHTP", "SWITCH", "#FF9900"
), byrow = TRUE, ncol = 3, dimnames = list(NULL, c("Group_Name", "Label", "Color")))

# folders & files
projectBaseDir <- "../"
RobjectsDir <- file.path(projectBaseDir, "DATA")
reportDir <- file.path(projectBaseDir, "REPORT")
#dir.create(reportDir)
#setwd(projectBaseDir)

debug <- FALSE

# Rerun the analyses or load from file (if available)?
force_rerun_mofa <- TRUE
force_rerun_pcsf <- TRUE # requires integrative network & KEGG license
force_recreate_network <- FALSE # KEGG license required

# rendering options
opts_chunk$set(dev = 'pdf',
               fig.width=6,
               fig.height=5,
               message=FALSE,
               warning=FALSE)

#https://github.com/jhollist/manuscriptPackage
# Table Captions from @DeanK on http://stackoverflow.com/questions/15258233/
#  using-table-caption-on-r-markdown-file-using-knitr-to-use-in-pandoc-to-convert-t
#Figure captions are handled by LaTeX
knit_hooks$set(tab.cap = function(before, options, envir) {
                  if(!before) {
                    paste('\n\n:', options$tab.cap, sep='')
                  }
                })
default_output_hook = knit_hooks$get("output")
knit_hooks$set(output = function(x, options) {
  if (!is.null(options$tab.cap)) {
    x
  } else
    default_output_hook(x,options)
})
#https://stackoverflow.com/questions/25646333/code-chunk-font-size-in-rmarkdown-with-knitr-and-latex
def.chunk.hook  <- knitr::knit_hooks$get("chunk")
knitr::knit_hooks$set(chunk = function(x, options) {
  x <- def.chunk.hook(x, options)
  ifelse(options$size != "normalsize", paste0("\\", options$size,"\n\n", x, "\n\n \\normalsize"), x)
})

set.seed(456463)
```

4 Import the data

This section imports the data objects for the five data modalities. The R data objects are located in the ..//DATA folder:

- DATA objects: Quantification results for each omics data modality. List with the following slots:
  - data: the data matrix (analytes x samples)
  - I: metadata (sample x covariate)
  - L: Group annotation
  - CO: contrast matrix
- IDMAP object: Differential expression results. Lists with one named element per contrast. Each element is a data.frame, which includes the following columns:
  - nodeLabel: Name of the analyte (e.g., gene symbol)
  - foldChange: log2 fold-change
  - p.value: p-value
  - adj.p.value: BH-adjusted p-value

```
# load idmaps
idmaps <- list()
idmaps[["MRNA"]] <- load2(file.path(RobjectsDir, "IDMAP_P15038-S185500_APOE_P2_Lung_Mm_MRNA.rda"))
idmaps[["METABOLITE"]] <- load2(file.path(RobjectsDir, "IDMAP-Tissue_APOE_P2_Lung_Ms_METABOLOMICS.rda"))
idmaps[["PROTEIN"]] <- load2(file.path(RobjectsDir, "IDMAP-_APOE_P2_Lung_Mm_PEX.rda"))
idmaps[["MIRNA"]] <- load2(file.path(RobjectsDir, "IDMAP_P15038-S185500_APOE_P2_Lung_Mm_MIRNA.rda"))
idmaps[["LIPID"]] <- load2(file.path(RobjectsDir, "IDMAP-P15038-S186400_APOE_P2_Lung_Mm_LIPSG.rda"))

# contrast names (shorten, as all are vs sham)
idmaps <- lapply(idmaps, function(idmap) {
  names(idmap) <- gsub(" vs.+", "", gsub("_", " ", names(idmap)), perl = TRUE)
  return(idmap)
})

# load dat objects
dats <- list()
dats[["MRNA"]] <- load2(file.path(RobjectsDir, "DATA_P15038-S185500_APOE_P2_Lung_Mm_MRNA.rda"))
dats[["METABOLITE"]] <- load2(file.path(RobjectsDir, "DATA-Tissue_APOE_P2_Lung_Ms_METABOLOMICS.rda"))
dats[["PROTEIN"]] <- load2(file.path(RobjectsDir, "DATA-_APOE_P2_Lung_Mm_PEX.rda"))
dats[["LIPID"]] <- load2(file.path(RobjectsDir, "DATA-P15038-S186400_APOE_P2_Lung_Mm_LIPSG.rda"))

dats[["MIRNA"]] <- load2(file.path(RobjectsDir, "DATA_P15038-S185500_APOE_P2_Lung_Mm_MIRNA.rda"))
rownames(dats[["MIRNA"]]$data) <- as.character(dats[["MIRNA"]]$R[match(rownames(dats[["MIRNA"]]$data),
                            as.character(dats[["MIRNA"]]$R$Probe.Set.Name)), "Transcript.ID.Array.Design."])
```

5 Overview of individual omes

```
# number of 'captured' molecules
res <- lapply(dats, function(x) {
  X <- x$data
  X <- X[!apply(X, 1, function(y) all(is.na(y))), ]
  nrow(X)
})
tab_captured_molecules <- as.data.frame(unlist(res))
colnames(tab_captured_molecules) <- c("Number of molecules")

# Number of DEGs plot
dat <- do.call(rbind, lapply(names(idmaps), function(x) {
  no_degs <- sapply(getDEG(idmaps[[x]]), nrow)
  data.frame(type = x, contrast = names(no_degs), no_degs = as.numeric(no_degs))
}))
dat$contrast <- factor(dat$contrast, levels = names(idmaps[["MRNA"]]))
dat$treatment <- getsplit(dat$contrast, " ", 1)
dat$type <- factor(paste0(dat$type, "s"), levels = c("MRNAs", "PROTEINs", "METABOLITEs", "MIRNAs", "LIPIDs"))
tab_de <- dat

cols <- attr(idmaps[["MRNA"]], "colors")
names(cols) <- names(idmaps[["MRNA"]])

p <- ggplot(aes(x = contrast, y = no_degs), data = dat)
p <- p + geom_bar(aes(fill = contrast), stat = "identity")
p <- p + scale_fill_manual("", values = cols, guide = FALSE)
```

```
p <- p + facet_wrap(~type, ncol = 2, scales = "free_y")
p <- p + theme_bw()
p <- p + theme(axis.text.x = element_text(angle = 90, vjust = 0.5, hjust = 1))
p <- p + labs(x = "", y = "# differentially abundant/expressed")
p <- p + theme(strip.background = element_blank())
p <- p + theme(strip.text.x = element_text(size = 12, face = "bold"))

P_nodegs <- p

pdf(file = file.path(reportDir, "P15038_LungManuscript_NoDEGs_w_lipids.pdf"), width = 7, height = 10)
print(p)
dev.off()
```

```
## pdf
##   2
```

```
# Exports DEGs for Supplement
degs_list <- lapply(idmaps, function(idmap) {
  degs <- getDEG(idmap)
  do.call(rbind,lapply(names(degs), function(nm) {
      x <- as.data.frame(degs[[nm]])
      x <- x[,c("nodeLabel", "foldChange", "t", "df", "p.value", "adj.p.value"), drop = FALSE]
      if (nrow(x) > 0) {
        x$contrast <- nm
      }
      return(x)
  }))
})
file <- file.path(reportDir, "P15038_LungManuscript_DiffExpression.xlsx")
openxlsx::write.xlsx(degs_list, file = file)
```

Table 1: Total number of captured molecules.

	Number of molecules
MRNA	17473
METABOLITE	674
PROTEIN	5002
LIPID	399
MIRNA	363

Table 2: Total number of captured molecules.

type	contrast	no_degs	treatment
MRNAs	3R4F 3m	2313	3R4F
MRNAs	3R4F 4m	2649	3R4F
MRNAs	3R4F 6m	4324	3R4F
MRNAs	CHTP1.2 3m	0	CHTP1.2
MRNAs	CHTP1.2 4m	0	CHTP1.2
MRNAs	CHTP1.2 6m	0	CHTP1.2
MRNAs	THS2.2 3m	0	THS2.2
MRNAs	THS2.2 4m	0	THS2.2
MRNAs	THS2.2 6m	1	THS2.2
MRNAs	CESS 4m	604	CESS
MRNAs	CESS 6m	0	CESS
MRNAs	SWITCH 4m	66	SWITCH
MRNAs	SWITCH 6m	1191	SWITCH
METABOLITEs	3R4F 3m	173	3R4F
METABOLITEs	3R4F 6m	119	3R4F
METABOLITEs	CHTP1.2 3m	0	CHTP1.2
METABOLITEs	CHTP1.2 6m	0	CHTP1.2
METABOLITEs	THS2.2 3m	0	THS2.2
METABOLITEs	THS2.2 6m	0	THS2.2

type	contrast	no_degs	treatment
METABOLITEs	CESS 6m	0	CESS
METABOLITEs	SWITCH 6m	15	SWITCH
PROTEINs	3R4F 3m	271	3R4F
PROTEINs	3R4F 4m	266	3R4F
PROTEINs	3R4F 6m	529	3R4F
PROTEINs	CHTP1.2 3m	0	CHTP1.2
PROTEINs	CHTP1.2 4m	0	CHTP1.2
PROTEINs	CHTP1.2 6m	0	CHTP1.2
PROTEINs	THS2.2 3m	0	THS2.2
PROTEINs	THS2.2 4m	0	THS2.2
PROTEINs	THS2.2 6m	0	THS2.2
PROTEINs	CESS 4m	60	CESS
PROTEINs	CESS 6m	0	CESS
PROTEINs	SWITCH 4m	1	SWITCH
PROTEINs	SWITCH 6m	101	SWITCH
MIRNAs	3R4F 3m	21	3R4F
MIRNAs	3R4F 4m	16	3R4F
MIRNAs	3R4F 6m	43	3R4F
MIRNAs	CHTP1.2 3m	0	CHTP1.2
MIRNAs	CHTP1.2 4m	0	CHTP1.2
MIRNAs	CHTP1.2 6m	0	CHTP1.2
MIRNAs	THS2.2 3m	0	THS2.2
MIRNAs	THS2.2 4m	0	THS2.2
MIRNAs	THS2.2 6m	0	THS2.2
MIRNAs	CESS 4m	1	CESS
MIRNAs	CESS 6m	0	CESS
MIRNAs	SWITCH 4m	1	SWITCH
MIRNAs	SWITCH 6m	1	SWITCH
LIPIDs	3R4F 3m	100	3R4F
LIPIDs	3R4F 4m	120	3R4F
LIPIDs	3R4F 6m	122	3R4F
LIPIDs	CHTP1.2 3m	0	CHTP1.2
LIPIDs	CHTP1.2 4m	0	CHTP1.2
LIPIDs	CHTP1.2 6m	0	CHTP1.2
LIPIDs	THS2.2 3m	0	THS2.2
LIPIDs	THS2.2 4m	0	THS2.2
LIPIDs	THS2.2 6m	0	THS2.2
LIPIDs	CESS 4m	8	CESS
LIPIDs	CESS 6m	0	CESS
LIPIDs	SWITCH 4m	0	SWITCH
LIPIDs	SWITCH 6m	1	SWITCH

Table 1 lists the total number of captured molecules across the five data modalities. Figure 1 shows the number of differentially expressed molecules for each omics data modality. Table 2 lists the number of differentially expressed molecules for each omics data modality.

6 Prepare multi-omics data

In this section, we prepare the multi-omics data set for further analysis. This includes:

- Aligning the five data matrices by unique animal number (CAN) across columns

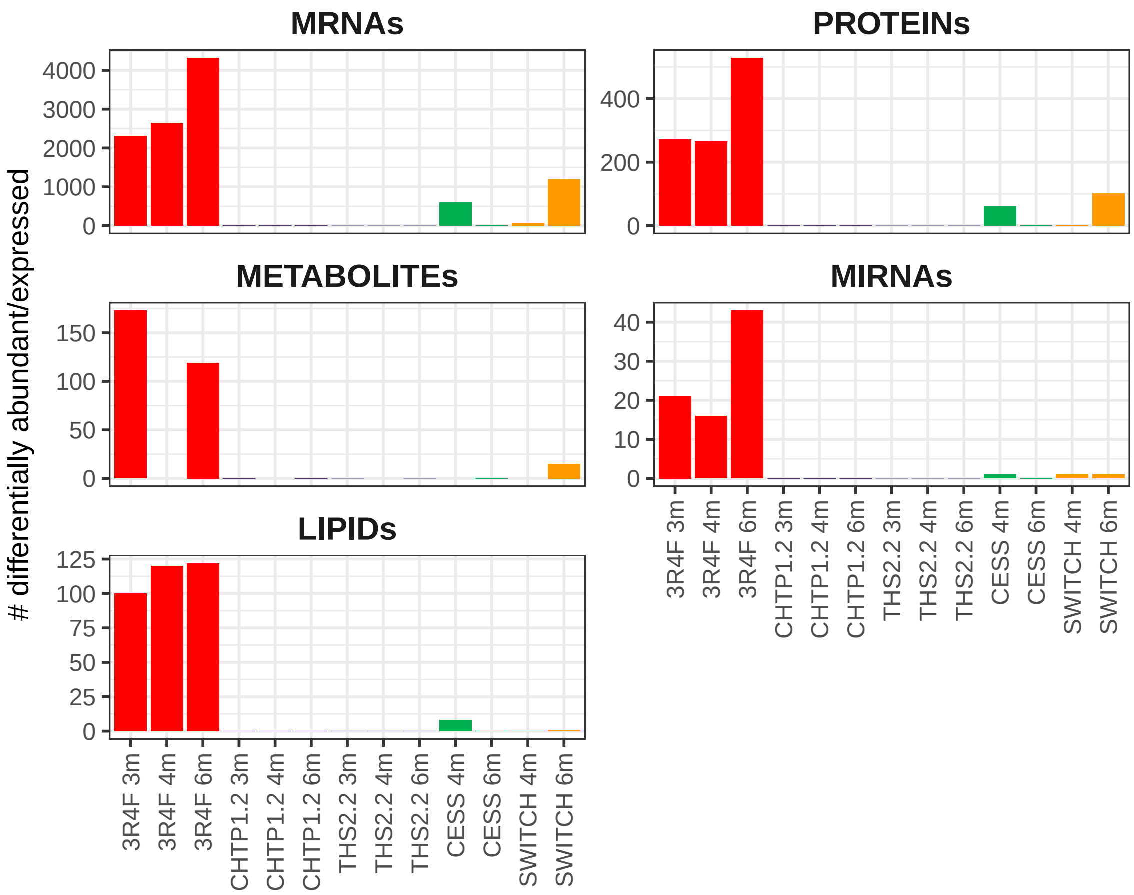

Figure 1: Number of differentially expressed molecules across omics data modalities.

- Summarizing available data types per animal
- Allowing for two missing data modalities for each animal
- Proteomics data has missing values: filter for proteins with missing values for less than 10% of samples
- Create multi-omics data list with associated meta data

```
# mRNA data
Xmrna <- dats[["MRNA"]]$data
colnames(Xmrna) <- as.character(dats[["MRNA"]]$I$CAN.PatientID)

# miRNA data
Xmirna <- dats[["MIRNA"]]$data
colnames(Xmirna) <- as.character(dats[["MIRNA"]]$I$CAN.PatientID)

# iTRAQ/proteomics data
Xprot <- dats[["PROTEIN"]]$data
colnames(Xprot) <- as.character(dats[["PROTEIN"]]$I$Subject.number)

# get CAN
# original SDMS link not used here to facilitate external reproducibility
filename <- file.path("../", "INFO", "S186500_NA_LUNG-L_ITRAQ_NA_15038ProteomicsSliceTissue-SampleMetaData.xlsx")
metadat <- readxl::read_xlsx(filename, sheet = 4) %>% as.data.frame()
colnames(Xprot) <- metadat[match(colnames(Xprot), metadat$`Child Sample ID`), "CAN/PatientID"]
# filter for proteins with less than 10% missing values
perc_not_na <- apply(Xprot, 1, function(x) sum(!is.na(x)) / length(x))
Xprot <- Xprot[perc_not_na > 0.90, ]

# metabolomics data
Xmet <- dats[["METABOLITE"]]$data
colnames(Xmet) <- gsub("S", "", as.character(dats[["METABOLITE"]]$I$ANIMAL_ID))

# lipidomics data
Xlip <- dats[["LIPID"]]$data
I <- dats[["LIPID"]]$I

# get CAN
# original SDMS link not used here to facilitate external reproducibility
filename <- file.path("../","INFO", "S186400_NA_LUNG-L_NA_NA_15038LipidomicsSliceTissue-SampleMetaData.xlsx")
metadat <- readxl::read_xlsx(filename, sheet = 4) %>% as.data.frame()
colnames(Xlip) <- metadat[match(I[, "SAMPLE_ID_1"], metadat$`Child Sample ID`), "CAN/PatientID"]

# get meta data for all samples
# original SDMS link not used here to facilitate external reproducibility
filename <- file.path("../","INFO", "15038_NA_NA_NA_NA_SelectedSampleListSysTox.xlsx")
metadat_all <- readxl::read_xlsx(filename, sheet = 1) %>% as.data.frame()
metadat_all$TreatmentGroupName <- gsub("THS", "THS2.2",
                                       gsub("Switch_CHTP", "SWITCH",
                                            gsub("Cess", "CESS",
                                                 gsub("^CHTP", "CHTP1.2", metadat_all$TreatmentGroupName))))
metadat_all$TreatmentGroupName <- factor(metadat_all$TreatmentGroupName,
                                         levels = unique(metadat_all$TreatmentGroupName))

# summary matrix of available samples
dat <- rbind(
  data.frame(type = "MRNA", CAN = colnames(Xmrna), value = TRUE),
  data.frame(type = "MIRNA", CAN = colnames(Xmirna), value = TRUE),
  data.frame(type = "PROTEIN", CAN = colnames(Xprot), value = TRUE),
  data.frame(type = "METABOLITE", CAN = colnames(Xmet), value = TRUE),
  data.frame(type = "LIPID", CAN = colnames(Xlip), value = TRUE)
)
mat <- reshape2::dcast(dat, type ~ CAN, value.var = "value")
rownames(mat) <- mat$type
mat <- mat[, -1]
mat[is.na(mat)] <- 0
mat[mat == TRUE] <- 1

#Updated to improve readability of sample plot
#hc <- hclust(dist(t(mat), method = "manhattan"), method = "average")
#dat$CAN <- factor(dat$CAN, levels = hc$labels[hc$order])
dat$Group <- metadat_all[match(dat$CAN, metadat_all$`CAN/PatientID`), "TreatmentGroupName"]
dat$CAN <- with(dat,factor(CAN, levels = unique(CAN[order(Group, CAN)])))

dat$TTT <- getsplit(dat$Group, "_", 1)
dat$Time <- getsplit(dat$Group, "_", 2)

cols <- colgrps[,"Color"]
names(cols) <- colgrps[,"Label"]

p <- ggplot(aes(x = CAN, y = type, fill = TTT), data = dat)
p <- p + geom_tile(na.rm = TRUE)
p <- p + scale_fill_manual("", values = cols,guide = FALSE, na.value = "grey20")
```

```
p <- p + facet_grid(~Time, scales = "free_x", space = "free_x")
p <- p + labs(x = "Lung samples", y = "")
p <- p + theme_bw()
p <- p + theme(axis.text.x = element_blank())
p <- p + theme(axis.text.y = element_text(color = "black"))
p <- p + theme(axis.ticks = element_blank())
p <- p + theme(plot.background = element_blank())
p_shared_samples_hm <- p

pdf(file = file.path(reportDir, "P15038_LungManuscript_SharedSamples.pdf"), width = 7, height = 2.5)
    plot(p_shared_samples_hm)
dev.off()
```

```
## pdf
##   2
```

```
tmp <- as.matrix(table(apply(mat, 2, sum, na.rm = TRUE)))
tmp2 <- as.numeric(tmp)
names(tmp2) <- rownames(tmp)
tmp <- cumsum(rev(tmp2))
tmp
```

```
##   5   4   3
##  69 129 144
```

```
dat2 <- data.frame(datasets = names(tmp), samples = tmp)
p <- ggplot(aes(x = datasets, y = samples), data = dat2)
p <- p + geom_bar(stat = "identity", colour = "black", fill = "grey30", width = 0.6)
p <- p + theme_bw()
p <- p + labs(x = "# data sets", y = "# samples")
p_shared_samples_cumsum <- p

pdf(file = file.path(reportDir, "P15038_LungManuscript_SharedSamplesCumSum.pdf"), width = 3, height = 3)
plot(p_shared_samples_cumsum)
dev.off()
```

```
## pdf
##   2
```

```
# Number of data types per animal
tab <- table(c(colnames(Xmrna), colnames(Xmirna), colnames(Xprot), colnames(Xmet), colnames(Xlip)))

# allow for two missing data types
common_samples <- names(tab)[tab >= 3]

data_list <- list(
  MRNA = Xmrna[, match(common_samples, colnames(Xmrna))],
  MIRNA = Xmirna[, match(common_samples, colnames(Xmirna))],
  PROTEIN = Xprot[, match(common_samples, colnames(Xprot))],
  MET = Xmet[, match(common_samples, colnames(Xmet))],
  LIPID = Xlip[, match(common_samples, colnames(Xlip))]
)
data_list <- lapply(data_list, function(x) {
  colnames(x) <- common_samples
  return(x)
})

tab_data_dimensions <- as.data.frame(vapply(data_list, nrow, numeric(1)))
colnames(tab_data_dimensions) <- c("Number of molecules")

# get meta data for all samples
# original SDMS link not used here to facilitate external reproducibility
filename <- file.path("../","INFO", "15038_NA_NA_NA_NA_SelectedSampleListSysTox.xlsx")
metadat <- readxl::read_xlsx(filename, sheet = 1) %>% as.data.frame()

dat_meta <- data.frame(common_samples, metadat_all[match(common_samples, metadat_all$`CAN/PatientID`), "TreatmentGroupName"])
colnames(dat_meta) <- c("CAN", "Group")

save(data_list, dat_meta, file = file.path(reportDir, "P15038_LungManuscript_data_list.rda"))
# load(file.path(reportDir, "P15038_LungManuscript_data_list.rda"))
```

Table 3: Data dimensions. Number of molecules considered for multi-omics analysis.

	Number of molecules
MRNA	17473
MIRNA	363
PROTEIN	1395
MET	674

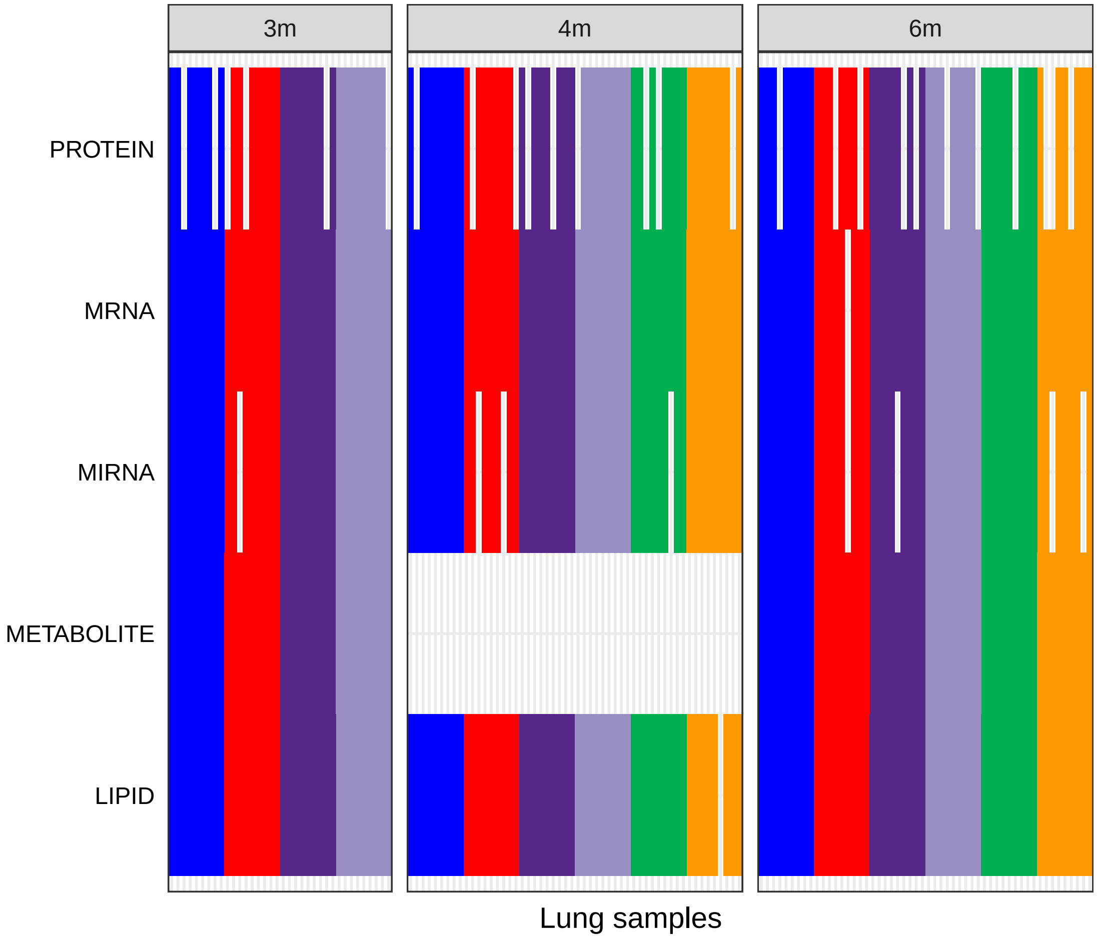

Figure 2: Lung samples obtained and analyzed from the same animals across the five omics analyses. A colored cell in the matrix indicates that a lung sample from an animal was analyzed successfully for the given omics data set. Note that only samples from the 3- and 6-month time point were submitted for metabolomics analysis.

	Number of molecules
LIPID	399

Figure 2 shows the data modalities available for animal, with the number of animals with >= 3, 4, or 5 available data modalities summarized in Figure 3. Table 3 lists the number of considered molecules across the five data modalities.

7 Exposure markers

```
# get current non-omics data compilation
file <- file.path("../","DATA", "P15038_APOE_P2_Exposure.rda")
dat_comb <- load2(file)
```

```
## Loading objects:
##   dat_comb
```

```
dat_comb$Group <- as.character(dat_comb$Group)
dat_comb <- dat_comb[grep("[56]m", dat_comb$Group), ]
```

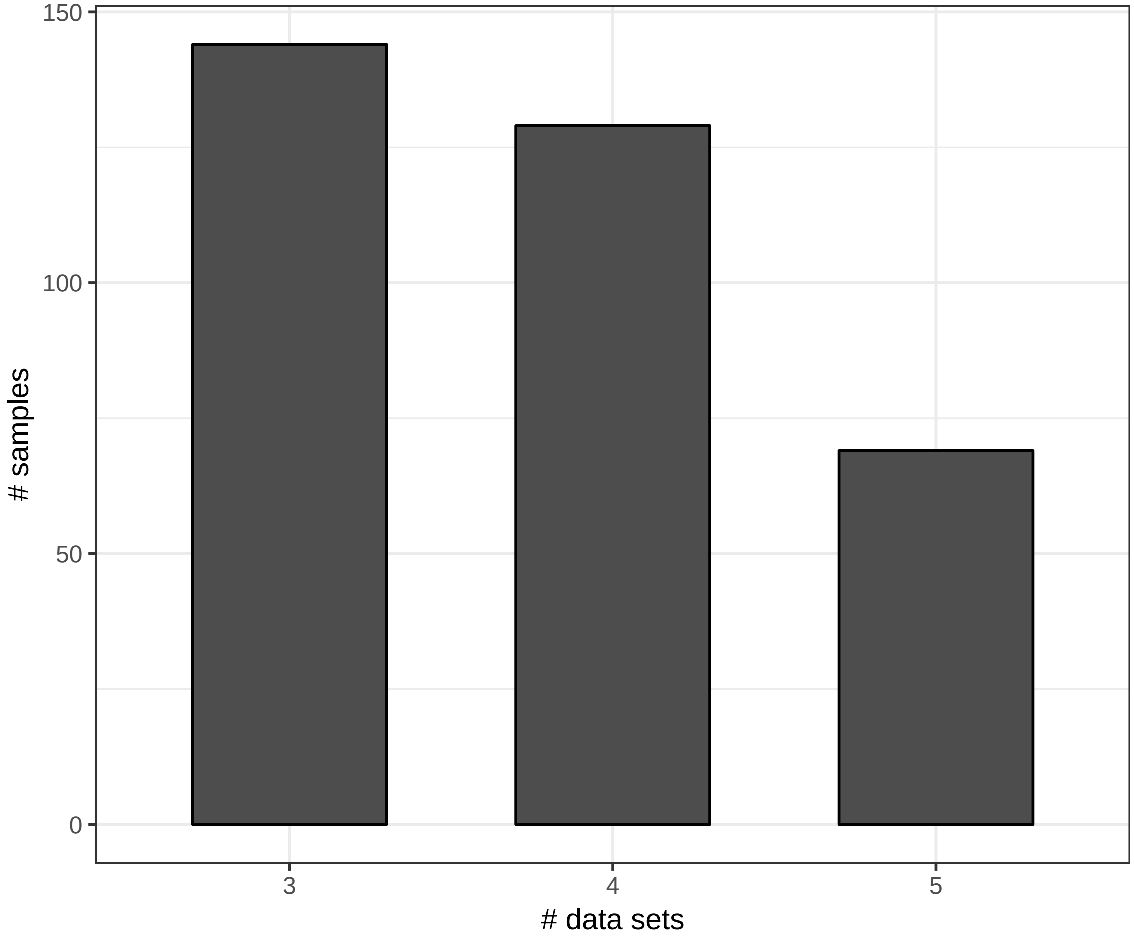

Figure 3: Distribution plot showing the number of shared samples across a given number of omics data sets.

```r
# setup visualization parameters
grp <- unique(dat_comb$Group)
grp <- grp[order(
  getsplit(grp, "-", 3),
  -as.numeric(factor(getsplit(grp, "-", 4))),
  c("Sham" = 1, "3R4F" = 2, "CHTP1.2" = 3, "THS2.2" = 4, "CESS" = 5, "SWITCH" = 6, "NA" = 7)[getsplit(grp, " ", 1)],
  getsplit(grp, " ", 2)
)]
grp
```

```
##  [1] "Sham 5m"    "Sham 6m"    "3R4F 5m"    "3R4F 6m"    "CHTP1.2 5m"
##  [6] "CHTP1.2 6m" "THS2.2 5m"  "THS2.2 6m"  "CESS 5m"    "CESS 6m"
## [11] "SWITCH 5m"  "SWITCH 6m"
```

```r
dat_comb$Group <- factor(dat_comb$Group, levels = grp)

cols <- attr(idmaps[["MRNA"]], "colors")
names(cols) <- names(idmaps[["MRNA"]])
cols2 <- cols[grep("6m", names(cols))]
names(cols2) <- gsub("6m", "5m", names(cols2))
cols <- c(cols, cols2)
cols3 <- c("blue", "blue")
names(cols3) <- c("Sham 5m", "Sham 6m")
cols <- c(cols, cols3)

ep_sel <- c(
  "Aerosol uptake and exposure|Nicotine metabolites in urine (absolute)|Total metabolites|nmol",
  "Aerosol uptake and exposure|Marker of exposure in urine (concentration)|3-HPMA|ng/mL",
  "Aerosol uptake and exposure|Marker of exposure in urine (concentration)|Total NNAL|pg/mL",
  "Aerosol uptake and exposure|Marker of exposure in urine (concentration)|SPMA|ng/mL",
  "Aerosol uptake and exposure|Marker of exposure in urine (concentration)|CEMA|ng/mL"
)

dat_comb <- dat_comb[dat_comb$EndpointMapped %in% ep_sel,]
range(dat_comb$N)
```

```
## [1] 10 12
```

```r
p_exposure <- list()
for (ep in ep_sel) {
  label <- getsplit(ep, "|", 3)
  unit <- getsplit(ep, "|", 4)

  p <- barchartNonOmicsMouse(dat_comb,
    ep = ep,
    laby = paste0(label, " ", unit),
    cols = cols
  )

  p_exposure[[label]] <- p

  filename <- file.path(reportDir, paste0("P15038_Urine_EXPOSURE-", label, ".pdf"))
  pdf(file = filename, width = 6, height = 6, useDingbats = FALSE)
  print(p)
  dev.off()
}
```

Figure 4 shows exposure markers measured in urine.

8 mRNA-protein correlations

```r
# mRNA vs protein correlation (group level)
idmapM <- lapply(idmaps[["MRNA"]], function(x) {
  x$nodeLabel <- as.character(x$nodeLabel)
  x[, c("nodeLabel", "foldChange", "adj.p.value", "p.value", "t", "df", "A", "TTT", "CTRL")]
})
idmapP <- lapply(idmaps[["PROTEIN"]], function(x) {
  x$nodeLabel <- as.character(x$nodeLabel)
  x[, c("nodeLabel", "foldChange", "adj.p.value", "p.value", "t", "df", "A", "TTT", "CTRL")]
})
stopifnot(all(names(idmapM) == names(idmapP)))

molM <- unique(unlist(lapply(idmapM, function(x) as.character(x$nodeLabel))))
molP <- unique(unlist(lapply(idmapP, function(x) as.character(x$nodeLabel))))
shared_mols <- intersect(molM, molP)
```

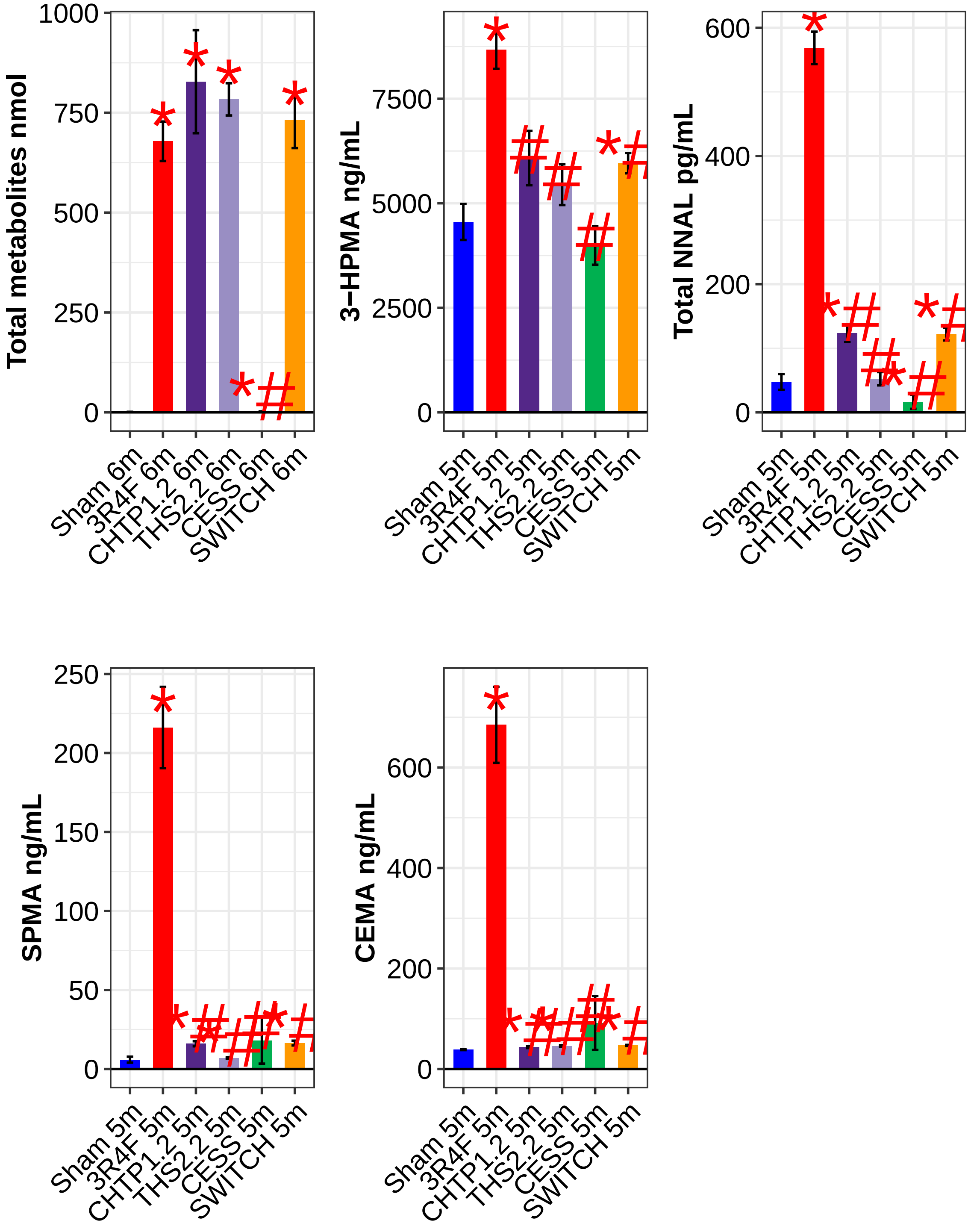

Figure 4: Exposure markers measured in urine.

```r
idmapM <- lapply(idmapM, function(x) {
  x[match(shared_mols, x$nodeLabel), ]
})
idmapP <- lapply(idmapP, function(x) {
  x[match(shared_mols, x$nodeLabel), ]
})

dat_list <- lapply(names(idmapM), function(x) {
  iM <- idmapM[[x]]
  iP <- idmapP[[x]]
  stopifnot(all(iM$nodeLabel == iP$nodeLabel))
  return(data.frame(Symbol = iM$nodeLabel,
                    fc_M = iM$foldChange,
                    sig_M = iM$adj.p.value < 0.05,
                    fc_P = iP$foldChange,
                    sig_P = iP$adj.p.value < 0.05,
                    Contrast = x))
})
dat <- do.call(rbind, dat_list)
dat$sig_summary <- "None"
dat$sig_summary[dat$sig_M == TRUE] <- "MRNA"
dat$sig_summary[dat$sig_P == TRUE] <- "PROTEIN"
dat$sig_summary[dat$sig_P == TRUE & dat$sig_M == TRUE] <- "BOTH"

dat$Treatment <- getsplit(dat$Contrast, " ", 1)
dat$Treatment <- factor(dat$Treatment, levels = c("3R4F", "CHTP1.2", "THS2.2", "CESS", "SWITCH"))
dat$Time <- getsplit(dat$Contrast, " ", 2)
dat$Time <- factor(dat$Time, levels = c("3m", "4m", "6m"))


res <- tapply(1:nrow(dat), dat$Contrast, function(i) {
  dat_sel <- dat[i, ]
  dat_sel <- dat_sel[dat_sel$sig_summary != "None", , drop = FALSE]
  if (nrow(dat_sel) > 10) {
    res.sum <- summary(lm(fc_P ~ fc_M, data = dat_sel))
    return(list(r.squared = res.sum$r.squared, intercept = res.sum$coefficients[1, 1], slope = res.sum$coefficients[2, 1]))
  } else {
    return(list(r.squared = NA, slope = NA, intercept = NA))
  }
})
stat_sum <- do.call(rbind, lapply(names(res), function(x) {
  data.frame(
    Contrast = x,
    Treatment = factor(getsplit(x, " ", 1), levels = c("3R4F", "CHTP1.2", "THS2.2", "CESS", "SWITCH")),
    Time = factor(getsplit(x, " ", 2), levels = c("3m", "4m", "6m")),
    R2 = res[[x]]$r.squared,
    intercept = res[[x]]$intercept,
    slope = res[[x]]$slope
  )
}))
stat_sum <- as.data.frame(stat_sum)
stat_sum$R2_txt <- paste0("R2 = ", sprintf("%2.2f", stat_sum$R2))
stat_sum$R2_txt[is.na(stat_sum$R2)] <- ""

p <- ggplot(aes(x = fc_M, y = fc_P, colour = sig_summary, size = sig_summary), data = dat)
p <- p + geom_point()
p <- p + scale_size_manual("", values = c("BOTH" = 2, "MRNA" = 2, "PROTEIN" = 2, "None" = 1), guide = FALSE)
p <- p + scale_colour_manual("", values = c("BOTH" = "firebrick2", "MRNA" = "slateblue1", "PROTEIN" = "palegreen4",
                                            "None" = "grey30"), guide = FALSE)
p <- p + geom_vline(xintercept = 0, size = 0.5, colour = "grey50")
p <- p + geom_hline(yintercept = 0, size = 0.5, colour = "grey50")
p <- p + geom_abline(aes(slope = slope, intercept = intercept),
                     colour = "steelblue",
                     size = 0.5,
                     data = stat_sum)
p <- p + geom_text(aes(label = R2_txt),
                   colour = "black",
                   x = 2,
                   y = 1.3,
                   hjust = 0,
                   size = 4, data =
                       stat_sum)
p <- p + facet_grid(Treatment ~ Time, drop = TRUE)
p <- p + theme_bw()
p <- p + labs(x = "log2 fold-change [MRNA]", y = "log2 fold-change [PROTEIN]")

p_scatter_mRNA_Prot <- p
png(file = file.path(reportDir, "P15038_LungManuscript_Scatter_mRNA_Prot.png"), width = 7, height = 9, units = "in", res = 200)
print(p)
dev.off()
```

```
## pdf
##   2
```

```r
p <- ggplot(aes(x = fc_M, y = fc_P, colour = sig_summary, size = sig_summary),
            data = droplevels(dplyr::filter(dat, Treatment == "3R4F")))
p <- p + geom_point()
p <- p + scale_size_manual("", values = c("BOTH" = 2, "MRNA" = 2, "PROTEIN" = 2, "None" = 1), guide = FALSE)
p <- p + scale_colour_manual("", values = c("BOTH" = "firebrick2", "MRNA" = "slateblue1", "PROTEIN" = "palegreen4",
                                            "None" = "grey30"), guide = FALSE)
p <- p + geom_vline(xintercept = 0, size = 0.5, colour = "grey50")
p <- p + geom_hline(yintercept = 0, size = 0.5, colour = "grey50")
p <- p + geom_abline(aes(slope = slope, intercept = intercept),
                     colour = "steelblue",
                     size = 0.5,
                     data = droplevels(dplyr::filter(stat_sum, Treatment == "3R4F")))
p <- p + geom_text(aes(label = R2_txt),
                   colour = "black",
                   x = 2,
                   y = 1.3,
                   hjust = 0,
                   size = 4,
                   data = droplevels(dplyr::filter(stat_sum, Treatment == "3R4F")))
p <- p + facet_grid(Time ~ Treatment, drop = TRUE)
p <- p + theme_bw()
p <- p + labs(x = "log2 fold-change [MRNA]", y = "log2 fold-change [PROTEIN]")

p_scatter_mRNA_Prot_3R4Fonly <- p

png(file = file.path(reportDir, "P15038_LungManuscript_Scatter_mRNA_Prot_3R4Fonly.png"),
    width = 3.5, height = 7, units = "in", res = 200)
print(p)
dev.off()
```

```
## pdf
##   2
```

Figure 5 shows the fold-change correlation between mRNA and protein expression, with Figure 6 focused on the 3R4F groups.

9 Biological impact factor (BIF) for lung

```r
npa_list = load2(file.path(RobjectsDir, "NPALIST_P15038-S185500_APOE_P2_Lung_Mm_MRNA.rda"))
```

```
## Loading objects:
##   npalist
```

```r
bif <- get_bif(npa_list)

cols = attr(idmaps[["MRNA"]], "colors")
names(cols) = names(idmaps[["MRNA"]])


filename = file.path(reportDir, "P15038_LungManuscript_BIF.pdf")
pdf(file = filename, width = 8,height=7)
  res <- barplot(bif,col = cols, mar = 8, pie = FALSE)
dev.off()
```

```
## pdf
##   2
```

```r
filename = file.path(reportDir, "P15038_LungManuscript_NPA_HEATMAP.pdf")
pdf(file = filename, width = 13,height=13)
  plot(npa_list)
dev.off()
```

```
## pdf
##   2
```

Figure 7 shows the biological impact on lung tissue using causal network enrichment approach and Figure 8 the corresponding NPA heatmap.

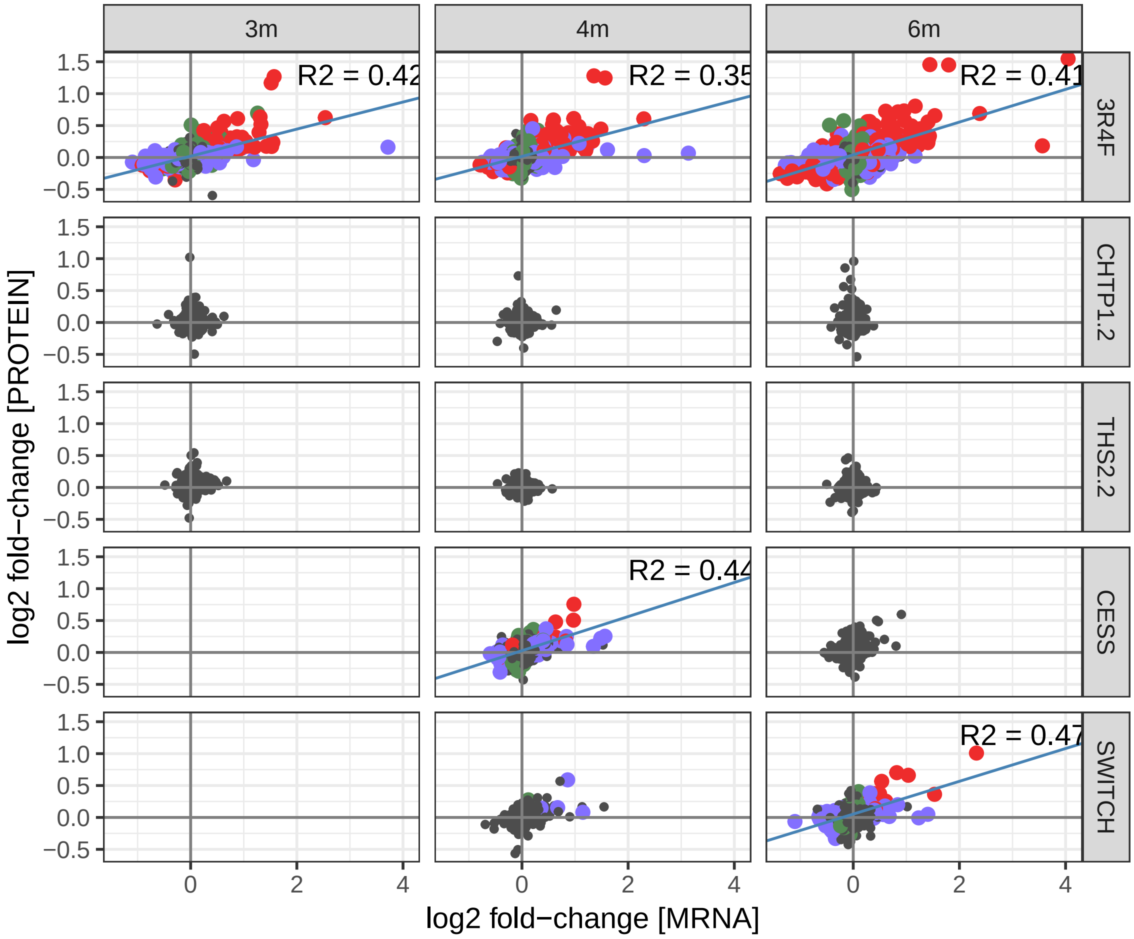

Figure 5: Correlation between mRNA and protein log2 fold-changes.

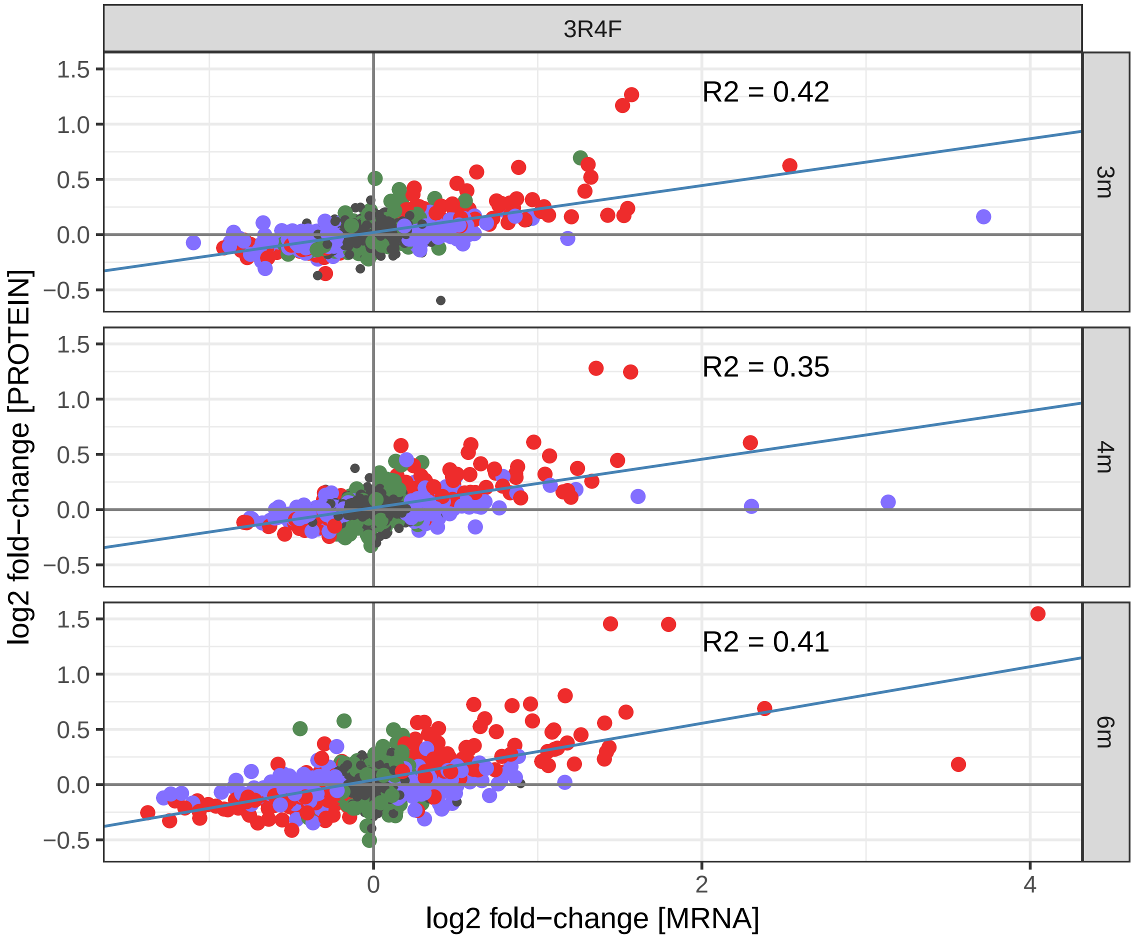

Figure 6: Correlation between mRNA and protein log2 fold-changes.

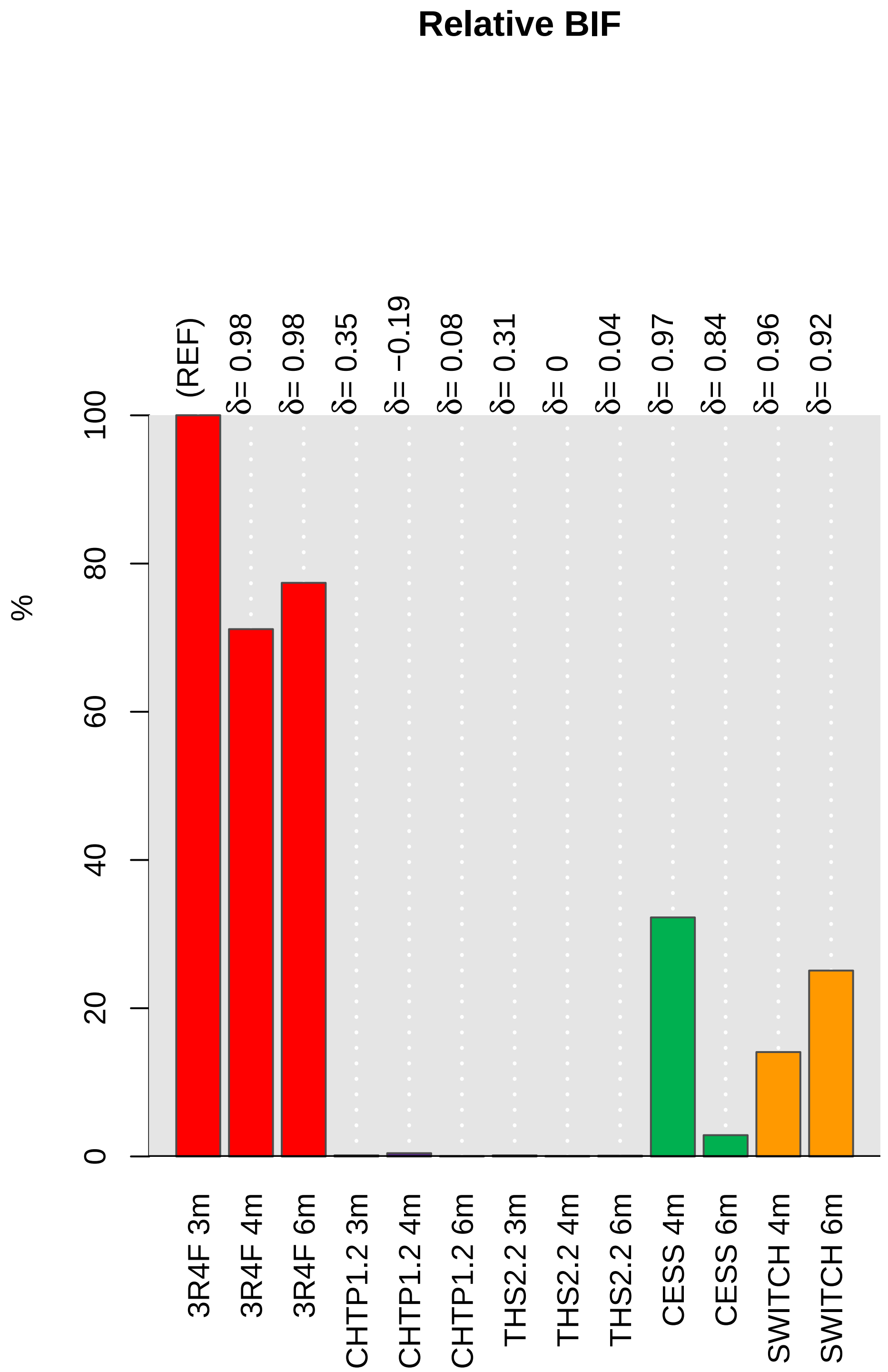

Figure 7: Evaluation of biological impact on lung tissue using causal network enrichment approach based on transcriptomics data. RBIFs are represented for each group versus Sham comparisons.

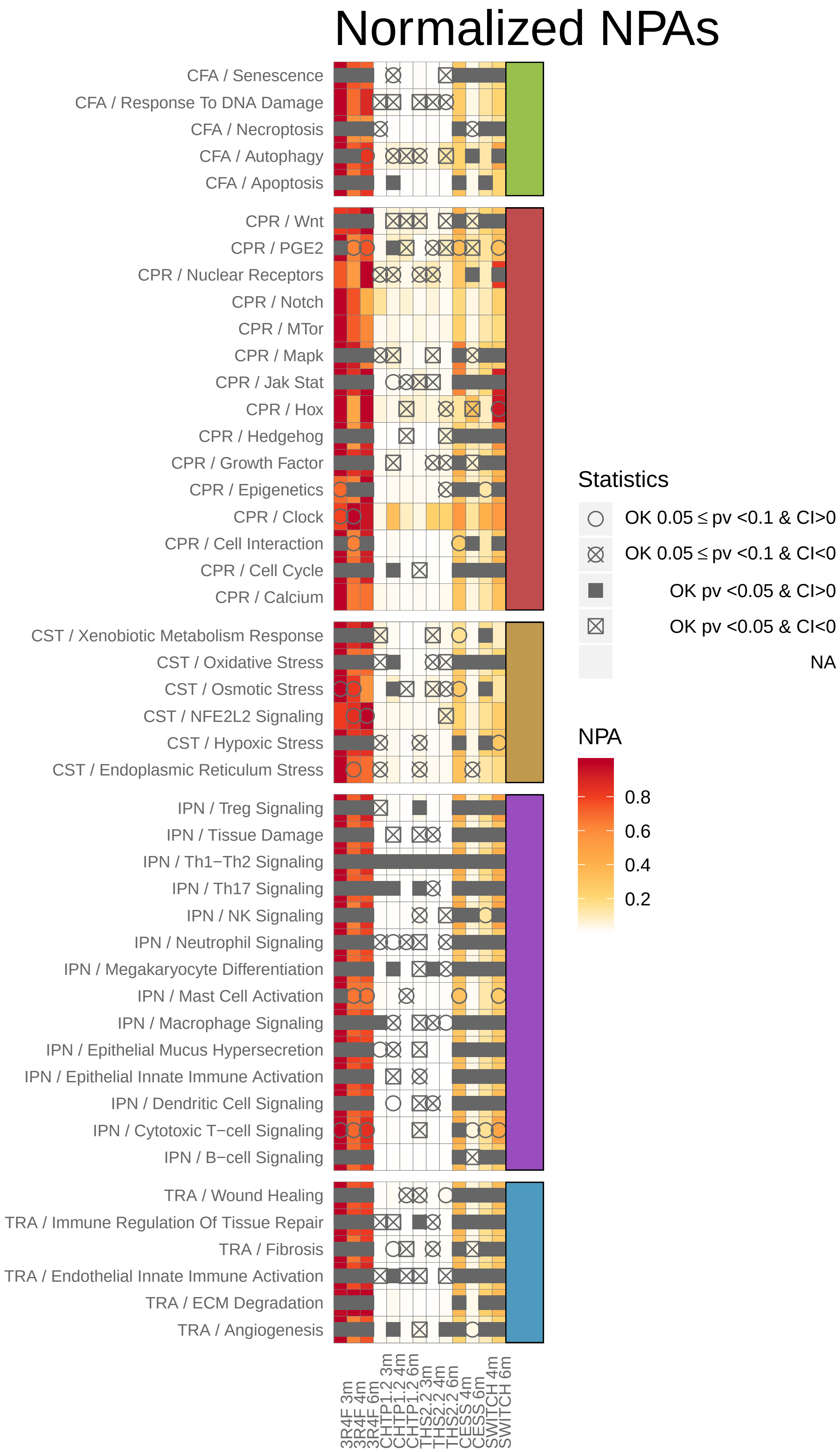

Figure 8: NPA heatmap. NPA heatmap for the lung. The heatmap shows NPAs for each network in the collection,

10 Principal component analysis (individual omes)

```
cols <- colgrps[,3]
names(cols) <- colgrps[,2]
pca_plots <- lapply(names(data_list), function(x) {
  view_dat <- t(data_list[[x]])
  sel <- apply(view_dat, 1, function(y) !all(is.na(y)))
  view_dat <- view_dat[sel, ]
  group <- dat_meta[match(rownames(view_dat), dat_meta$CAN), "Group"]
  cols2 <- cols[getsplit(group,"_",1)]
  names(cols2) <- group
  return(pcaGG(view_dat, group = factor(group), cols = cols2, main = x, print = FALSE, annotate = FALSE))
})
png(file = file.path(reportDir, "P15038_LungManuscript_PCA_ScorePlots2.png"), width = 11, height = 13, units = "in", res = 200)
do.call(gridExtra::grid.arrange, c(pca_plots, list(ncol = 2)))
dev.off()
```

```
## pdf
##   2
```

Figure 9 shows PCA score plots for the five omics data modalities.

11 Multi-omics factor analysis

11.1 Generate model

Perform multi-omics factor analysis for the five omice data modalities. Limit number of reported factors to reasonable number using a 2% DropFactorThreshold, otherwise go with default parameters.

```
MOFAobject <- createMOFAobject(data_list)

ModelOptions <- getDefaultModelOptions(MOFAobject)
ModelOptions
```

```
## $likelihood
##       MRNA      MIRNA    PROTEIN        MET      LIPID
## "gaussian" "gaussian" "gaussian" "gaussian" "gaussian"
##
## $numFactors
## [1] 72
##
## $sparsity
## [1] TRUE
```

```
TrainOptions <- getDefaultTrainOptions()
TrainOptions$seed <- 814279
TrainOptions$DropFactorThreshold <- 0.02
TrainOptions
```

```
## $maxiter
## [1] 5000
##
## $tolerance
## [1] 0.1
##
## $DropFactorThreshold
## [1] 0.02
##
## $verbose
## [1] FALSE
##
## $seed
## [1] 814279
```

```
DataOptions <- getDefaultDataOptions()
DataOptions
```

```
## $scaleViews
## [1] FALSE
##
## $removeIncompleteSamples
## [1] FALSE
```

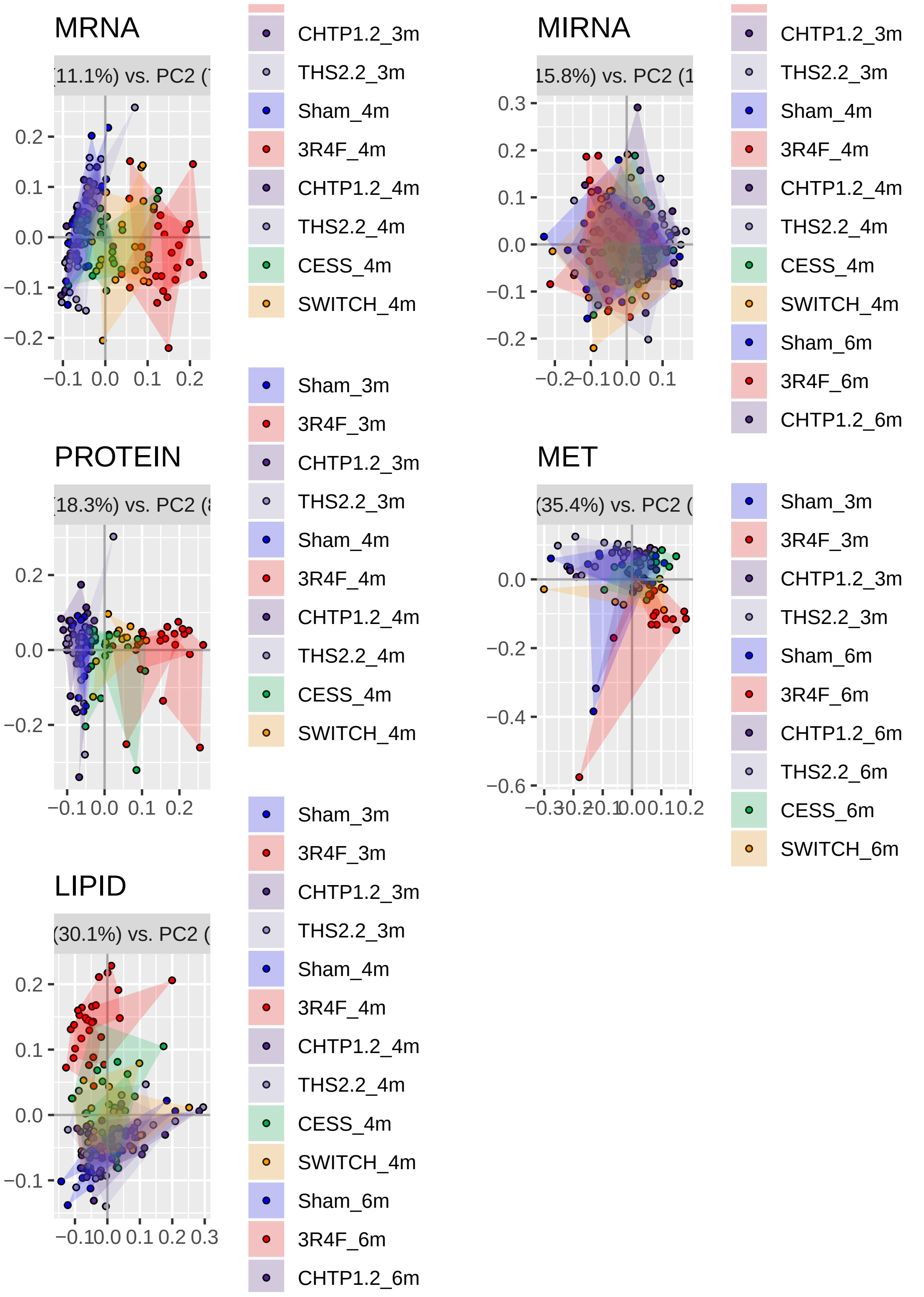

Figure 9: PCA score plots.

```
mofa_file <- file.path(reportDir, "P15038_LungManuscript_MOFA2.rda")
if (!force_rerun_mofa && file.exists(mofa_file)) {
    load(mofa_file)
} else {
  MOFAobject <- prepareMOFA(MOFAobject,
    DataOptions = DataOptions,
    ModelOptions = ModelOptions,
    TrainOptions = TrainOptions
  )
  MOFAobject <- runMOFA(MOFAobject)
  save(MOFAobject, file = mofa_file)
}
```

```
## [1] "No output file provided, using a temporary file..."
```

11.2 Explained variance, score, and loading plots

```
p_var_explained <- plotVarianceExplained(MOFAobject)
pdf(file = file.path(reportDir, "P15038_LungManuscript_MOFA_VarianceExplained.pdf"), width = 7, height = 7)
    print(p_var_explained)
dev.off()
```

```
## pdf
##   2
```

```
# alternative stacked barplot for explained variance
R2_list <- calculateVarianceExplained(MOFAobject)
fvar_m <- R2_list$R2Total
fvar_mk <- R2_list$R2PerFactor
plotdat <- reshape2::melt(fvar_mk)
colnames(plotdat) <- c("LF", "View", "ExpVar")
p <- ggplot(aes(x = factor(View, levels = c("MRNA", "PROTEIN", "MET", "MIRNA", "LIPID")), y = ExpVar, fill = LF), data = plotdat)
p <- p + geom_bar(stat = "identity", width = 0.5)
p <- p + scale_fill_brewer("LF", palette = "Spectral")
p <- p + theme_bw()
p <- p + theme(axis.text.x = element_text(angle = 45, hjust = 1, vjust = 1))
p <- p + theme(plot.title = element_text(hjust = 0.5))
p <- p + labs(x = "", y = "explained variance", title = "MOFA")
p_var_explained_bars <- p

pdf(file = file.path(reportDir, "P15038_LungManuscript_MOFA_VarianceExplainedBars.pdf"), width = 5, height = 5)
    plot(p_var_explained_bars)
dev.off()
```

```
## pdf
##   2
```

```
# export weights
res <- MOFA::getWeights(MOFAobject)
fn <- file.path(reportDir, "P15038_LungManuscript_MOFA_AllWeights.rda")
save(res, file = fn)

# Top weights
pdf(file = file.path(reportDir, "P15038_LungManuscript_MOFA_TopWeights.pdf"), width = 9, height = 8)
for (data_type in c("PROTEIN", "MRNA", "MIRNA", "LIPID", "MET")) {
  for (LF in 1:3) {
    plot(plotTopWeights(MOFAobject, data_type, LF, nfeatures = 30) + labs(title = data_type))
  }
}
dev.off()
```

```
## pdf
##   2
```

```
# Scatter Pairs
grp <- sapply(strsplit(as.character(dat_meta$Group), "_"), function(x) x[[1]])
tp <- sapply(strsplit(as.character(dat_meta$Group), "_"), function(x) x[[2]])

cols <- colgrps[match(unique(grp), colgrps[, 2]), "Color"]
names(cols) <- unique(grp)

p <- plotFactorScatters2(MOFAobject, factors = 1:10, color_by = grp, shape_by = factor(tp), col_values = cols)
p_scatter_pairs <- p

pdf(file = file.path(reportDir, "P15038_LungManuscript_MOFA_FactorsScatterPairs.pdf"), width = 15, height = 15)
print(p_scatter_pairs)
dev.off()
```

```
## pdf
```

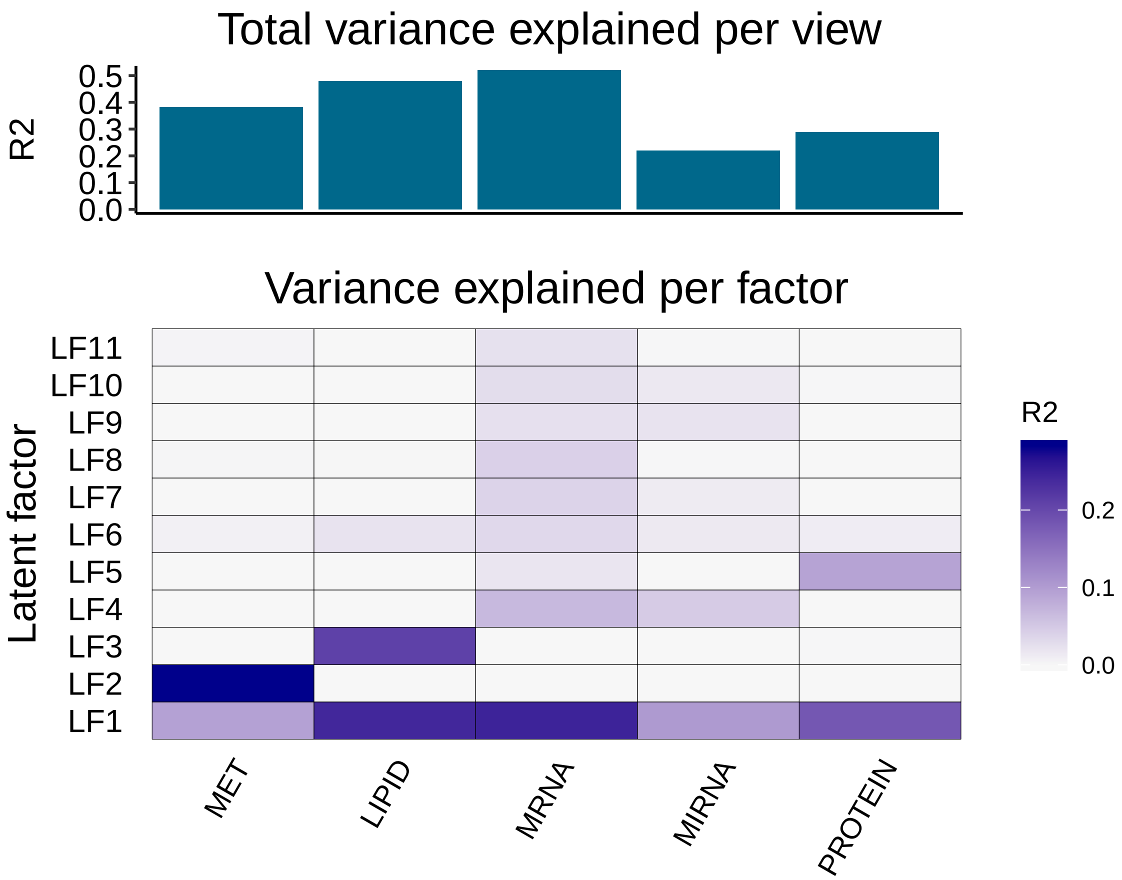

Figure 10: Explained variance across omics data modalities and latent factors.

```
##   2
```

```
# LF1 score beeswarm
Z <- getFactors(MOFAobject,
  factors = 1,
  as.data.frame = TRUE
)
Z$Group <- dat_meta[match(Z$sample, dat_meta$CAN), "Group"]
Z$Treatment <- getsplit(Z$Group, "_", 1)
Z$Time <- getsplit(Z$Group, "_", 2)

p <- ggplot(aes(x = 0, y = value, colour = Treatment, shape = Time), data = Z)
p <- p + ggbeeswarm::geom_quasirandom()

cols <- colgrps[match(unique(Z$Treatment), colgrps[, 2]), "Color"]
names(cols) <- unique(Z$Treatment)

p <- p + scale_colour_manual("Treatment", values = cols)
p <- p + theme_bw()
p <- p + theme(
  axis.text.x = element_blank(),
  axis.ticks.x = element_blank(),
  plot.title = element_text(hjust = 0.5)
)
p <- p + labs(title = "MOFA", x = "", y = "score LF1")
p_LF1_score_beeswarm <- p

pdf(file = file.path(reportDir, "P15038_LungManuscript_MOFA_LF1_ScoresByGroup.pdf"), width = 3.5, height = 3.5, useDingbats = FALSE)
plot(p_LF1_score_beeswarm)
dev.off()
```

```
## pdf
##   2
```

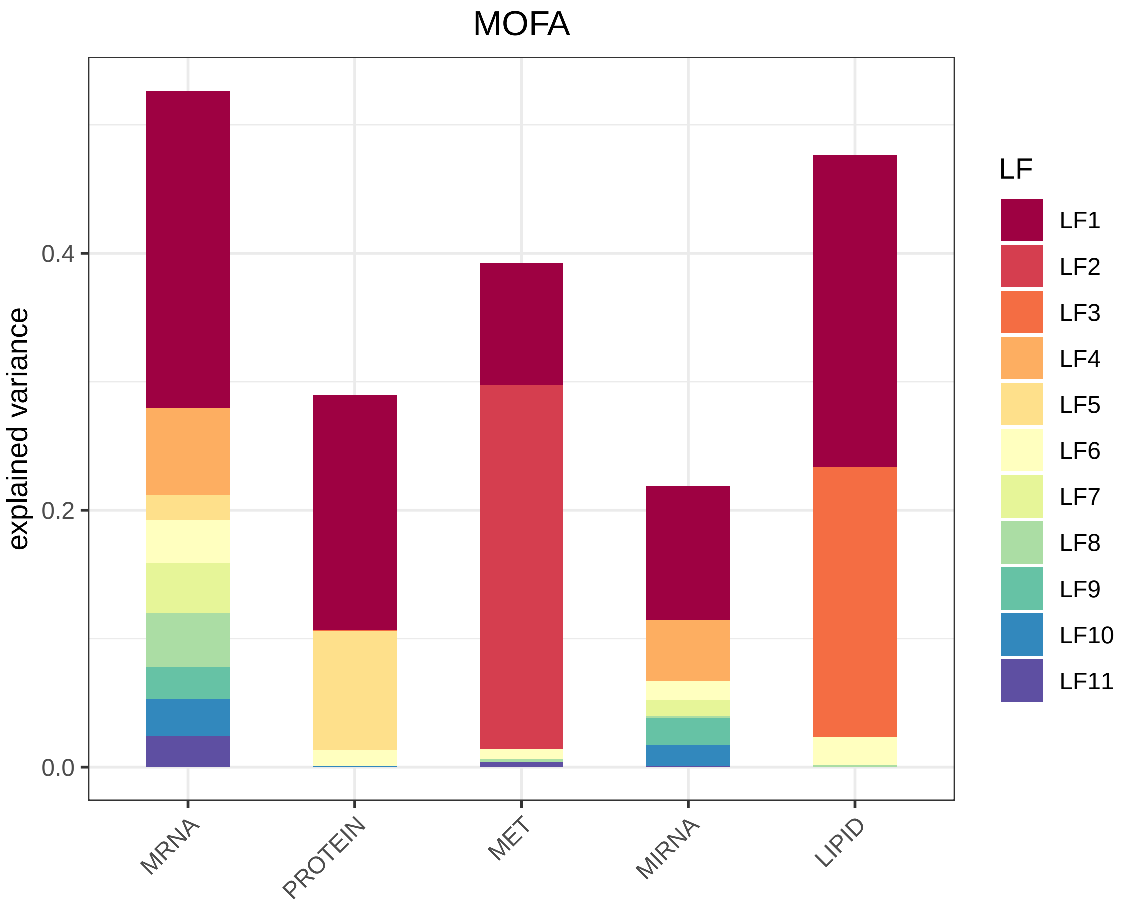

Figure 11: Explained variance across omics data modalities and latent factors.

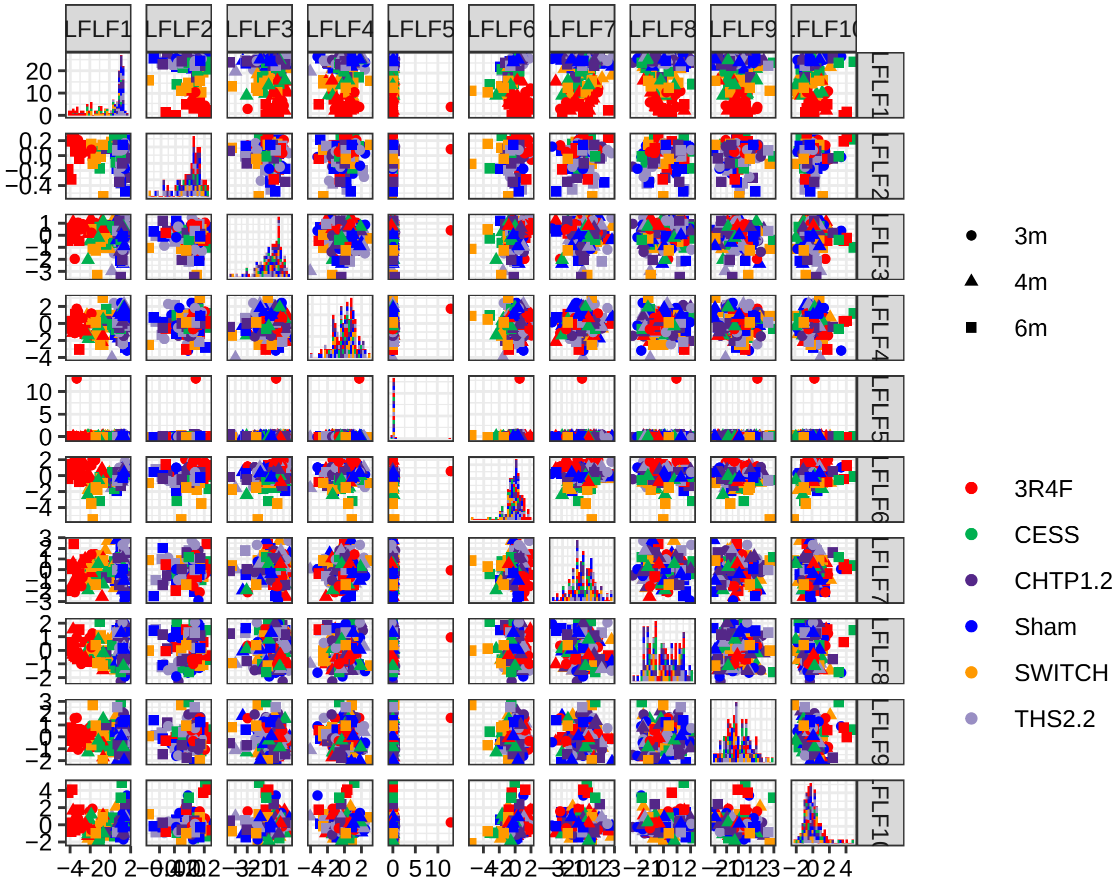

Figure 12: LF score pairs.

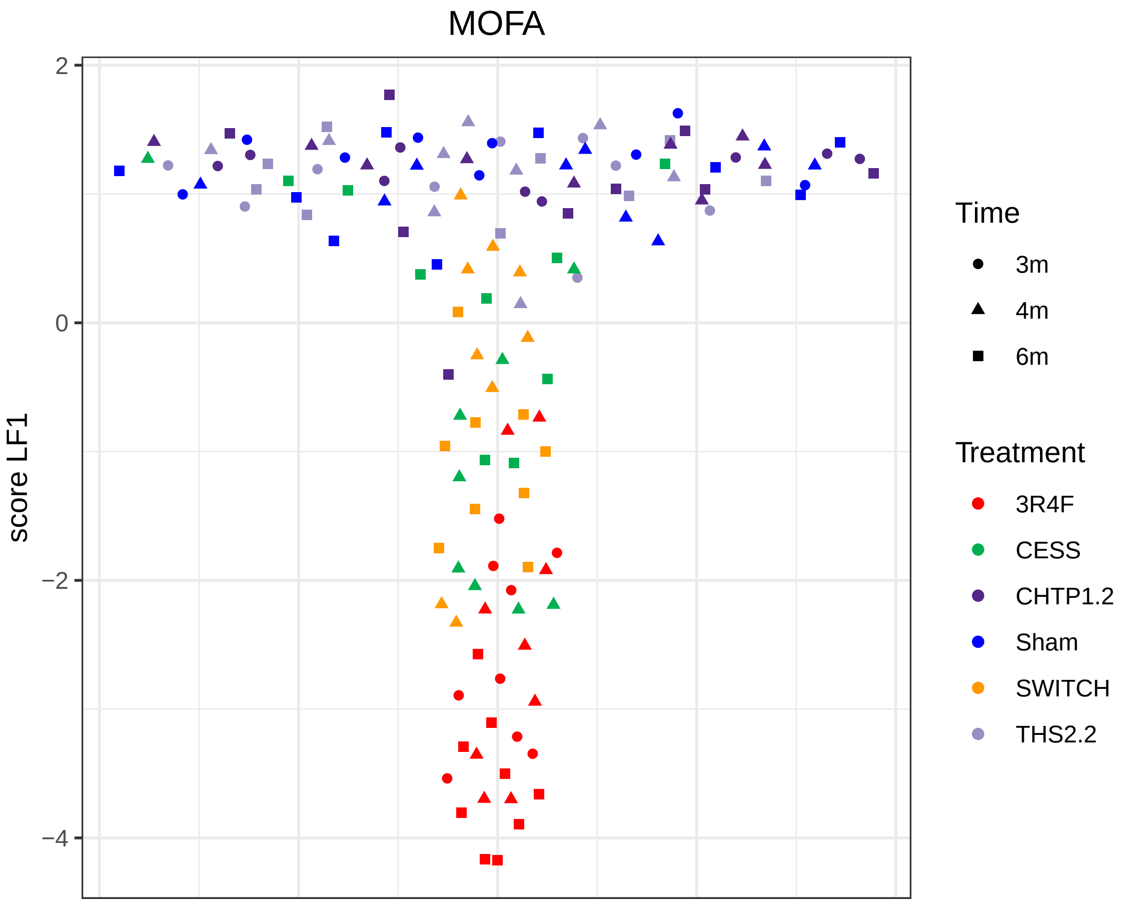

Figure 13: LF1 scores.

Figure 10 and Figure 11 show variance explained by the MOFA model across the data modalities and discovered latent factors (LFs). Figure 12 shows the pair-wise LF score plots, highlighting LF1 as the LF that separates the exposure groups. Figure 13 shows the scores for LF1.

11.3 GSEA for biological interpretation of LF1

```
# functional annotation --> GSEA
gsc_file <- readLines(file.path("..","DATA", "EXTERNAL", "ReactomePathways_23Apr2018.gmt"))
gsc <- do.call(rbind, lapply(gsc_file, function(x) {
  ss <- strsplit(x, "\t", fixed = TRUE)[[1]]
  genes <- ss[seq(3, length(ss))]
  gs <- ss[1]
  data.frame(Gene = genes, GS = gs)
}))
gsc <- piano::loadGSC(gsc)

weight_list <- MOFA::getWeights(MOFAobject)
fgsea.res <- lapply(c("MRNA", "PROTEIN"), function(view) {
  geneLevelStats <- weight_list[[view]][, "LF1"]
  names(geneLevelStats) <- getsplit(names(geneLevelStats), "_", 1)
  names(geneLevelStats) <- GetOrtholog(names(geneLevelStats), species_from = "Mm", species_to = "Hs")
  geneLevelStats <- geneLevelStats[!is.na(names(geneLevelStats))]
  geneLevelStats <- tapply(geneLevelStats, names(geneLevelStats), function(x) x[which.max(abs(x))])

  set.seed(23489)
  piano.res <- piano::runGSA(
    geneLevelStats = geneLevelStats,
    geneSetStat = "fgsea",
    signifMethod = "geneSampling",
    adjMethod = "fdr",
    ncpus = 1,
    gsc = gsc,
    gsSizeLim = c(5, Inf)
  )
  piano::GSAsummaryTable(piano.res)
})
```

```
## Loading objects:
##   HCOP_ORTHOLOGY_TABLE
## Checking arguments...done!
## Calculating gene set statistics...done!
## Calculating gene set significance...done!
## Adjusting for multiple testing...done!
## Loading objects:
##   HCOP_ORTHOLOGY_TABLE
## Checking arguments...done!
## Calculating gene set statistics...done!
## Calculating gene set significance...done!
## Adjusting for multiple testing...done!
```

```
names(fgsea.res) <- c("MRNA", "PROTEIN")
filename <- file.path(reportDir, "P15038_LungManuscript_MOFA_LF1_FGSEA.rda")
save(fgsea.res, file = filename)

# tables
for (view in c("MRNA", "PROTEIN")) {
  filename <- file.path(reportDir, paste0("P15038_LungManuscript_MOFA_FGSEA_LF1_", view, ".csv"))
  write.csv(fgsea.res[[view]], file = filename)
}

# figures
p_MOFA_GSEA_figures <- list()
for (view in c("MRNA", "PROTEIN")) {
  tab <- fgsea.res[[view]]
  tab <- tab[tab$"p adj (dist.dir.up)" < 0.05 | tab$"p adj (dist.dir.dn)" < 0.05, ]
  tab$pval <- ifelse(!is.na(tab$"p (dist.dir.up)"), tab$"p (dist.dir.up)", tab$"p (dist.dir.dn)")
  sort_order <- order(tab$pval, decreasing = FALSE)
  tab <- tab[sort_order, ]
  tab <- tab[1:20, ]
  colnames(tab)[3] <- "Stat"
  tab$Name <- factor(tab$Name, levels = rev(tab$Name))

  p <- ggplot(aes_string(x = "Name", y = "Stat", fill = "pval"), data = tab)
  p <- p + geom_bar(stat = "identity")
  # p = p + scale_y_log10()
  p <- p + ggplot2::coord_flip()
  p <- p + theme_bw()
```

```
  p <- p + labs(y = "Stat", x = "", title = view)
  p <- p + theme(axis.text = element_text(size = 8, colour = "black"))
  p <- p + theme(axis.title = element_text(size = 8, colour = "black", face = "bold"))
  p_MOFA_GSEA_figures[[view]] <- p

  filename <- file.path(reportDir, paste0("P15038_LungManuscript_MOFA_FGSEA_LF1_", view, ".pdf"))
  pdf(file = filename, width = 7, height = 5)
  print(p)
  dev.off()
}
```

Figure 14 shows the TOP 20 GSEA-enriched gene sets by p-value for the Reactome collection.

11.4 LF1 vs inflammatory cells in BALF

```
## correlate / compare scores with total cells in BALF (group averages)
stat_dat <- load2(file.path("../","DATA", "P15038_APOE_P2_BALF_cells.rda"))
```

```
## Loading objects:
##   stat_dat
```

```
# head(stat_dat)
stat_dat <- stat_dat[stat_dat$Endpoint_Sub_Cat == "FLC differentiation (count)" &
                         stat_dat$Endpoint_Name == "Total cells", ]
stat_dat$Group <- paste0(gsub("Switch CHTP", "SWITCH",
                              gsub("Cessation", "CESS", stat_dat$Treatment_Order)), "_",
                         gsub("M", "m", stat_dat$Dissection_Order))
unique(stat_dat$Group)
```

```
##  [1] "Sham_2m"    "3R4F_2m"    "CHTP1.2_2m" "THS2.2_2m"  "Sham_3m"
##  [6] "3R4F_3m"    "CHTP1.2_3m" "THS2.2_3m"  "Sham_4m"    "3R4F_4m"
## [11] "CHTP1.2_4m" "THS2.2_4m"  "CESS_4m"    "SWITCH_4m"  "Sham_6m"
## [16] "3R4F_6m"    "CHTP1.2_6m" "THS2.2_6m"  "CESS_6m"    "SWITCH_6m"
```

```
Z <- getFactors(MOFAobject,
  factors = 1,
  as.data.frame = TRUE
)
Z$Group <- dat_meta[match(Z$sample, dat_meta$CAN), "Group"]
Z$Treatment <- getsplit(Z$Group, "_", 1)
Z$Time <- getsplit(Z$Group, "_", 2)

stopifnot(all(Z$Group %in% stat_dat$Group))

Z_avg <- Z %>% dplyr::group_by(Group, Treatment, Time) %>% dplyr::summarise(mean_score = mean(value, na.rm = TRUE))
Z_avg <- as.data.frame(Z_avg)
Z_avg$TotalCells <- stat_dat[match(Z_avg$Group, stat_dat$Group), "Endpoint_Value_Mean"]

p <- ggplot(aes(x = mean_score, y = TotalCells), data = Z_avg)
p <- p + geom_point(aes(colour = Treatment, shape = Time), size = 2)
p <- p + geom_smooth(method = "lm", data = Z_avg, formula = y ~ x)
p <- p + theme_bw()
p <- p + labs(x = "mean MOFA score [comp. 1]", y = "mean Total cells in BALF [10E5]")

cols <- colgrps[, "Color"]
names(cols) <- colgrps[, "Label"]
p <- p + scale_colour_manual("Treatment", values = cols)

p_MOFA_BALF_correlation <- p

filename <- file.path(reportDir, "P15038_LungManuscript_MOFA_TotalCells_Scatter.pdf")
pdf(file = filename, width = 4.4, height = 3.5, useDingbats = FALSE)
print(p_MOFA_BALF_correlation)
dev.off()
```

```
## pdf
##   2
```

Figure 15 compares average LF1 and BALF cell counts across the exposure groups.

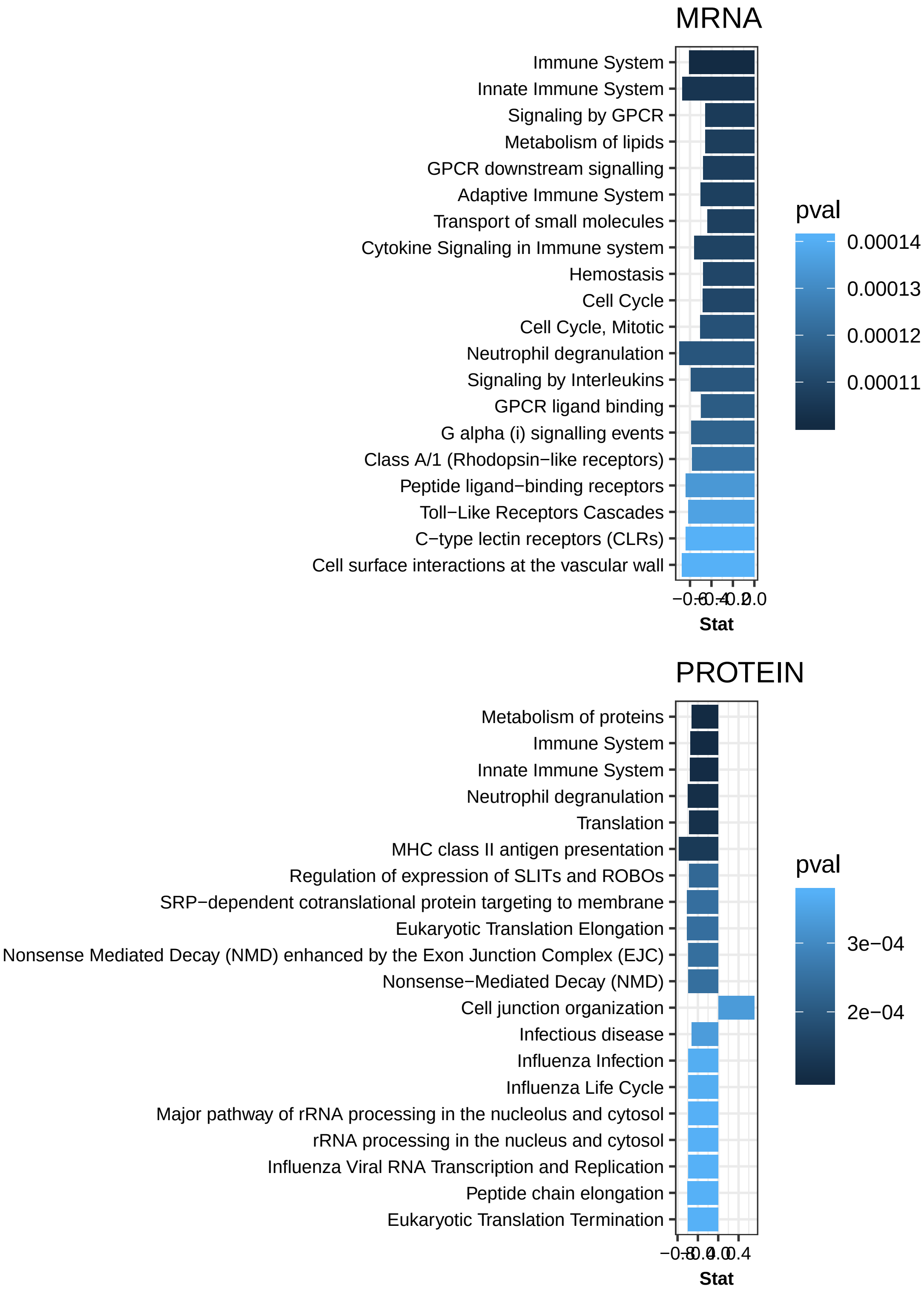

Figure 14: LF1 scores.

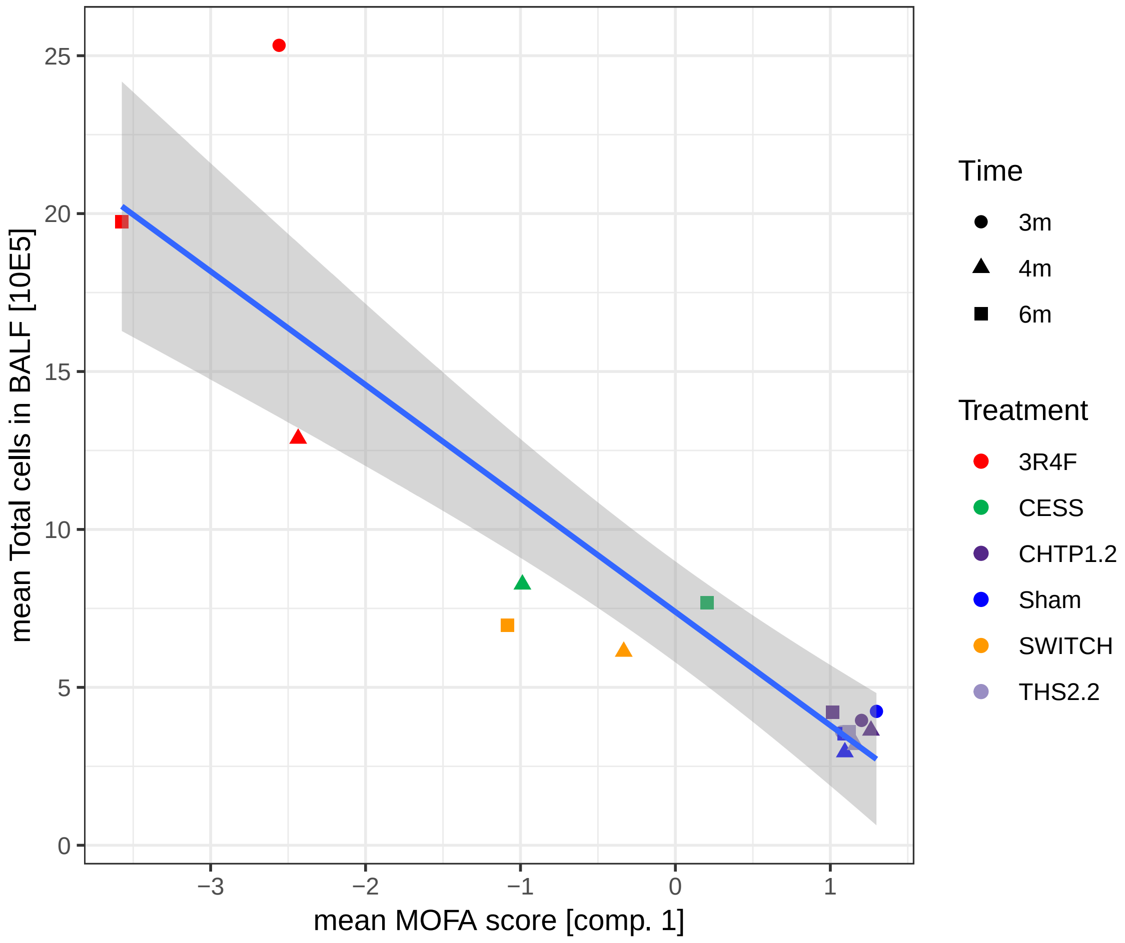

Figure 15: LF1 vs BALF cells.

12 sGCCA: complement MOFA approach

```
# for definition of data_list, see above
data_list2 <- lapply(data_list, function(x) t(x))
lapply(data_list2, dim)
```

```
## $MRNA
## [1]   144 17473
##
## $MIRNA
## [1] 144 363
##
## $PROTEIN
## [1]  144 1395
##
## $MET
## [1] 144 674
##
## $LIPID
## [1] 144 399
```

```
# design matrix for sGCCA
design <- matrix(1,
  ncol = length(data_list), nrow = length(data_list),
  dimnames = list(names(data_list), names(data_list))
)
diag(design) <- 0
design
```

```
##         MRNA MIRNA PROTEIN MET LIPID
## MRNA       0     1       1   1     1
## MIRNA      1     0       1   1     1
## PROTEIN    1     1       0   1     1
## MET        1     1       1   0     1
## LIPID      1     1       1   1     0
```

```
ncomp <- 8
# set number of variables to select, per component and per data set (fixed to 30)
list.keepX <- list(MRNA = rep(30, ncomp),
                   MIRNA = rep(30, ncomp),
                   PROTEIN = rep(30, ncomp),
                   MET = rep(30, ncomp),
                   LIPID = rep(30, ncomp))

set.seed(342897)
res.block.spls <- block.spls(
  X = data_list2, indY = 4,
  ncomp = ncomp, keepX = list.keepX, design = design, mode = "canonical",
  max.iter = 1000
)
# res.block.spls

res <- selectVar(res.block.spls, comp = 1)

# stacked barplot for explained variance
fvar_mk <- do.call(cbind, res.block.spls$explained_variance)
rownames(fvar_mk) <- gsub("comp ", "", rownames(fvar_mk))
plotdat <- reshape2::melt(fvar_mk)
colnames(plotdat) <- c("LF", "View", "ExpVar")
p <- ggplot(aes(x = factor(View, levels = c("MRNA", "PROTEIN", "MET", "MIRNA", "LIPID")),
                y = ExpVar, fill = factor(LF, levels = 1:8)), data = plotdat)
p <- p + geom_bar(stat = "identity", width = 0.5)
p <- p + scale_fill_brewer("LF", palette = "Spectral")
p <- p + theme_bw()
p <- p + theme(axis.text.x = element_text(angle = 45, hjust = 1, vjust = 1))
p <- p + theme(plot.title = element_text(hjust = 0.5))
p <- p + labs(x = "", y = "explained variance", title = "sGCCA")
p_sGCCA_expl_var <- p

pdf(file = file.path(reportDir, "P15038_LungManuscript_sGCCA_VarianceExplainedBars.pdf"), width = 3, height = 3.2)
plot(p_sGCCA_expl_var)
dev.off()
```

```
## pdf
##   2
```

```
#score plots
groups <- dat_meta[match(rownames(data_list2[[1]]), dat_meta$"CAN"), "Group"]
TTT <- factor(getsplit(groups, "_", 1), levels = colgrps[, 2])
TIME <- factor(getsplit(groups, "_", 2))
```

```
fn <- file.path(reportDir, "P15038_LungManuscript_sGCCA_SCORE-PLOT.pdf")
pdf(file = fn, width = 12, height = 9, useDingbats = FALSE)
plotIndiv(res.block.spls,
            group = TTT,
            col.per.group = colgrps[match(levels(TTT), colgrps[, 2]), "Color"],
            pch = TIME,
            ind.names = FALSE,
            legend = TRUE,
            ellipse = FALSE
            )
dev.off()
```

```
## pdf
##   2
```

```
# LF score beeswarm for each block
Z <- do.call(rbind, lapply(names(res.block.spls$variates), function(x) {
  vars <- res.block.spls$variates[[x]]
  data.frame(block = x, sample = rownames(vars), value = as.numeric(vars[, "comp 1"]))
}))
Z$Group <- dat_meta[match(Z$sample, dat_meta$CAN), "Group"]
Z$Treatment <- getsplit(Z$Group, "_", 1)
Z$Time <- getsplit(Z$Group, "_", 2)
Z$block <- factor(Z$block, levels = c("MRNA", "PROTEIN", "MET", "MIRNA", "LIPID"))

p <- ggplot(aes(x = 0, y = value, colour = Treatment, shape = Time), data = Z)
p <- p + ggbeeswarm::geom_quasirandom()

cols <- colgrps[match(unique(Z$Treatment), colgrps[, 2]), "Color"]
names(cols) <- unique(Z$Treatment)
p <- p + scale_colour_manual("Treatment", values = cols)

p <- p + facet_wrap(~block, ncol = 2)

p <- p + theme_bw()
p <- p + theme(
  axis.text.x = element_blank(),
  axis.ticks.x = element_blank(),
  plot.title = element_text(hjust = 0.5)
)
p <- p + labs(title = "sGCCA", x = "", y = "score LF1")
p_sGCCA_LF1_beeswarm <- p

pdf(file = file.path(reportDir, "P15038_LungManuscript_sGCCA_LF1_ScoresByGroup.pdf"),
    width = 7, height = 7, useDingbats = FALSE)
plot(p_sGCCA_LF1_beeswarm)
dev.off()
```

```
## pdf
##   2
```

```
# illustrates coefficient weights in each block
fn <- file.path(reportDir, "P15038_LungManuscript_sGCCA_LOADINGS-PLOT.pdf")
pdf(file = fn, width = 12, height = 12)
plotLoadings(res.block.spls, ncomp = 1)
dev.off()
```

```
## pdf
##   2
```

Figure 16 shows the score plots for the sGCCA appraoch, with Figure 17 focussing on the LF1 scores.

13 MOFA vs sGCCA

```
## compare loadings / weights between SGCCA and MOFA
weights_sgcca <- selectVar(res.block.spls, comp = 1)
fn <- file.path(reportDir, "P15038_LungManuscript_MOFA_AllWeights.rda")
weights_mofa <- load2(fn)
```

```
## Loading objects:
##   res
```

```
# compare score rankings
plots <- list()
res_list <- list()
for (view in names(weights_mofa)) {
  dat <- weights_sgcca[[view]]$value
```

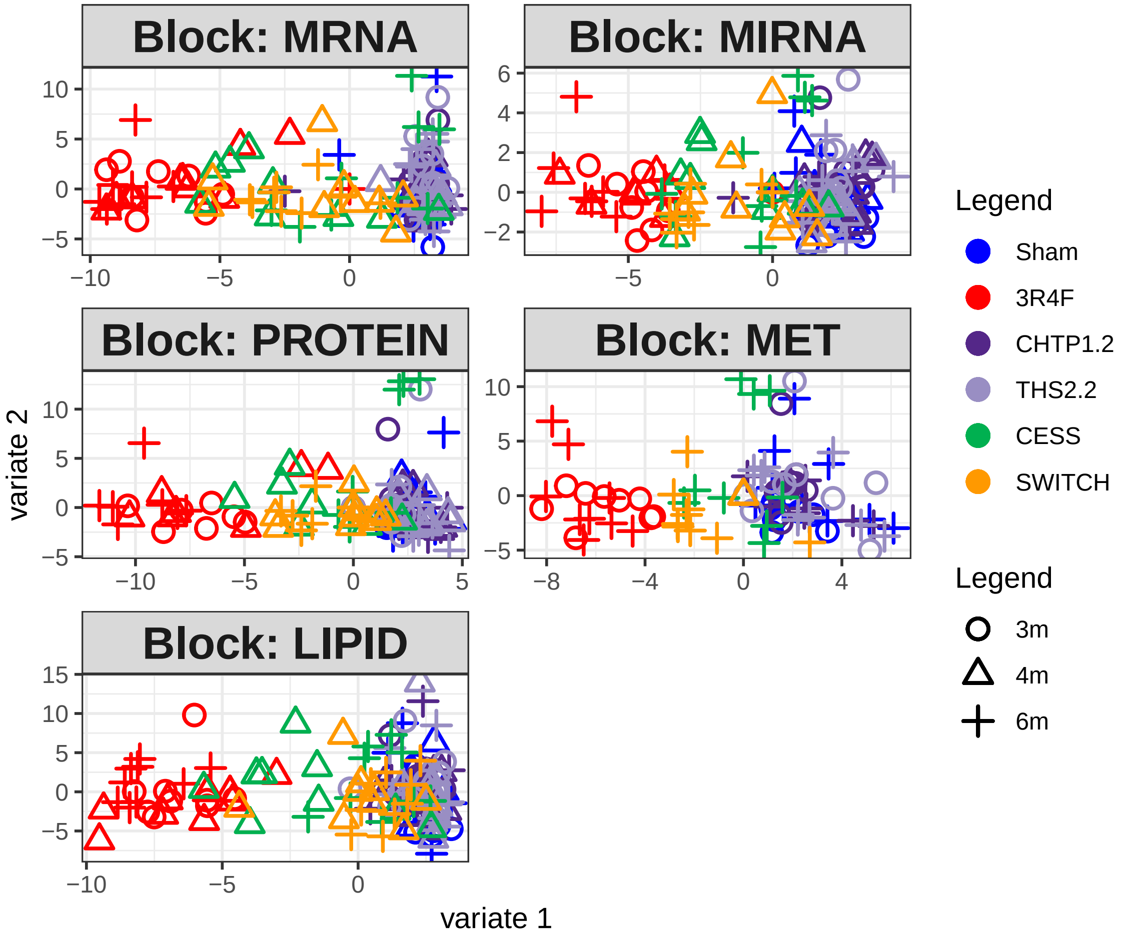

Figure 16: sGCCA score plots.

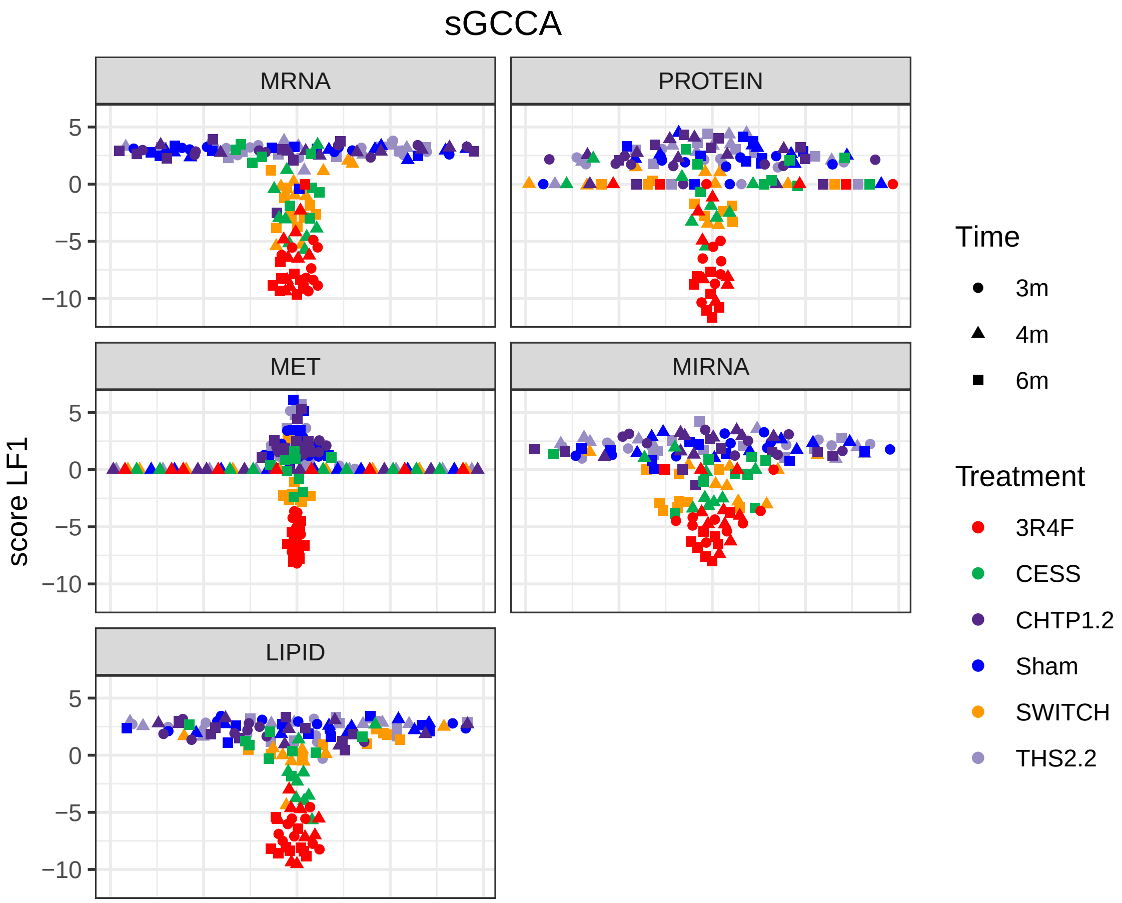

Figure 17: sGCCA score plot for LF1.

```r
  dat$rank_sgcca <- sign(dat$value.var) * order(abs(dat$value.var), decreasing = TRUE)

  tmp <- weights_mofa[[view]][order(abs(weights_mofa[[view]][, "LF1"]), decreasing = TRUE), , drop = FALSE]
  tmp <- data.frame(tmp)
  tmp$rank <- sign(tmp[, "LF1"]) * seq_len(nrow(tmp))

  dat$rank_mofa <- tmp$rank[match(rownames(dat), rownames(tmp))]

  dat$x <- abs(dat$rank_sgcca)
  dat$y <- abs(dat$rank_mofa)
  dat$x_sign <- sign(dat$rank_sgcca)
  dat$y_sign <- sign(dat$rank_mofa)
  dat$xy_sign <- ifelse(dat$x_sign * dat$y_sign == 1, "same", "opposite")

  p <- ggplot(aes(x = x, y = y, colour = xy_sign), data = dat)
  p <- p + geom_point(size = 2)
  p <- p + scale_colour_manual("Sign", values = c("same" = "red", "opposite" = "blue"))
  p <- p + theme_bw()
  p <- p + labs(x = "rank sGCCA [LF1]", y = "rank MOFA [LF1]", title = view)

  plots[[view]] <- p

  tmp <- dat[, c("rank_sgcca", "rank_mofa")]
  tmp$analyte <- rownames(tmp)
  tmp$view <- view
  res_list[[view]] <- tmp
}

filename <- file.path(reportDir, "P15038_LungManuscript_MOFA_sGCCA_Comparison.pdf")
pdf(file = filename, width = 8, height = 10, useDingbats = FALSE)
gridExtra::grid.arrange(grobs = plots, cols = 2)
dev.off()
```

```
## pdf
##   2
```

```r
res_dat <- do.call(rbind, res_list)
filename <- file.path(reportDir, "P15038_LungManuscript_MOFA_sGCCA_Comparison.csv")
write.csv(res_dat, file = filename, row.names = FALSE)
```

Figure 18 compares the loadings between the MOFA and sGCCA approach.

14 Network-assisted interpretation of LF1

14.1 Create network

```r
if (force_recreate_network) {
    #  * Sub-Section * Create metabolite network
    # ~~~~~~~~~~~~~~~~~~~~~~~~~~~~~~~~~~~~~~~~~~~~~~~~~~~~~~~~~~~~~~~~~~~~~~~~~~~

    # Prepare KEGG
    reaction_dat <- readr::read_delim("../DATA/EXTERNAL/reaction_mapformula.lst", " ", col_names = FALSE) %>% as.data.frame()
    reaction_dat[reaction_dat$X1 == "R00005:", ]
    colnames(reaction_dat) <- c("rxn_ID", "pwy", "cpn_ID1", "dir", "cpn_ID2")
    reaction_dat$rxn_ID <- gsub(":", "", reaction_dat$rxn_ID)
    reaction_dat$pwy <- gsub(":", "", reaction_dat$pwy)
    reaction_dat <- reaction_dat[, -2]
    reaction_dat <- unique(reaction_dat)
    head(reaction_dat)
    dim(reaction_dat)

    reaction_ko <- readr::read_delim("DATA/EXTERNAL/reaction_ko.list", "\t", col_names = FALSE) %>% as.data.frame()
    colnames(reaction_ko) <- c("rxn_ID", "ko_ID")
    reaction_ko[] <- lapply(reaction_ko, function(x) gsub(".+:", "", x))
    head(reaction_ko)
    reaction_dat <- dplyr::left_join(reaction_dat, reaction_ko, by = c("rxn_ID" = "rxn_ID"))
    head(reaction_dat)

    # read mouse gene mapping
    gene_dat <- readr::read_delim("../DATA/EXTERNAL/T01002.kff", "\t", col_names = FALSE) %>% as.data.frame()
    head(gene_dat)
    reaction_dat$gene_Mm <- gene_dat[match(reaction_dat$ko_ID, gene_dat$X10), "X8"]
    reaction_dat <- reaction_dat[!is.na(reaction_dat$gene_Mm), ]
    dim(reaction_dat)
```

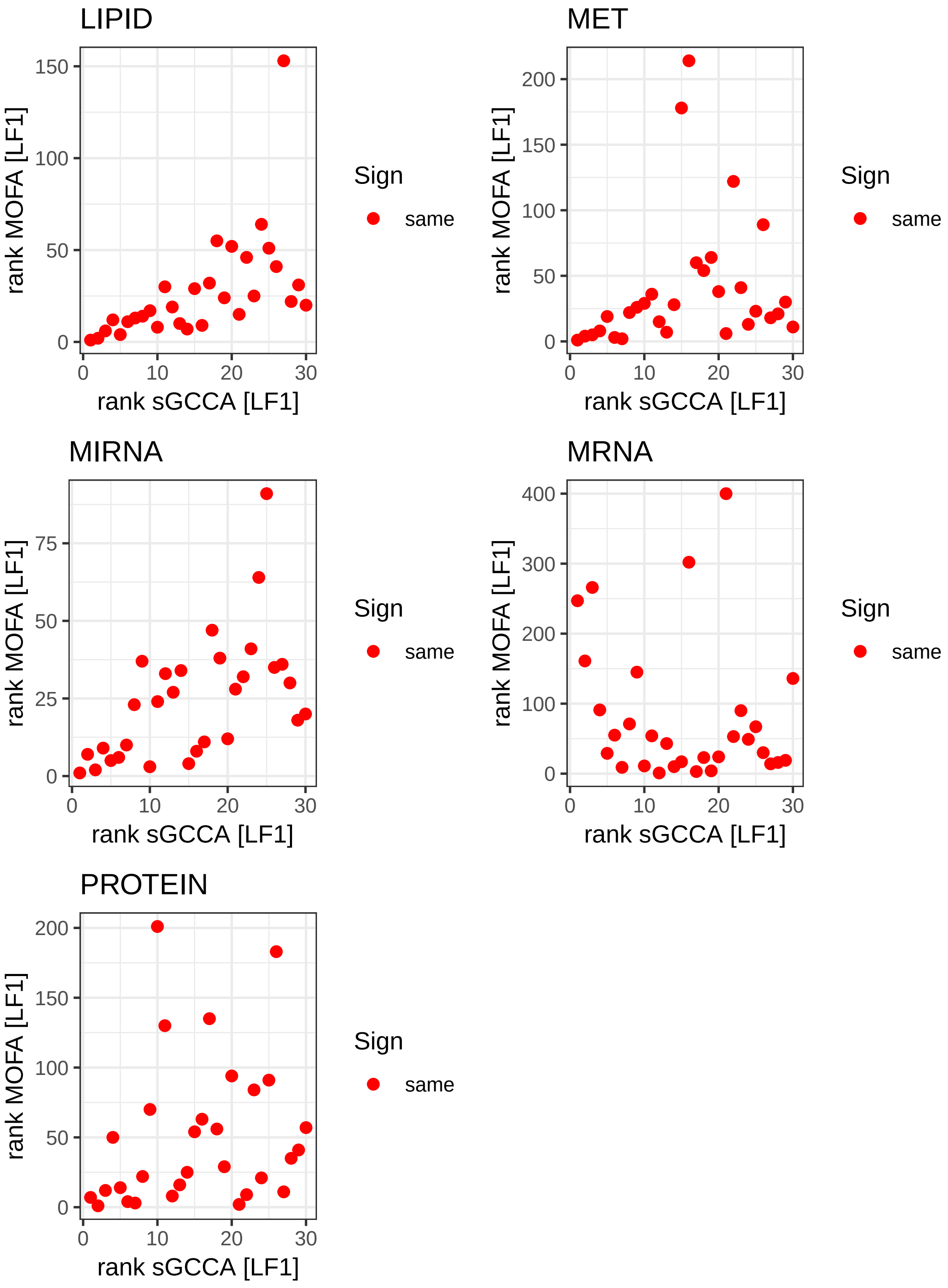

Figure 18: Comparison of loadings between MOFA and sGCCA approach.

```r
# read compound data mapping
compound_dat <- readr::read_delim("../DATA/EXTERNAL/cmp_extracted.table.txt", "\t", col_names = FALSE) %>%
    as.data.frame()
colnames(compound_dat) <- c("cpn_ID", "names")
compound_dat$label <- getsplit(compound_dat$names, ";", 1)
reaction_dat$cpn_lab1 <- compound_dat[match(reaction_dat$cpn_ID1, compound_dat$cpn_ID), "label"]
reaction_dat$cpn_lab2 <- compound_dat[match(reaction_dat$cpn_ID2, compound_dat$cpn_ID), "label"]
head(reaction_dat)

# graph with gene and metabolite nodes
g1_tab <- reaction_dat[, c("cpn_ID1", "gene_Mm")]
g2_tab <- reaction_dat[, c("cpn_ID2", "gene_Mm")]
colnames(g1_tab) <- colnames(g2_tab) <- c("cpn_ID", "gene_Mm")
g_tab <- rbind(g1_tab, g2_tab)
g_tab <- unique(g_tab)
g_met <- igraph::graph.data.frame(g_tab, directed = FALSE, vertices = NULL)

#  * Sub-Section * Create StringDB network
# ~~~~~~~~~~~~~~~~~~~~~~~~~~~~~~~~~~~~~~~~~~~~~~~~~~~~~~~~~~~~~~~~~~~~~~~~
string_mapping <- readr::read_tsv(file.path("../","DATA", "EXTERNAL", "10090.protein.aliases.v10.5.txt"),
  col_types = "ccc", col_names = c("STRINGID", "ID", "SOURCE"),
  skip = 1
) %>% as.data.frame()
dim(string_mapping)
head(string_mapping)

sel <- grep("Ensembl_EntrezGene", string_mapping$SOURCE, fixed = TRUE)
notsel <- grep("Ensembl_EntrezGene_synonym", string_mapping$SOURCE, fixed = TRUE)
sel <- setdiff(sel, notsel)
string_mapping <- string_mapping[sel, ]

string_net <- readr::read_delim(file.path("../","DATA", "EXTERNAL", "10090.protein.links.detailed.v10.5.txt"), " ",
  col_types = "cciiiiiiii",
  col_names = c("protein1", "protein2", "neighborhood",
                "fusion", "cooccurence", "coexpression",
                "experimental", "database", "textmining", "combined_score"),
  skip = 1
) %>% as.data.frame()
dim(string_net)
head(string_net)
string_net <- string_net[string_net$combined_score > 700, ] # high confidence defined by StringDB
dim(string_net)
string_net$Symbol1 <- string_mapping[match(string_net$protein1, string_mapping$STRINGID), "ID"]
string_net$Symbol2 <- string_mapping[match(string_net$protein2, string_mapping$STRINGID), "ID"]
string_net <- string_net[!is.na(string_net$Symbol1) & !is.na(string_net$Symbol2), ]
string_net <- string_net[, c(11, 12, 10, 1:9)]
dim(string_net)
head(string_net)
# make unique
KEY <- apply(string_net, 1, function(x) {
  ifelse(x["Symbol1"] < x["Symbol2"],
    paste0(x["Symbol1"], "-", x["Symbol2"]),
    paste0(x["Symbol2"], "-", x["Symbol1"])
  )
})
string_net <- string_net[!duplicated(KEY), ]
dim(string_net)

g_string <- igraph::graph.data.frame(string_net, directed = FALSE, vertices = NULL)
g_string

#  * Sub-Section * Prepare miRNA network
# ~~~~~~~~~~~~~~~~~~~~~~~~~~~~~~~~~~~~~~~~~~~~~~~~~~~~~~~~~~~~~~~~~~~~~~~~
# miRTarBase
# http://mirtarbase.mbc.nctu.edu.tw/php/download.php
dat <- read.xlsx2(file.path("../","DATA", "EXTERNAL", "mirTarBase_Mm_10July2018.xls"), sheetIndex = 1)

dat <- dat %>%
  group_by(miRTarBase.ID, miRNA, Target.Gene) %>%
  summarize(
    Experiments = paste0(Experiments, collapse = "//"),
    no_refs = length(unique(References..PMID.))
  )
dat <- as.data.frame(dat)
toMatch <- toupper(c("Western blot", "Reporter assay", "qRT-PCR"))
dat$no_hc <- stri_count_regex(toupper(dat$Experiments), paste(toMatch, collapse = "|"))
head(dat)
dat <- dat %>% dplyr::filter(no_refs > 1, no_hc > 1) %>% as.data.frame()
dat <- dat[, c(2, 3, 1, 4:6)]
```

```r
    g_mirna <- igraph::graph.data.frame(dat, directed = FALSE, vertices = NULL)
    g_mirna

    #  * Sub-Section *  Define weights and combine
    # ~~~~~~~~~~~~~~~~~~~~~~~~~~~~~~~~~~~~~~~~~~~~~~~~~~~~~~~~~~~~~~~~~~~~~~~~
    # check naming alignment
    V(g_met)$name[V(g_met)$name == "Acod1"] <- "Irg1"

    # define weights
    E(g_met)$weight <- 0.1
    E(g_string)$weight <- 1 - (E(g_string)$combined_score / 1000) + 0.1
    E(g_mirna)$weight <- 0.1

    g_comb <- igraph::union(g_met, g_string, g_mirna, byname = TRUE)
    class(g_comb)
    g_comb

    E(g_comb)$weight <- ifelse(is.na(E(g_comb)$weight_1), E(g_comb)$weight_2, E(g_comb)$weight_1)
    E(g_comb)$weight <- ifelse(is.na(E(g_comb)$weight_3), E(g_comb)$weight, E(g_comb)$weight_3)

    summary(E(g_comb)$weight)

    file <- file.path(reportDir, "P15038_LungManuscript_IntegrNetwork.rda")
    save(g_comb, file = file)
} else {
    if (force_rerun_pcsf) {
        file <- file.path(reportDir, "P15038_LungManuscript_IntegrNetwork.rda")
        if (!file.exists(file)) {
            stop("Need integrated network to rerun PCSF (required KEGG license to create/share)")
        }
        g_comb <- load2(file)
    } else {
      cat("Integrated network not needed (PCSF not rerun)\n")
    }
     compound_dat <- readr::read_delim("../DATA/EXTERNAL/cmp_extracted.table.txt", "\t", col_names = FALSE) %>%
            as.data.frame()
        colnames(compound_dat) <- c("cpn_ID", "names")
        compound_dat$label <- getsplit(compound_dat$names, ";", 1)
}
```

```
## Loading objects:
##   g_comb
```

14.2 PCSF-derived network

```r
max_terminal_view <- 200
res <- lapply(names(weights_mofa), function(view) {
  lval <- abs(weights_mofa[[view]][, "LF1"])
  lval <- lval / sum(lval)
  sel <- which(lval > 2 * 1 / length(lval))
  if (length(sel) > max_terminal_view) {
    return(lval[sel][order(lval[sel], decreasing = TRUE)[seq_len(max_terminal_view)]])
  } else {
    return(lval[sel])
  }
})
names(res) <- names(weights_mofa)
lapply(res, length)
```

```
## $LIPID
## [1] 43
##
## $MET
## [1] 82
##
## $MIRNA
## [1] 55
##
## $MRNA
## [1] 200
##
## $PROTEIN
## [1] 169
```

```r
# fix names
Imet <- dats[["METABOLITE"]]$I_metabolite
names(res[["MET"]]) <- Imet[match(names(res[["MET"]]), Imet$BIOCHEMICAL), "KEGG"]
```

```
nm <- names(res[["MET"]])
res[["MET"]] <- res[["MET"]][nm != "" & !is.na(nm)]

names(res[["PROTEIN"]]) <- getsplit(names(res[["PROTEIN"]]), "_", 1)
nm <- names(res[["PROTEIN"]])
res[["PROTEIN"]] <- res[["PROTEIN"]][nm != "" & !is.na(nm)]

lipid2kegg <- read.csv("../DATA/P15038_LungManuscript_LipidMappingTable3_bt.csv")
names(res[["LIPID"]]) <- gsub(" ", "", gsub("\xa0", "", lipid2kegg[match(names(res[["LIPID"]]),
                                                                          lipid2kegg$Name_PMI), "KEGG"]))
nm <- names(res[["LIPID"]])
res[["LIPID"]] <- res[["LIPID"]][nm != "" & !is.na(nm)]
res[["LIPID"]] <- tapply(res[["LIPID"]], names(res[["LIPID"]]), mean, na.rm = TRUE)

terminals <- c(res[["PROTEIN"]], res[["MRNA"]], res[["MET"]], res[["MIRNA"]], res[["LIPID"]])
terminals <- tapply(unname(terminals), names(terminals), function(x) max(x))

set.seed(4823972)
test_grid <- expand.grid(omega = seq(0.20, 1, by = 0.2), beta = c(100, 200, 400, 1000, 2000),
                         stringsAsFactors = FALSE)
test_grid$label <- paste0(sprintf("%02d", 1:nrow(test_grid)), ":w=",
                          test_grid[, "omega"], ",b=", test_grid[, "beta"])

file <- file.path(reportDir, "P15038_LungManuscript_Network_PCSF.rda")
if (!force_rerun_pcsf && file.exists(file)) {
    pcsf.res <- load2(file)
} else {
    pcsf.res <- lapply(1:nrow(test_grid), function(i) {
      cat(i, "\n")
      subnet <- PCSF(g_comb,
        terminals,
        w = test_grid[i, "omega"],
        b = test_grid[i, "beta"],
        mu = 0.0005
      )
      return(subnet)
    })
    save(pcsf.res, file = file)
}
```

```
## 1
##   75.4% of your terminal nodes are included in the interactome
##   Solving the PCSF...
## 2
##   75.4% of your terminal nodes are included in the interactome
##   Solving the PCSF...
## 3
##   75.4% of your terminal nodes are included in the interactome
##   Solving the PCSF...
## 4
##   75.4% of your terminal nodes are included in the interactome
##   Solving the PCSF...
## 5
##   75.4% of your terminal nodes are included in the interactome
##   Solving the PCSF...
## 6
##   75.4% of your terminal nodes are included in the interactome
##   Solving the PCSF...
## 7
##   75.4% of your terminal nodes are included in the interactome
##   Solving the PCSF...
## 8
##   75.4% of your terminal nodes are included in the interactome
##   Solving the PCSF...
## 9
##   75.4% of your terminal nodes are included in the interactome
##   Solving the PCSF...
## 10
##   75.4% of your terminal nodes are included in the interactome
##   Solving the PCSF...
## 11
##   75.4% of your terminal nodes are included in the interactome
##   Solving the PCSF...
## 12
##   75.4% of your terminal nodes are included in the interactome
##   Solving the PCSF...
## 13
##   75.4% of your terminal nodes are included in the interactome
##   Solving the PCSF...
## 14
```

```
##   75.4% of your terminal nodes are included in the interactome
##   Solving the PCSF...
## 15
##   75.4% of your terminal nodes are included in the interactome
##   Solving the PCSF...
## 16
##   75.4% of your terminal nodes are included in the interactome
##   Solving the PCSF...
## 17
##   75.4% of your terminal nodes are included in the interactome
##   Solving the PCSF...
## 18
##   75.4% of your terminal nodes are included in the interactome
##   Solving the PCSF...
## 19
##   75.4% of your terminal nodes are included in the interactome
##   Solving the PCSF...
## 20
##   75.4% of your terminal nodes are included in the interactome
##   Solving the PCSF...
## 21
##   75.4% of your terminal nodes are included in the interactome
##   Solving the PCSF...
## 22
##   75.4% of your terminal nodes are included in the interactome
##   Solving the PCSF...
## 23
##   75.4% of your terminal nodes are included in the interactome
##   Solving the PCSF...
## 24
##   75.4% of your terminal nodes are included in the interactome
##   Solving the PCSF...
## 25
##   75.4% of your terminal nodes are included in the interactome
##   Solving the PCSF...
```

```
stat <- unlist(lapply(pcsf.res, function(g) {
  comps <- components(g)
  comps$no
}))
dat <- data.frame(no_trees = stat)
dat <- cbind(test_grid, dat)
p <- ggplot(aes(x = label, y = no_trees, fill = beta, colour = omega), data = dat)
p <- p + geom_bar(stat = "identity")
p <- p + scale_fill_distiller(type = "seq", palette = "Purples", direction = 1)
p <- p + scale_colour_distiller(type = "seq", palette = "Greens", direction = 1)
p <- p + theme_bw()
p <- p + labs(x = "", y = "#trees")
p <- p + theme(axis.text.x = element_text(angle = 90, hjust = 1, vjust = 0.5))
p1 <- p

stat <- unlist(lapply(pcsf.res, function(g) {
  sum(names(terminals) %in% V(g)$name) / length(terminals) * 100
}))
dat <- data.frame(terminals_percent = stat)
dat <- cbind(test_grid, dat)
p <- ggplot(aes(x = label, y = terminals_percent, fill = beta, colour = omega), data = dat)
p <- p + geom_bar(stat = "identity")
p <- p + scale_fill_distiller(type = "seq", palette = "Purples", direction = 1)
p <- p + scale_colour_distiller(type = "seq", palette = "Greens", direction = 1)
p <- p + theme_bw()
p <- p + labs(x = "", y = "terminals %")
p <- p + theme(axis.text.x = element_text(angle = 90, hjust = 1, vjust = 0.5))
p2 <- p

stat_dat <- do.call(rbind, lapply(1:length(pcsf.res), function(i) {
  g <- pcsf.res[[i]]
  comps <- components(g)
  data.frame(testrun = as.character(i), comp = as.character(1:length(comps$csize)), no_elements = comps$csize)
}))
test_grid2 <- test_grid
test_grid2$testrun <- as.character(rownames(test_grid2))
stat_dat <- left_join(stat_dat, test_grid2, by = "testrun")

p <- ggplot(aes(x = label, y = no_elements, fill = comp), data = stat_dat)
p <- p + geom_bar(stat = "identity", colour = "black")
# p = p + scale_fill_distiller(type = "seq", palette = "Purples", direction = 1)
# p = p + scale_colour_distiller(type = "seq", palette = "Greens", direction = 1)
p <- p + theme_bw()
p <- p + labs(x = "", y = "nodes per component")
p <- p + theme(axis.text.x = element_text(angle = 90, hjust = 1, vjust = 0.5))
```

```r
p3 <- p

file <- file.path(reportDir, "P15038_LungManuscript_Network_PCSF_stats.png")
png(file = file, units = "in", res = 200, width = 7, height = 14)
gridExtra::grid.arrange(p1, p2, p3, ncol = 1)
dev.off()
```

```
## pdf
##   2
```

```r
# SELECT PARAMS
# 18: w = 0.6; b = 1000
# rational:
#   - low number but not only single tree
#   - good coverage of terminals (close to saturation)

# run randomized version
seltest <- 18
set.seed(423987823)

file <- file.path(reportDir, "P15038_LungManuscript_Network_PCSF_final.rda")
if (!force_rerun_pcsf && file.exists(file)) {
    subnet_final <- load2(file)
} else {
    subnet_final <- PCSF_rand(g_comb, terminals, n = 10, r = 0.1, w = test_grid[seltest, "omega"],
                              b = test_grid[seltest, "beta"], mu = 0.0005)
    save(subnet_final, file = file)
}
```

```
##   75.4% of your terminal nodes are included in the interactome
##   Solving the PCSF by adding random noise to the edge costs...
```

```r
subnet_final2 <- subnet_final
sel <- grepl("^C\\d{5}", V(subnet_final2)$name)
V(subnet_final2)[sel]$name <- compound_dat[match(V(subnet_final2)[sel]$name, compound_dat$cpn_ID), "label"]

res_network_enrich <- enrichment_analysis(subnet_final2)
```

```
##   Performing enrichment analysis...
##
##   Enrichment is being performed by EnrichR (http://amp.pharm.mssm.edu/Enrichr) API ...
```

```r
file <- file.path(reportDir, "P15038_LungManuscript_PCSF_network_final_enrich.rda")
save(res_network_enrich, file = file)
```

Figure 19 shows the grid-search results for PCSF-based network identification. Based on these results, we selected condition #18 (w = 0.6; b = 1000) for functional interpretation of the multi-omics links. The rational for the selection was to end up with a low number of trees (but not only a single tree) and a good coverage of terminals (close to saturation).

```r
# alternative visualization
subnet <- res_network_enrich$subnet
edge_width <- 8
node_size <- 30
node_label_cex <- 1
Terminal_node_legend <- "Terminal"
Steiner_node_legend <- "Steiner"

V(subnet)$label.cex <- node_label_cex

# prize = abs(V(subnet)$prize)
# min1 = 10
# max1 = node_size
# r1 = max1 - min1
# min2 = min(prize)
# max2 = max(prize)
# r2 = max2 - min2
# adjusted_prize = r1 * (prize - min2)/r2 + min1
# V(subnet)$size = adjusted_prize
V(subnet)$size <- node_size

weight <- E(subnet)$weight
min1 <- 2
max1 <- edge_width
r1 <- max1 - min1
min2 <- min(weight)
max2 <- max(weight)
r2 <- max2 - min2
adjusted_weight <- r1 * (weight - min2) / r2 + min1
E(subnet)$width <- adjusted_weight
```

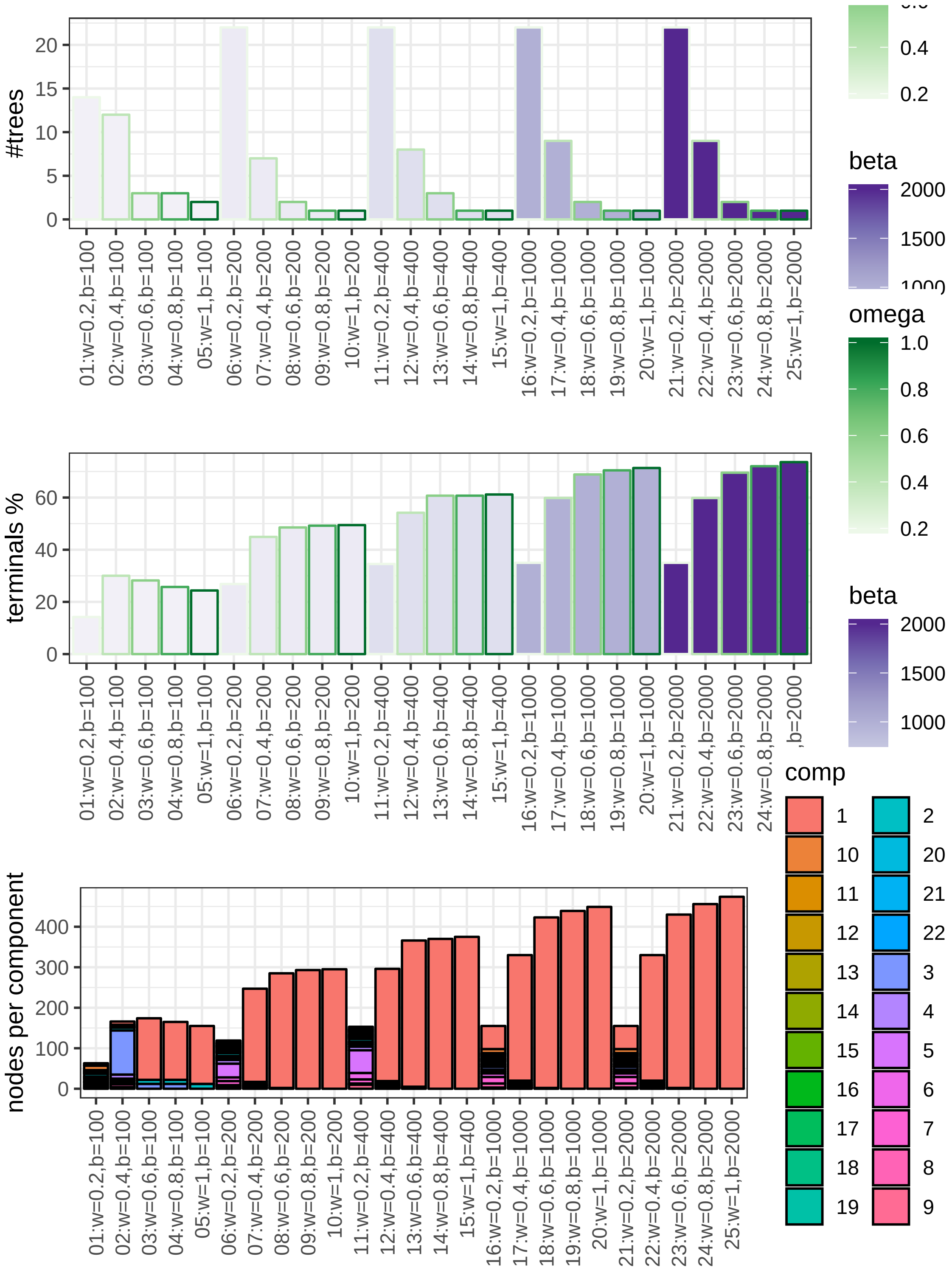

Figure 19: Grid-search results for PCSF-based network identification.

```r
V(subnet)$borderWidth <- 1
V(subnet)$borderWidth <- ifelse(V(subnet)$type == "Steiner", 1, 3)

V(subnet)$mol_type <- "gene/protein"
V(subnet)$mol_type[grepl("-miR-", V(subnet)$name)] <- "miRNA"
V(subnet)$mol_type[V(subnet)$name %in% compound_dat$label] <- "compound"

# shape = V(subnet)$type
# shape[which(shape == "Steiner")] = "triangle"
# shape[which(shape == "Terminal")] = "square"
V(subnet)$shape <- c(
  "gene/protein" = "square",
  "miRNA" = "triangle",
  "compound" = "dot"
)[V(subnet)$mol_type]

# create graph plot
gvp <- visIgraph(subnet) %>%
  visIgraphLayout(
    layout = "cluster_layout", # "cluster_layout", #layout_with_fr
    randomSeed = 47839,
    sel_weights = c(0.5, 0.05)
  ) %>%
  visOptions(highlightNearest = list(enabled = TRUE), selectedBy = "group") %>%
  visLegend(
    addNodes = list(
      list(
        label = "gene/protein",
        shape = "square",
        size = 10,
        label.cex = 0.3
      ),
      list(
        label = "miRNA",
        shape = "triangle",
        size = 10,
        label.cex = 0.3
      ),
      list(
        label = "compound",
        shape = "dot",
        size = 10,
        label.cex = 0.3
      ),
      list(
        label = "terminal",
        shape = "square",
        size = 10,
        borderWidth = 3,
        label.cex = 0.3
      )
    ),
    width = 0.2, useGroups = FALSE
  ) %>%
  visNodes(scaling = list(label = list(enabled = FALSE)))

# save graph plot
file <- file.path(getwd(),"../","REPORT", "P15038_LungManuscript_PCSF_network.html")
gvp %>% visSave(file = file)

file2 <- file.path(reportDir, "P15038_LungManuscript_PCSF_network.rda")
save(gvp, file = file2)

# load(file2)
# file3 = file.path(reportDir, "P15038_LungManuscript_PCSF_network.pdf")
# gvp %>% visExport(type = "png", name = "export-network",
#                    float = "left", label = "Save network", background = "white", style= "")
# poor resolution

# plot directly with igraph
subnet2 <- subnet
V(subnet2)$shape <- c(
  "square" = "square",
  "triangle" = "csquare",
  "dot" = "circle"
)[V(subnet2)$shape]
V(subnet2)$size <- 5
V(subnet2)$label.font <- 1
V(subnet2)$label.font[V(subnet)$type == "Terminal"] <- 2
```

```r
V(subnet2)$edge.curved <- TRUE

set.seed(34234)
layout <- cluster_layout(subnet2, sel_weights = c(0.5, 0.05))
layout[, 2] <- -layout[, 2]

grp_cols <- colorRampPalette(brewer.pal(9, "Set2"))(length(unique(V(subnet2)$group)))
names(grp_cols) <- unique(V(subnet2)$group)
V(subnet2)$color <- grp_cols[V(subnet2)$group]

file <- file.path(reportDir, "P15038_LungManuscript_PCSF_network.pdf")
pdf(file = file, width = 17, height = 15)
plot.igraph(subnet2, layout = layout)
legend("right", legend = names(grp_cols), fill = grp_cols, title = "Groups", horiz = FALSE)
dev.off()
```

```
## pdf
##   2
```

```r
# Only show labels for examples in main figure
subnet3 <- subnet2
sel_molecules_0 <- c("Irg1",
                   "Blvrb",
                   "mmu-miR-34a-5p",
                   "Pla2g2d",
                   "Sat1")
sel_molecules <-  names(unlist(ego(subnet3, nodes = sel_molecules_0)))

sel <- which(V(subnet3)$name %in% sel_molecules)
V(subnet3)$name[-sel] <- ""

file <- file.path(reportDir, "P15038_LungManuscript_PCSF_network_example_sel_labels.pdf")
pdf(file = file, width = 17, height = 15)
plot.igraph(subnet3, layout = layout, vertex.label.cex= 2)
legend("right", legend = names(grp_cols), fill = grp_cols, title = "Groups", horiz = FALSE)
dev.off()
```

```
## pdf
##   2
```

```r
# plot select neighborhoods
for (sel_mol in sel_molecules_0) {
  sel <-  unlist(ego(subnet2, nodes = sel_mol, order = 2))
  sg <- induced_subgraph(subnet2, sel)

  set.seed(34234)
  layout2 <- cluster_layout(sg, sel_weights = c(0.5, 0.05))
  layout2[, 2] <- -layout2[, 2]

  file <- file.path(reportDir, paste0("P15038_LungManuscript_PCSF_network_mol_", sel_mol, ".pdf"))
  pdf(file = file, width = 5, height = 5)
  plot.igraph(induced_subgraph(subnet2, sel) , layout = layout2)
  # legend("right",legend=names(grp_cols),fill=grp_cols, title = "Groups", horiz = FALSE)
  dev.off()
}

# also plot full clusters
for (gr in unique(V(subnet2)$group)) {
  sel <- which(V(subnet2)$group == gr)
  file <- file.path(reportDir, paste0("P15038_LungManuscript_PCSF_network_cl", gr, ".pdf"))
  pdf(file = file, width = 17, height = 15)
  plot.igraph(induced_subgraph(subnet2, sel) , layout = layout[sel,])
  # legend("right",legend=names(grp_cols),fill=grp_cols, title = "Groups", horiz = FALSE)
  dev.off()
}
```

Figure 20 shows the derived multi-omics response network.

14.3 GSEA for functional interpretation of clusters

```r
# cluster annotation using reactome db
gsc_file <- readLines("../DATA/EXTERNAL/ReactomePathways_23Apr2018.gmt")
gsc <- do.call(rbind, lapply(gsc_file, function(x) {
  ss <- strsplit(x, "\t", fixed = TRUE)[[1]]
  genes <- ss[seq(3, length(ss))]
  gs <- ss[1]
  data.frame(Gene = genes, GS = gs)
```

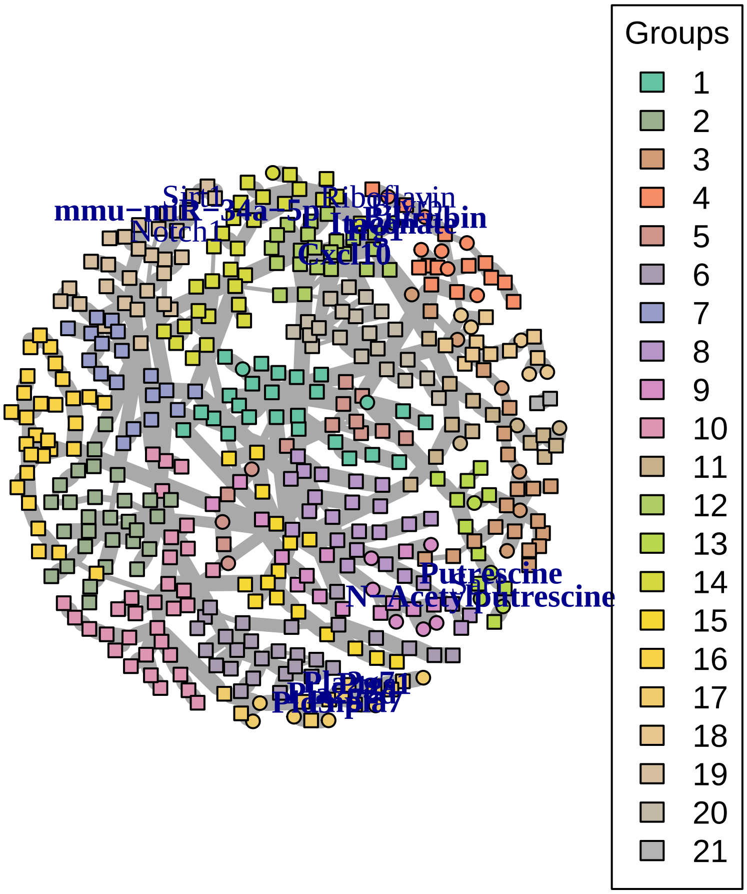

Figure 20: Aggregated gene–protein–metabolite–miRNA network for LF 1 constructed using the PCSF algorithm.

```r
}))
gsc <- piano::loadGSC(gsc)

global_universe <- unique(unlist(lapply(gsc$gsc, function(x) x)))
subnet <- res_network_enrich$subnet
grp <- V(subnet)$group
enrich_res <- lapply(unique(grp), function(gr) {
  genes <- GetOrtholog(V(subnet)[V(subnet)$group == gr]$name, species_from = "Mm", species_to = "Hs")
  genes <- unique(genes[!is.na(genes)])
  if (length(genes) > 5) {
        set.seed(209438)
        res_tab <- piano::runGSAhyper(genes, universe = global_universe, gsc = gsc, gsSizeLim = c(5, Inf))$resTab
  } else {
    res_tab <- NULL
  }
  return(res_tab)
})
```

```
## Loading objects:
##   HCOP_ORTHOLOGY_TABLE
## Loading objects:
##   HCOP_ORTHOLOGY_TABLE
## Loading objects:
##   HCOP_ORTHOLOGY_TABLE
## Loading objects:
##   HCOP_ORTHOLOGY_TABLE
## Loading objects:
##   HCOP_ORTHOLOGY_TABLE
## Loading objects:
##   HCOP_ORTHOLOGY_TABLE
## Loading objects:
##   HCOP_ORTHOLOGY_TABLE
## Loading objects:
##   HCOP_ORTHOLOGY_TABLE
## Loading objects:
##   HCOP_ORTHOLOGY_TABLE
## Loading objects:
##   HCOP_ORTHOLOGY_TABLE
## Loading objects:
##   HCOP_ORTHOLOGY_TABLE
## Loading objects:
##   HCOP_ORTHOLOGY_TABLE
## Loading objects:
##   HCOP_ORTHOLOGY_TABLE
## Loading objects:
##   HCOP_ORTHOLOGY_TABLE
## Loading objects:
##   HCOP_ORTHOLOGY_TABLE
## Loading objects:
##   HCOP_ORTHOLOGY_TABLE
## Loading objects:
##   HCOP_ORTHOLOGY_TABLE
## Loading objects:
##   HCOP_ORTHOLOGY_TABLE
## Loading objects:
##   HCOP_ORTHOLOGY_TABLE
## Loading objects:
##   HCOP_ORTHOLOGY_TABLE
## Loading objects:
##   HCOP_ORTHOLOGY_TABLE
```

```r
names(enrich_res) <- unique(grp)
file <- file.path(reportDir, "P15038_LungManuscript_PCSF_network_final_ORA_clusters.rda")
save(enrich_res, file = file)

enrich_res <- lapply(enrich_res, function(er) er[er[, "Adjusted p-value"] < 0.05, , drop = FALSE])
# enrich_res <- enrich_res[!sapply(enrich_res, is.null)]

enrich_res_sum <- do.call(rbind, lapply(unique(V(subnet)$group), function(gr) {
  nodes <- paste0(V(subnet)[V(subnet)$group == gr]$name, collapse = ";")
  er <- enrich_res[[gr]]
  if (!is.null(er) && nrow(er) > 0) {
    er <- er[order(er[, "Adjusted p-value"], decreasing = FALSE), , drop = FALSE]
  }
  er <- paste0(rownames(er), " (",
    sprintf("%.2E", er[, "Adjusted p-value"]),
    ")",
    collapse = "|"
  )
  er <- gsub("()", "", er, fixed = TRUE)
  data.frame(Cluster = gr, Nodes = nodes, GeneSets = er)
```

```
}))
file <- file.path(reportDir, "P15038_LungManuscript_PCSF_network_final_CLUSTER_TABLE.csv")
write.csv(enrich_res_sum, file = file)

# Cluster annotation based on enrichment results:
cluster_annotation_table <- matrix(c("Cl01", "Lipid metabolism",
                                     "Cl02", "",
                                     "Cl03", "",
                                     "Cl04", "Xenobiotic metabolism",
                                     "Cl05", "Xenobiotic metabolism",
                                     "Cl06", "",
                                     "Cl07", "Immune-related",
                                     "Cl08", "Immune-related",
                                     "Cl09", "Nucleotide metabolism",
                                     "Cl10", "Immune-related",
                                     "Cl11", "Carbohydrate metabolism",
                                     "Cl12", "Immune-related",
                                     "Cl13", "Polyamine metabolism",
                                     "Cl14", "Immune-related",
                                     "Cl15", "Oxidative-stress response",
                                     "Cl16", "",
                                     "Cl17", "Lipid metabolism",
                                     "Cl18", "Lipid metabolism",
                                     "Cl19", "Immune-related",
                                     "Cl20", "Extracellular matrix",
                                     "Cl21", ""),
                                   ncol = 2, byrow = TRUE)
cluster_annotation_table <- as.data.frame(cluster_annotation_table)
colnames(cluster_annotation_table) <- c("Cluster", "General category")

file <- file.path(reportDir, "P15038_LungManuscript_PCSF_network_final_CLUSTER_ANNOT_TABLE.csv")
write.csv(cluster_annotation_table, file = file)
```

Table 4: General functional annotations for clusters (based on ORA results). Table **??** lists the proposed cluster annotations based on the ORA results.

Cluster	General category
Cl01	Lipid metabolism
Cl02	
Cl03	
Cl04	Xenobiotic metabolism
Cl05	Xenobiotic metabolism
Cl06	
Cl07	Immune-related
Cl08	Immune-related
Cl09	Nucleotide metabolism
Cl10	Immune-related
Cl11	Carbohydrate metabolism
Cl12	Immune-related
Cl13	Polyamine metabolism
Cl14	Immune-related
Cl15	Oxidative-stress response
Cl16	
Cl17	Lipid metabolism
Cl18	Lipid metabolism
Cl19	Immune-related
Cl20	Extracellular matrix
Cl21	

14.4 Response profiles

```r
#  * Sub-Section * Response profiles for clusters
# ~~~~~~~~~~~~~~~~~~~~~~~~~~~~~~~~~~~~~~~~~~~~~~~~~~~~~~~~~~~~~~~~~~~~~~~~~~~
subnet <- res_network_enrich$subnet
subnet_final2 <- subnet_final
V(subnet_final2)$group <- V(subnet)$group

Imet <- dats[["METABOLITE"]]$I_metabolite

grps <- V(subnet_final2)$group
names(grps) <- V(subnet_final2)$name
grps <- sort(grps)

# align

# start with combined heatmaps
# lapply(idmaps, function(x) names(x))
all_contrasts <- names(idmaps[["MRNA"]])
X_list <- lapply(names(idmaps), function(x) {
  apm <- getIDMAPentry(idmaps[[x]], entry = "adj.p.value")
  fcm <- getIDMAPentry(idmaps[[x]], entry = "foldChange")

  rn <- rownames(apm)
  if (x == "PROTEIN") {
    rn <- getsplit(rn, "_", 1)
  } else if (x == "METABOLITE") {
    rn <- Imet[match(rn, Imet$BIOCHEMICAL), "KEGG"]
  }
  sel <- which(!is.na(rn) & rn != "")

  # go by sum effect for collapsing
  sum_fc <- apply(fcm, 1, sum, na.rm = TRUE)
  sel <- unlist(tapply(sel, rn[sel], function(y) {
    if (length(y) == 1) {
      return(y)
    } else {
      return(sel[which.max(sum_fc[sel])])
    }
  }))
  apm <- apm[sel, match(all_contrasts, colnames(apm))]
  fcm <- fcm[sel, match(all_contrasts, colnames(fcm))]
  rownames(apm) <- rownames(fcm) <- rn[sel]
  colnames(apm) <- colnames(fcm) <- all_contrasts

  return(list(apm = apm, fcm = fcm))
})
names(X_list) <- names(idmaps)

res_list <- list()
for (gr in unique(grps)) {
  sel_mol <- names(grps)[grps == gr]
  label_lookup <- c(
    "MRNA" = "mRNA",
    "METABOLITE" = "met",
    "PROTEIN" = "prot",
    "MIRNA" = "miR",
    "LIPID" = "lip"
  )
  X_list_sel <- lapply(names(X_list), function(x) {
    rn <- rownames(X_list[[x]]$apm)
    apm <- X_list[[x]]$apm[rn %in% sel_mol, , drop = FALSE]
    fcm <- X_list[[x]]$fcm[rn %in% sel_mol, , drop = FALSE]
    if (nrow(apm) > 0) {
      rn <- rownames(fcm)
      if (x == "METABOLITE") {
        rn <- Imet[match(rn, Imet$KEGG), "BIOCHEMICAL"]
      }
      rownames(apm) <- rownames(fcm) <- paste0(label_lookup[x], "|", rn)
    }
    return(list(fcm = fcm, apm = apm))
  })
  apm <- do.call(rbind, lapply(X_list_sel, function(x) x$apm))
  sigm <- apm
  sigm[] <- NA
  sigm[apm < 0.05] <- 8
  fcm <- do.call(rbind, lapply(X_list_sel, function(x) x$fcm))

  file <- file.path(reportDir, paste0("P15038_LungManuscript_PCSF_Cluster_HM_", gr, ".pdf"))
  pdf(file = file, width = 6, height = 2 + 3 * (nrow(fcm) / 40))
  ImagePlotGG(fcm,
    textmat = sigm,
```

```r
    title = paste0("Cluster ", gr),
    useSymbols = TRUE,
    symbol_labels = c("fdr < 0.05", "NS")
  )
  dev.off()

  # create summary stats for cluster
  dat <- reshape2::melt(apm)
  colnames(dat) <- c("Analyte", "Contrast", "fdr")
  dat$ome <- getsplit(dat$Analyte, "|", 1)
  dat2 <- reshape2::melt(fcm)
  stopifnot(all((dat$Analyte == dat2$Var1)) && all(dat$Contrast == dat2$Var2))
  dat$fc <- dat2$value
  res <- dat %>%
    group_by(Contrast) %>%
    summarise(
      perc_signif = sum(fdr < 0.05, na.rm = TRUE) / n(),
      mean_effect = mean(fc, na.rm = TRUE)
    ) %>%
    as.data.frame()
  res$grp <- gr
  res_list[[gr]] <- res
}
res <- do.call(rbind, res_list)
res$Contrast <- factor(res$Contrast, levels = all_contrasts)
res$Cluster <- paste0("Cl", sprintf("%02d", res$grp))
res$Cluster <- factor(res$Cluster, levels = rev(unique(res$Cluster)))

p <- ggplot(aes(x = Contrast, y = Cluster), data = res)
p <- p + geom_tile(aes(fill = mean_effect))
col3 <- c(brewer.pal(n = 9, "Blues")[rev(c(2, 5, 9))], "white", brewer.pal(n = 9, "YlOrRd")[c(2, 5, 9)])

max_val <- 1.1 * max(abs(res$mean_effect), na.rm = TRUE)
values <- seq(-max_val, max_val, length = length(col3) + 1)
p <- p + scale_fill_gradientn(
  colours = col3, name = "",
  limits = c(-max_val, max_val), na.value = "grey60"
) +
  scale_x_discrete(expand = c(0, 0)) + scale_y_discrete(expand = c(0, 0))

p <- p + geom_point(aes(colour = perc_signif), size = 6)
p <- p + scale_colour_distiller(palette = "Greys", direction = 1)

p <- p + labs(x = "", y = "")
p <- p + theme(axis.text.x = element_text(angle = 90, hjust = 1, vjust = 0.5))
p <- p + theme(panel.border = element_rect(colour = "black", fill = NA, size = 1))
p <- p + geom_vline(xintercept = c(3, 6, 9, 11) + 0.5, colour = "black", size = 1)
p_PCSF_cluster_summary <- p

file <- file.path(reportDir, "P15038_LungManuscript_PCSF_Cluster_Summary.pdf")
pdf(file = file, width = 6, height = 8, useDingbats = FALSE)
print(p_PCSF_cluster_summary)
dev.off()
```

```
## pdf
##   2
```

```r
# also indicate functional categories
colnames(cluster_annotation_table) <- gsub(" ", "_", colnames(cluster_annotation_table))
cluster_annotation_table$Cluster <- factor(cluster_annotation_table$Cluster, levels = rev(cluster_annotation_table$Cluster))
cluster_annotation_table[cluster_annotation_table == ""] <- NA
p2 <- ggplot(aes(x = 1, y = Cluster, fill = General_category), data = cluster_annotation_table)
p2 <- p2 + geom_tile()
p2 <- p2 + scale_fill_brewer(type = "q", palette = "Set3")
p2 <- p2 + labs(x = "", y = "")
p2 <- p2 + theme_minimal()
p2 <- p2 + theme(panel.background = element_blank(),
               panel.grid = element_blank(),
               axis.text = element_blank())

file <- file.path(reportDir, "P15038_LungManuscript_PCSF_Cluster_Summary_Annot.pdf")
pdf(file = file, width = 10, height = 8, useDingbats = FALSE)
print(ggpubr::ggarrange(plotlist = list(p_PCSF_cluster_summary, p2),
          ncol = 2, widths = c(8,4), align = "h",
          common.legend = FALSE,
          legend = "right"))
dev.off()
```

```
## pdf
##   2
```

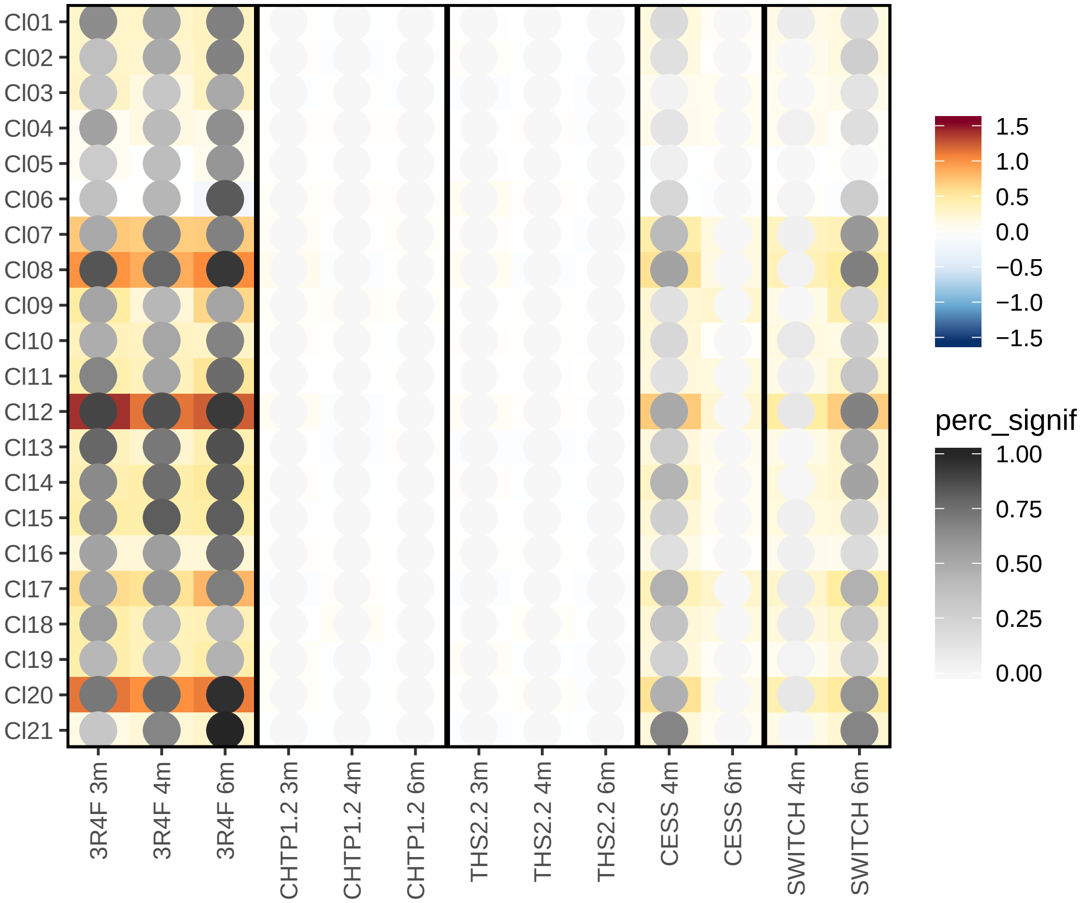

Figure 21: Expression profiles for identified clusters.

```
# also export data table
file <- file.path(reportDir, "P15038_LungManuscript_PCSF_Cluster_Summary.csv")
write.csv(res, file = file)
```

Figure 21 shows the response profiles of the identified clusters.

15 Metabolite and miRNA changes associated with 3R4F CS-induced immune response

15.1 Network enrichment analysis for the Macrophage Signaling network

```
nw_name <- "IPN / Macrophage Signaling"
sel_nw <- which(npa_list$networks() == nw_name)
npa <- subset(npa_list, sel_nw, NULL)

file <- file.path(reportDir, "P15038_LungManuscript_Macrophage_NPA.pdf")
pdf(file = file, width = 5, height = 6, useDingbats = FALSE)
barplot(npa,
        legend.text = FALSE,
        col = attr(idmaps[["MRNA"]], "colors"),
        bg = "white",
        main = nw_name)
dev.off()
```

```
## pdf
```

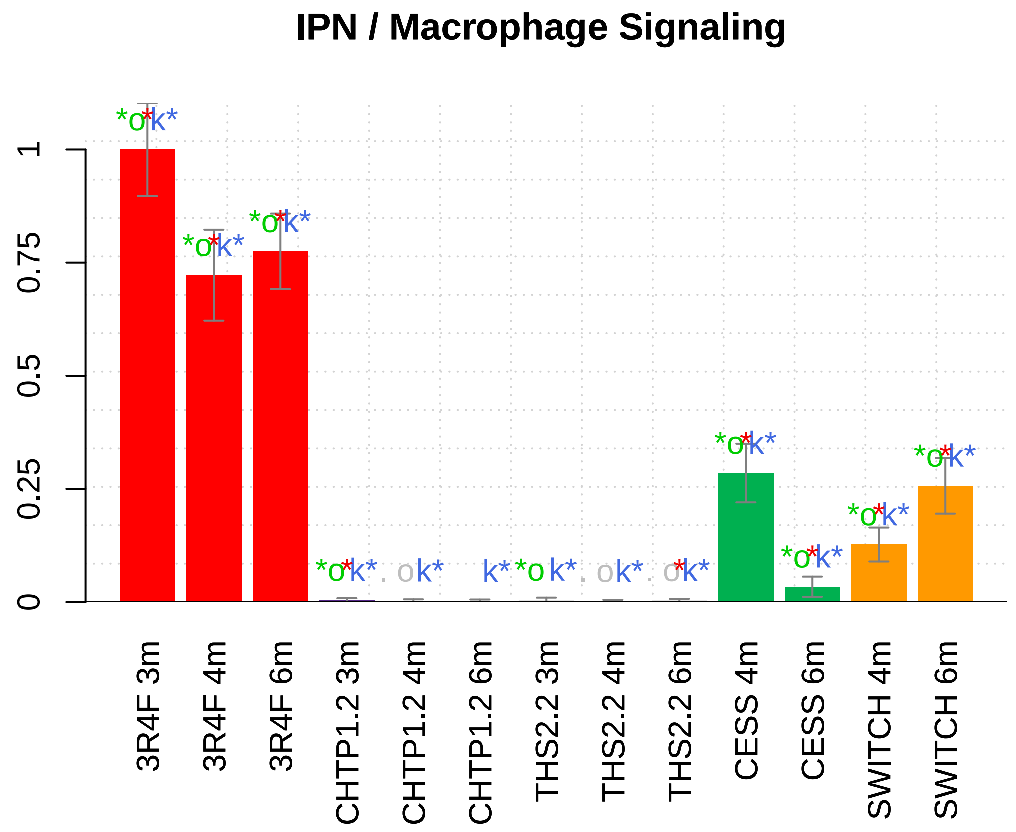

Figure 22: Network enrichment analysis for the Macrophage Signaling network.

```
## 2
```

Figure 22 shows the network enrichment analysis for the Macrophage Signaling network.

15.2 Itaconate metabolic pathway & correlations

```
#  * Sub-Section * Itaconate metabolic network
# ~~~~~~~~~~~~~~~~~~~~~~~~~~~~~~~~~~~~~~~~~~~~~~~~~~~~~~~~~~~~~~~~~~~~~~
col <- attr(idmaps[["MRNA"]], "colors")
names(col) <- names(idmaps[["MRNA"]])

sel_matrix <- matrix(c(
  "MRNA", "Irg1",
  "MRNA", "Aco1",
  "MRNA", "Aco2",
  "MRNA", "Idh1",
  "MRNA", "Idh2",
  "METABOLITE", "itaconate",
  "METABOLITE", "citrate",
  "METABOLITE", "alpha-ketoglutarate"
),
byrow = TRUE, ncol = 2
)

p_itaconate_pathway <- list()
for (i in 1:nrow(sel_matrix)) {
  idmap_sel <- idmaps[[sel_matrix[i, 1]]]
  gene_sel <- sel_matrix[i, 2]

  dat <- data.frame(
    fc = as.numeric(getFromIDMAP(idmap_sel, gene_sel, type = "foldChange", exact = TRUE)),
    apv = as.numeric(getFromIDMAP(idmap_sel, gene_sel, type = "adj.p.value", exact = TRUE)),
    contrasts = names(idmap_sel)
```

```
  )
  dat$sig <- ifelse(dat$apv < 0.05, "*", "")
  dat$contrasts <- factor(dat$contrasts, levels = dat$contrasts)

  p <- ggplot(aes(x = contrasts, y = fc, fill = contrasts), data = dat) +
    geom_bar(stat = "identity", width = 0.7) +
    scale_fill_manual("", values = col, guide = FALSE) +
    geom_text(aes(x = contrasts, y = fc + (max(abs(fc)) / 30 * sign(fc)), label = sig), data = dat, colour = "black") +
    labs(x = "", y = "log2 fold-change", title = gene_sel) +
    theme_bw() +
    theme(axis.text.x = element_text(angle = 90, hjust = 1, vjust = 0.5))
  p1 <- p +
    theme(
      axis.text = element_blank(),
      axis.title = element_blank(),
      axis.ticks = element_blank(),
      panel.grid = element_blank()
    )
  p_itaconate_pathway[[gene_sel]] <- p1

  file <- file.path(reportDir, paste0("P15038_LungManuscript_ItaconatePathwayExpression_", gene_sel, ".png"))
  png(file = file, width = 2, height = 2, res = 200, units = "in")
  print(p1)
  dev.off()
}

#  * Sub-Section * Itaconate / Irg1 correlation
# ~~~~~~~~~~~~~~~~~~~~~~~~~~~~~~~~~~~~~~~~~~~~~~~~~~~~~~~~~~~~~~~~~~~~~~~~
dat <- data.frame(
  x = data_list[["MRNA"]]["Irg1", ],
  y = data_list[["MET"]]["itaconate", ],
  CAN = colnames(data_list[["MRNA"]])
)
dat$Group <- dat_meta[match(dat$CAN, dat_meta$CAN), "Group"]
dat$Treatment <- getsplit(dat$Group, "_", 1)
dat$Time <- getsplit(dat$Group, "_", 2)

corr_txt <- paste0("Corr = ", sprintf("%2.2f", cor(dat$x, dat$y, use = "pairwise.complete", method = "pearson")))

p <- ggplot(aes(x = x, y = y), data = dat) +
  geom_point() +
  geom_smooth(method = "lm") +
  # scale_colour_manual("Treatment", values = cols) +
  theme_bw() +
  labs(x = "Irg1 [mRNA]", y = "itaconate") +
  xlim(4, 7) +
  ylim(-3, 4) +
  ggplot2::annotate("text", 4, 3.5, label = corr_txt, hjust = 0)

p_itaconate_mRNAvsMET <- p

file <- file.path(reportDir, "P15038_LungManuscript_Itaconate_mRNAvsMET.pdf")
pdf(file = file, width = 4, height = 4, useDingbats = FALSE)
print(p_itaconate_mRNAvsMET)
dev.off()
```

```
## pdf
##   2
```

```
#  * Sub-Section *  global itaconate correlations
# ~~~~~~~~~~~~~~~~~~~~~~~~~~~~~~~~~~~~~~~~~~~~~~~~~~~~~~~~~~~~~~~~~~~~~~~~
res.cor <- WGCNA::cor(t(data_list[["MRNA"]]), t(data_list[["MET"]]), use = "pairwise.complete.obs")
filename <- file.path(reportDir, "P15038_LungManuscript_ResCor_MRNA_MET.rda")
save(res.cor, file = filename)

res_melt <- reshape2::melt(res.cor)
res_melt <- res_melt[abs(res_melt$value) > 0.8, ]
dim(res_melt)
```

```
## [1] 315   3
```

```
res_melt <- res_melt[order(abs(res_melt$value), decreasing = TRUE), ]

tmp <- res.cor[, "itaconate"]
tmp <- tmp[order(abs(tmp), decreasing = TRUE)][1:10]
tmp <- data.frame(Name = names(tmp), Cor = tmp)
tmp$Name <- factor(tmp$Name, levels = rev(tmp$Name))

p1 <- ggplot(aes(x = Name, y = Cor), data = tmp) +
  geom_bar(stat = "identity", width = 0.6, fill = "#54b75c") +
  theme_bw() +
```

```r
  labs(x = "", y = "correlation", title = "itaconate vs. mRNA") +
  theme(axis.text = element_text(size = 10)) +
  coord_flip()

tmp <- res.cor["Irg1", ]
tmp <- tmp[order(abs(tmp), decreasing = TRUE)][1:10]
tmp <- data.frame(Name = names(tmp), Cor = tmp)
tmp$Name <- factor(tmp$Name, levels = rev(tmp$Name))

p2 <- ggplot(aes(x = Name, y = Cor), data = tmp) +
  geom_bar(stat = "identity", width = 0.6, fill = "steelblue") +
  theme_bw() +
  labs(x = "", y = "correlation", title = "Irg1 vs. metabolites") +
  theme(axis.text = element_text(size = 10)) +
  coord_flip()

file <- file.path(reportDir, "P15038_LungManuscript_Itaconate_cor.pdf")
pdf(file = file, width = 9, height = 5, useDingbats = FALSE)
gridExtra::grid.arrange(p1, p2, layout_matrix = matrix(c(1, 2, 2), ncol = 3))
dev.off()
```

```
## pdf
##   2
```

Figure 23 shows the itaconate pathway response profiles, Figure 24 shows the Irg1 mRNA vs Itaconate metabolite correlation, and Figure 24 shows the top 10 correlations for Irg1 mRNA and Itaconate.

15.3 Polyamine pathway

```r
#  * Sub-Section * Polyamine metabolic network
# ~~~~~~~~~~~~~~~~~~~~~~~~~~~~~~~~~~~~~~~~~~~~~~~~~~~~~~~~~~~~~~~~~~~~~~~
col <- attr(idmaps[["MRNA"]], "colors")
names(col) <- names(idmaps[["MRNA"]])

sel_matrix <- matrix(c(
  "MRNA", "Nos2",
  "MRNA", "Arg1",
  "MRNA", "Arg2",
  "MRNA", "Gatm",
  "MRNA", "Oat",
  "MRNA", "Odc1",
  "MRNA", "Otc",
  "MRNA", "Ass1",
  "MRNA", "Sat1",
  "MRNA", "Sat2",
  "MRNA", "Srm",
  "MRNA", "Sms",
  "METABOLITE", "(N(1) + N(8))-acetylspermidine",
  "METABOLITE", "spermine",
  "METABOLITE", "spermidine",
  "METABOLITE", "putrescine",
  "METABOLITE", "N-acetylputrescine",
  "METABOLITE", "arginine",
  "METABOLITE", "creatine",
  "METABOLITE", "creatinine",
  "METABOLITE", "creatine phosphate",
  "METABOLITE", "guanidinoacetate",
  "METABOLITE", "4-guanidinobutanoate",
  "METABOLITE", "guanidinosuccinate",
  "METABOLITE", "beta-guanidinopropanoate",
  "METABOLITE", "N-acetylarginine",
  "METABOLITE", "ornithine",
  "METABOLITE", "urea",
  "METABOLITE", "citrulline",
  "METABOLITE", "argininosuccinate",
  "METABOLITE", "proline"
),
byrow = TRUE, ncol = 2
)

p_polyamine_pathway <- list()
for (i in 1:nrow(sel_matrix)) {
  idmap_sel <- idmaps[[sel_matrix[i, 1]]]
  gene_sel <- sel_matrix[i, 2]

  if (gene_sel %in% idmap_sel[[1]]$nodeLabel) {
```

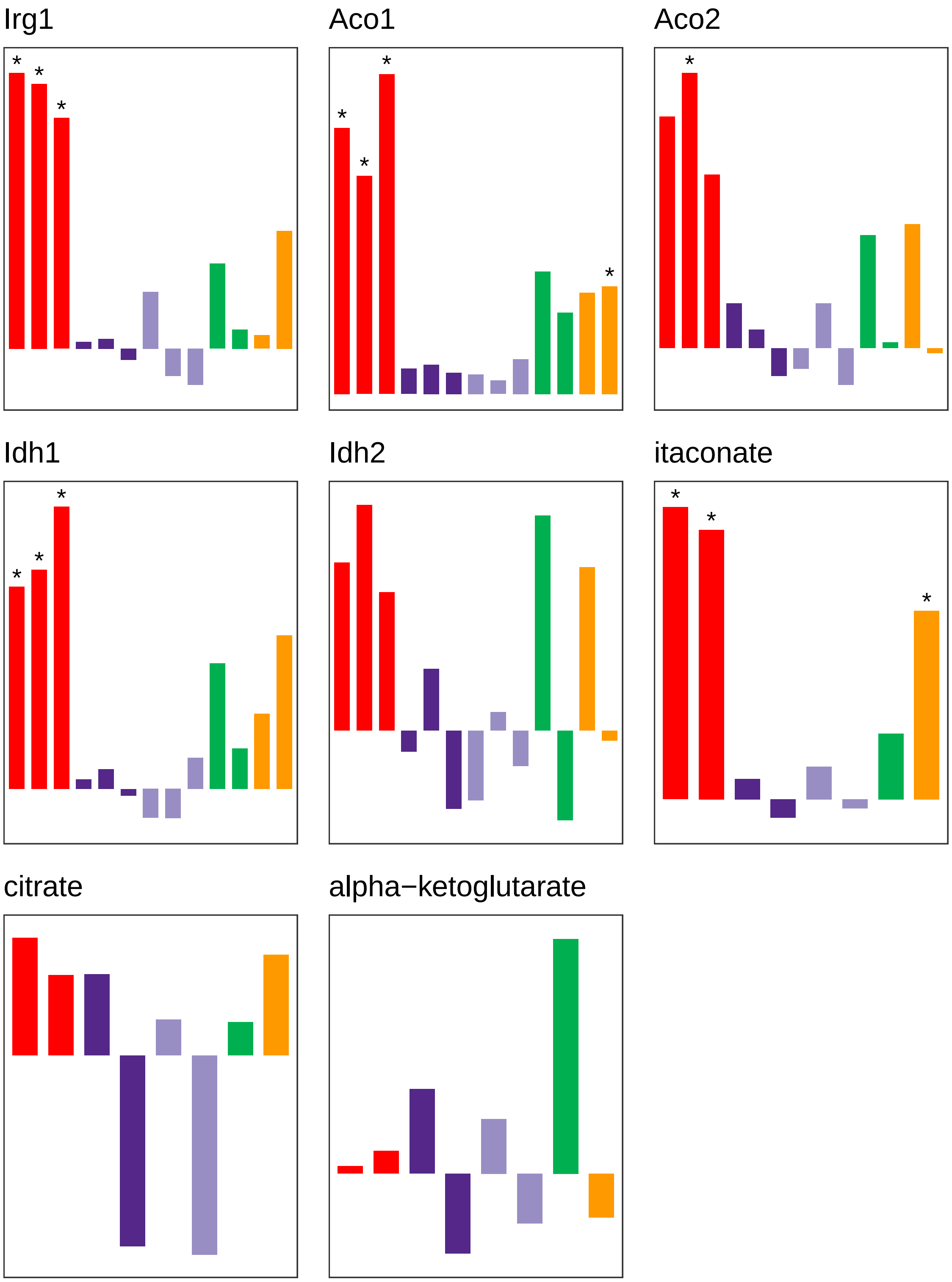

Figure 23: Itaconate metabolic pathway.

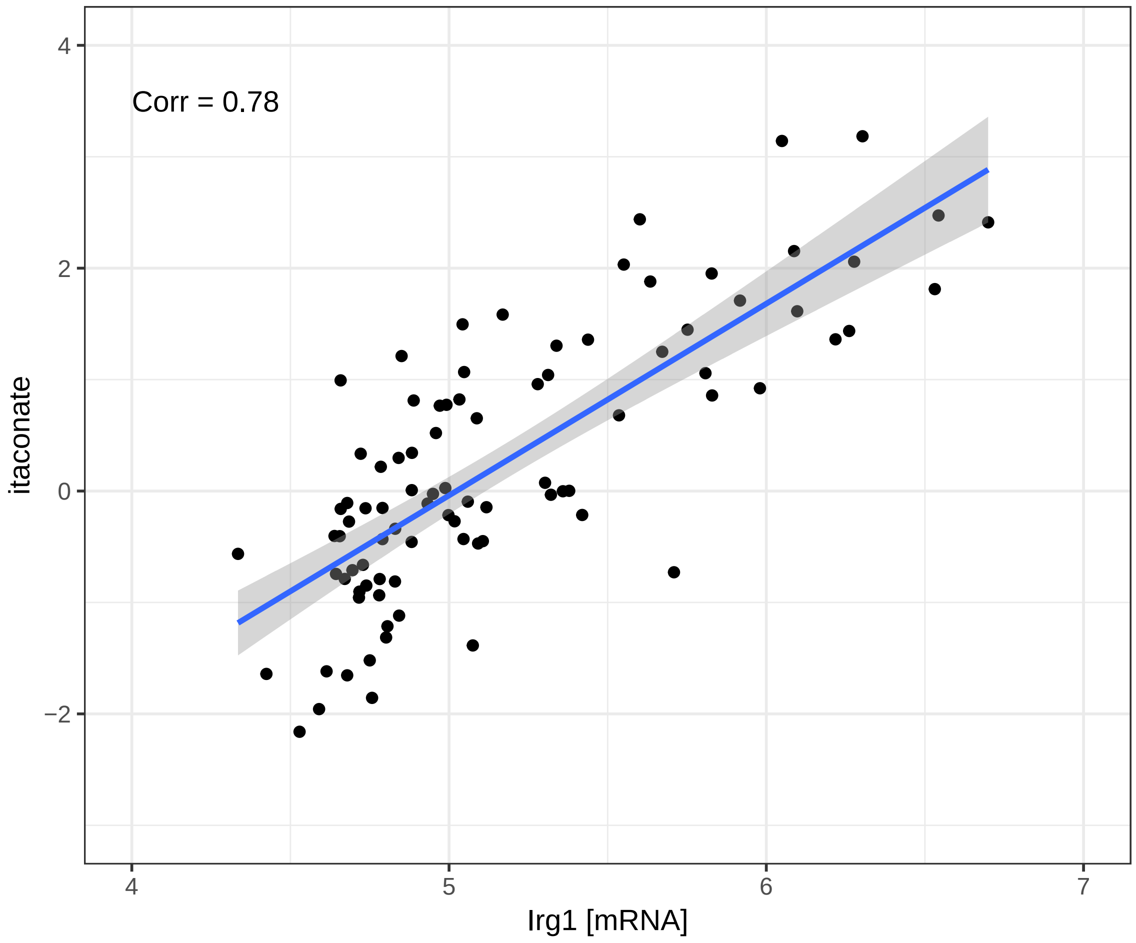

Figure 24: Correlation between Irg1 mRNA expression and itaconate abundance

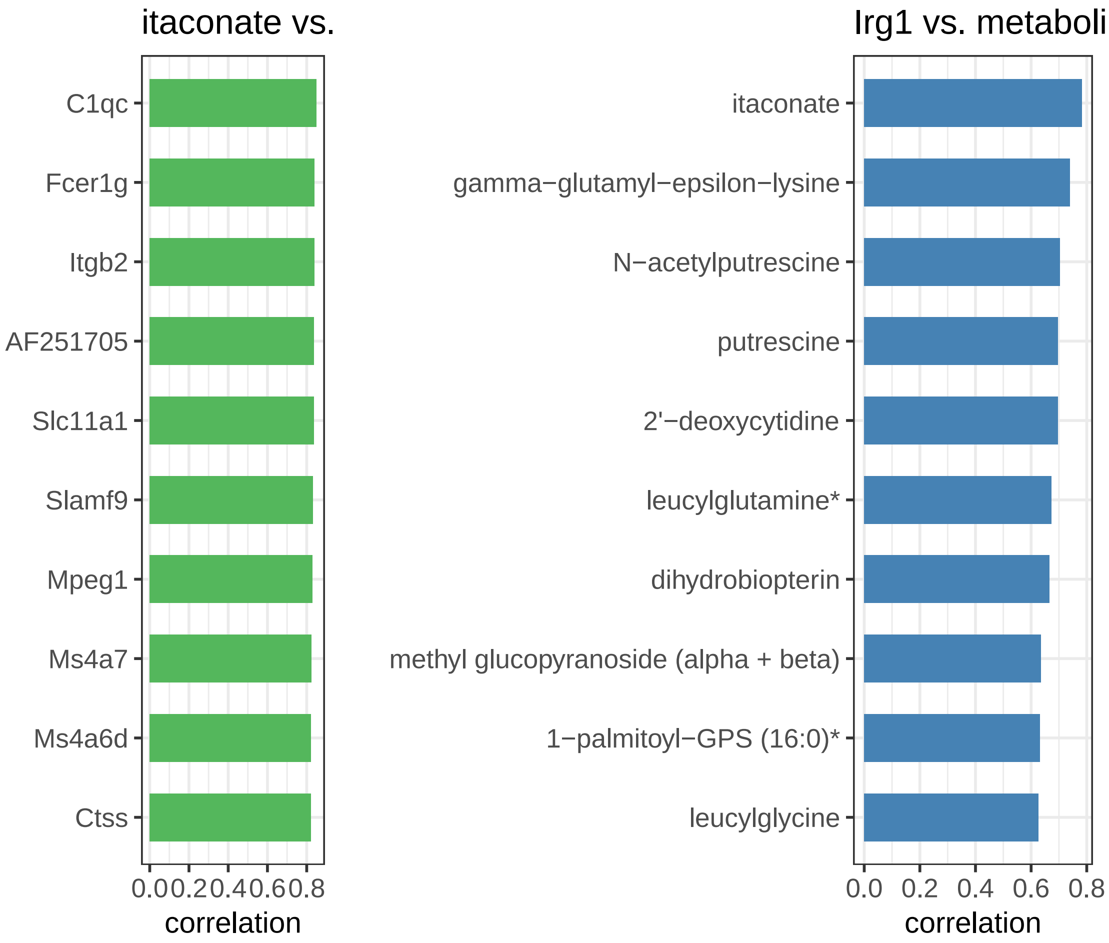

Figure 25: (left) Top 10 correlations of Irg1 mRNA expression against all metabolites. (right) Top 10 correlations of itaconate abundance against all mRNAs

```
    dat <- data.frame(
      fc = as.numeric(getFromIDMAP(idmap_sel, gene_sel, type = "foldChange", exact = TRUE)),
      apv = as.numeric(getFromIDMAP(idmap_sel, gene_sel, type = "adj.p.value", exact = TRUE)),
      contrasts = names(idmap_sel)
    )
    dat$sig <- ifelse(dat$apv < 0.05, "*", "")
    dat$contrasts <- factor(dat$contrasts, levels = dat$contrasts)

    p <- ggplot(aes(x = contrasts, y = fc, fill = contrasts), data = dat) +
      geom_bar(stat = "identity", width = 0.7) +
      scale_fill_manual("", values = col, guide = FALSE) +
      geom_text(aes(x = contrasts, y = fc + (max(abs(fc)) / 30 * sign(fc)), label = sig), data = dat, colour = "black") +
      labs(x = "", y = "log2 fold-change", title = gene_sel) +
      theme_bw() +
      theme(axis.text.x = element_text(angle = 90, hjust = 1, vjust = 0.5))
    p1 <- p +
      theme(
        axis.text = element_blank(),
        axis.title = element_blank(),
        axis.ticks = element_blank(),
        panel.grid = element_blank()
      )
    p_polyamine_pathway[[gene_sel]] <- p1

    file <- file.path(reportDir, paste0("P15038_LungManuscript_PolyaminePathwayExpression_", gene_sel, ".png"))
    png(file = file, width = 2, height = 2, res = 200, units = "in")
    print(p1)
    dev.off()
  } else {
    cat("Did not find ", gene_sel, "\n")
  }
}
```

```
## Did not find  Ass1
## Did not find  Sat1

## Did not find  Srm

## Did not find  ornithine
```

Figure 26 shows the polyamine pathway response profiles.

15.4 Expression profiles for selected immune-related miRNAs

```
sel_genes <- rev(c("mmu-miR-146a-5p", "mmu-miR-21a-5p", "mmu-miR-2137"))

idmap <- idmaps[["MIRNA"]]
sig_mir <- unique(unlist(lapply(getDEG(idmap), function(x) as.character(x$nodeLabel))))
sig_mir <- sig_mir[!is.na(sig_mir)]

p <- getIDMAPheatmapGG(idmap, sel_genes, cluster.row = FALSE, cex.labx = 2, cex.laby = 1.5, title = NULL,
                       col.lab = "black", object.only = TRUE)

pdf(file = file.path(reportDir, "P15038_LungManuscript_microRNA_HM.pdf"), width = 5, height = 2)
print(p)
dev.off()
```

```
## pdf
##   2
```

Figure 26 shows the polyamine pathway response profiles.

16 Oxidative stress-related effects

16.1 Activation of hemoglobin–biliverdin–bilirubin pathway

```
#  * Sub-Section * Bilirubin metabolic network
# ~~~~~~~~~~~~~~~~~~~~~~~~~~~~~~~~~~~~~~~~~~~~~~~~~~~~~~~~~~~~~~~~~~~~~~~
col <- attr(idmaps[["MRNA"]], "colors")
names(col) <- names(idmaps[["MRNA"]])
```

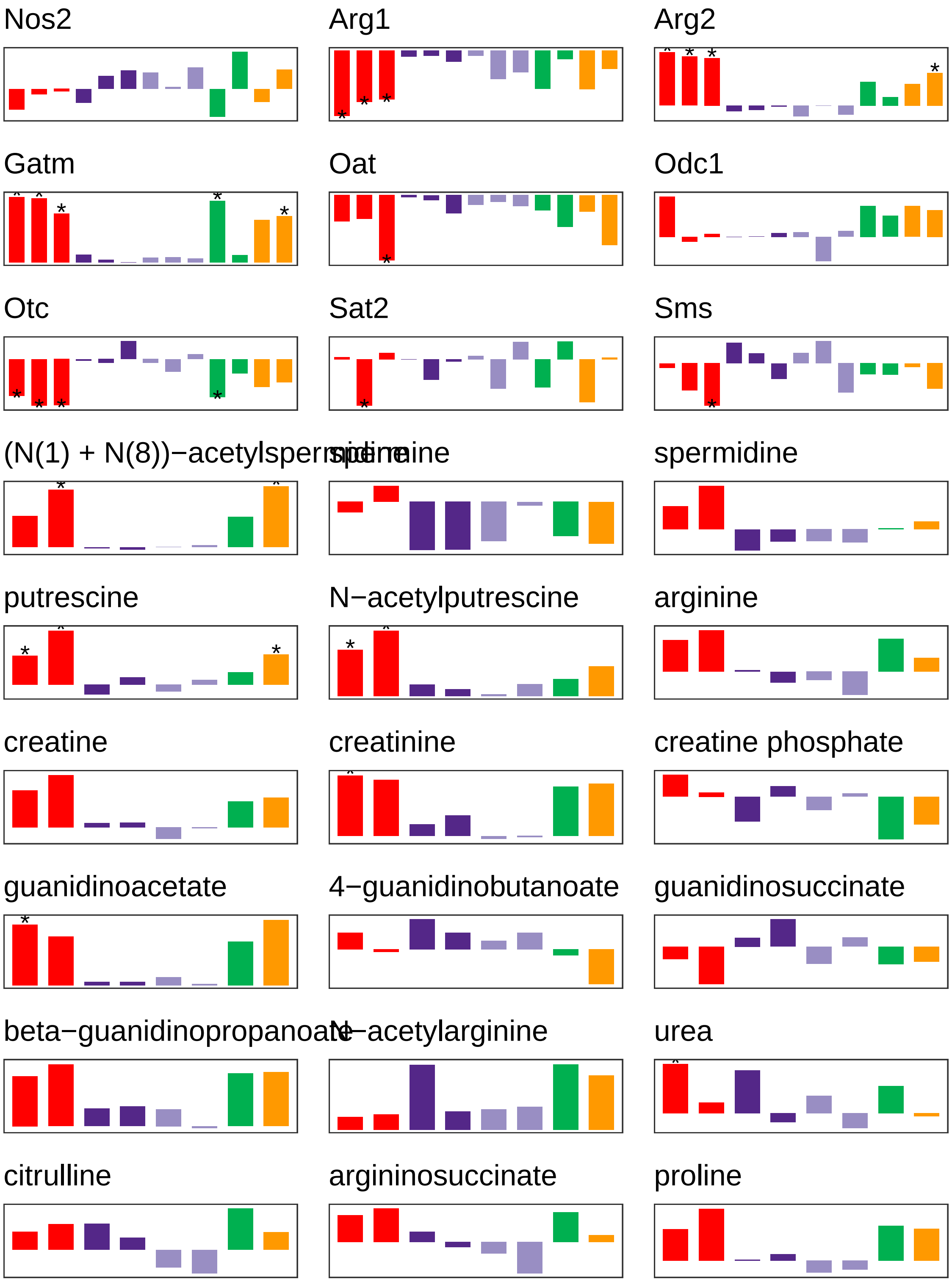

Figure 26: Polyamine metabolic pathway.

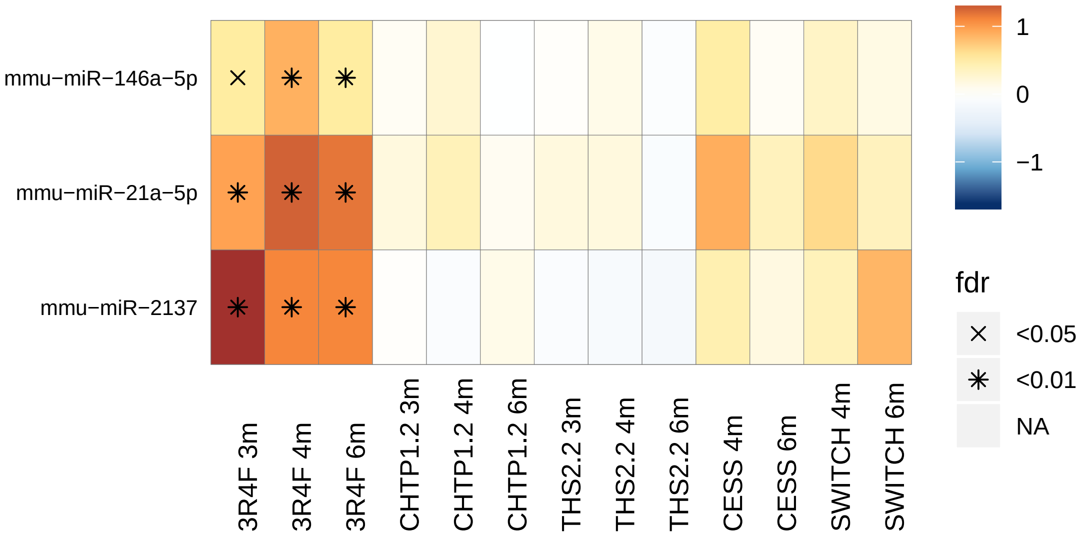

Figure 27: Expression profiles for selected immune-related miRNAs.

```
sel_matrix <- matrix(c(
  "MRNA", "Hmox1",
  "MRNA", "Hmox2",
  "PROTEIN", "Hmox1",
  "PROTEIN", "Hmox2",
  "PROTEIN", "Blvrb",
  "MRNA", "Blvrb",
  "MRNA", "Fth1",
  "MRNA", "Ftl1",
  "PROTEIN", "Fth1",
  "PROTEIN", "Ftl1",
  "METABOLITE", "bilirubin",
  "METABOLITE", "biliverdin"
),
byrow = TRUE, ncol = 2
)

p_hmox_pathway <- list()
for (i in 1:nrow(sel_matrix)) {
  idmap_sel <- idmaps[[sel_matrix[i, 1]]]
  gene_sel <- sel_matrix[i, 2]

  if (gene_sel %in% idmap_sel[[1]]$nodeLabel) {
    dat <- data.frame(
      fc = as.numeric(getFromIDMAP(idmap_sel, gene_sel, type = "foldChange", exact = TRUE)),
      apv = as.numeric(getFromIDMAP(idmap_sel, gene_sel, type = "adj.p.value", exact = TRUE)),
      contrasts = names(idmap_sel)
    )
    dat$sig <- ifelse(dat$apv < 0.05, "*", "")
    dat$contrasts <- factor(dat$contrasts, levels = dat$contrasts)

    p <- ggplot(aes(x = contrasts, y = fc, fill = contrasts), data = dat) +
      geom_bar(stat = "identity", width = 0.7) +
      scale_fill_manual("", values = col, guide = FALSE) +
      geom_text(aes(x = contrasts, y = fc + (max(abs(fc)) / 30 * sign(fc)), label = sig), data = dat, colour = "black") +
      labs(x = "", y = "log2 fold-change", title = paste0(gene_sel, " ", sel_matrix[i, 1])) +
      theme_bw() +
      theme(axis.text.x = element_text(angle = 90, hjust = 1, vjust = 0.5))
    p1 <- p +
      theme(
        axis.text = element_blank(),
        axis.title = element_blank(),
        axis.ticks = element_blank(),
        panel.grid = element_blank(),
        title = element_text(size = 8)
      )
    p_hmox_pathway[[gene_sel]] <- p1

    file <- file.path(reportDir, paste0("P15038_LungManuscript_BilirubinPathwayExpression_", sel_matrix[i, 1], "_", gene_sel, ".png"))
    png(file = file, width = 2, height = 2, res = 200, units = "in")
```

```
    print(p1)
    dev.off()
  } else {
    cat("Did not find ", gene_sel, "\n")
  }
}
```

```
## Did not find  Hmox1
## Did not find  Ftl1
```

Figure 28 shows the hemoglobin–biliverdin–bilirubin pathway response profiles.

16.2 Oxidative stress-related metabolites, proteins, and network

```
#  * Sub-Section *  Ox Stress metabolites
# ~~~~~~~~~~~~~~~~~~~~~~~~~~~~~~~~~~~~~~~~~~~~~~~~~~~~~~~~~~~~~~~~~~~~~~~~
# metabolites
dat <- dats[["METABOLITE"]]
idmap <- idmaps[["METABOLITE"]]

# metabolite selection, based on Metabolon report
met_sel <- c(
  "glutathione, reduced (GSH)",
  "glutathione, oxidized (GSSG)",
  "cysteine-glutathione disulfide",
  "cystathionine",
  "N-acetylmethionine sulfoxide",
  "methionine sulfoxide",
  "alpha-tocopherol"
)

p <- getIDMAPheatmapGG(idmap, met_sel,
  title = "", cluster.row = FALSE, col.group = NULL,
  cex.labx = 1.7, cex.laby = 1.4, col.lab = "black", cex.facet = 1.2,
  object.only = TRUE
) +
  theme(
    axis.text.x = element_text(angle = 90, vjust = 0.5, hjust = 1, size = 10),
    axis.text.y = element_text(angle = 0, vjust = 0.5, hjust = 1, size = 10)
  )
p_oxstress_met <- p

pdf(file = file.path(reportDir, "P15038_LungManuscript_OxStress_Metabolites_HM.pdf"), width = 5.3, height = 3)
print(p_oxstress_met)
dev.off()
```

```
## pdf
##   2
```

```
#  * Sub-Section *  Ox stress gene / protein sets
# ~~~~~~~~~~~~~~~~~~~~~~~~~~~~~~~~~~~~~~~~~~~~~~~~~~~~~~~~~~~~~~~~~~~~~~~~
# proteins
idmap <- idmaps[["PROTEIN"]]

sig_genes <- unique(unlist(lapply(getDEG(idmap), function(x) as.character(x$nodeLabel))))
sig_genes <- sig_genes[!is.na(sig_genes)]

gsc_h <- getGMT_GS("../DATA/EXTERNAL/h.all.v6.2.symbols.gmt")
gsc_c2 <- getGMT_GS("../DATA/EXTERNAL/c2.all.v6.2.symbols.gmt")

sel_genes <- gsc_h[["HALLMARK_REACTIVE_OXIGEN_SPECIES_PATHWAY"]]
sel_genes <- c(sel_genes, gsc_c2[["KEGG_METABOLISM_OF_XENOBIOTICS_BY_CYTOCHROME_P450"]])
sel_genes <- GetOrtholog(sel_genes, species_from = "Hs", species_to = "Mm")
```

```
## Loading objects:
##   HCOP_ORTHOLOGY_TABLE
```

```
sel_genes <- unique(sel_genes)
sel_genes <- sel_genes[!is.na(sel_genes)]

p <- getIDMAPheatmapGG(idmap, intersect(sel_genes, sig_genes), cluster.row = TRUE, cex.labx = 2, cex.laby = 1.5,
                       title = NULL, col.lab = "black", object.only = TRUE)
p_oxstress_prot <- p

pdf(file = file.path(reportDir, "P15038_LungManuscript_OxStressXeno_Proteins_HM.pdf"), width = 5, height = 5)
print(p_oxstress_prot)
dev.off()
```

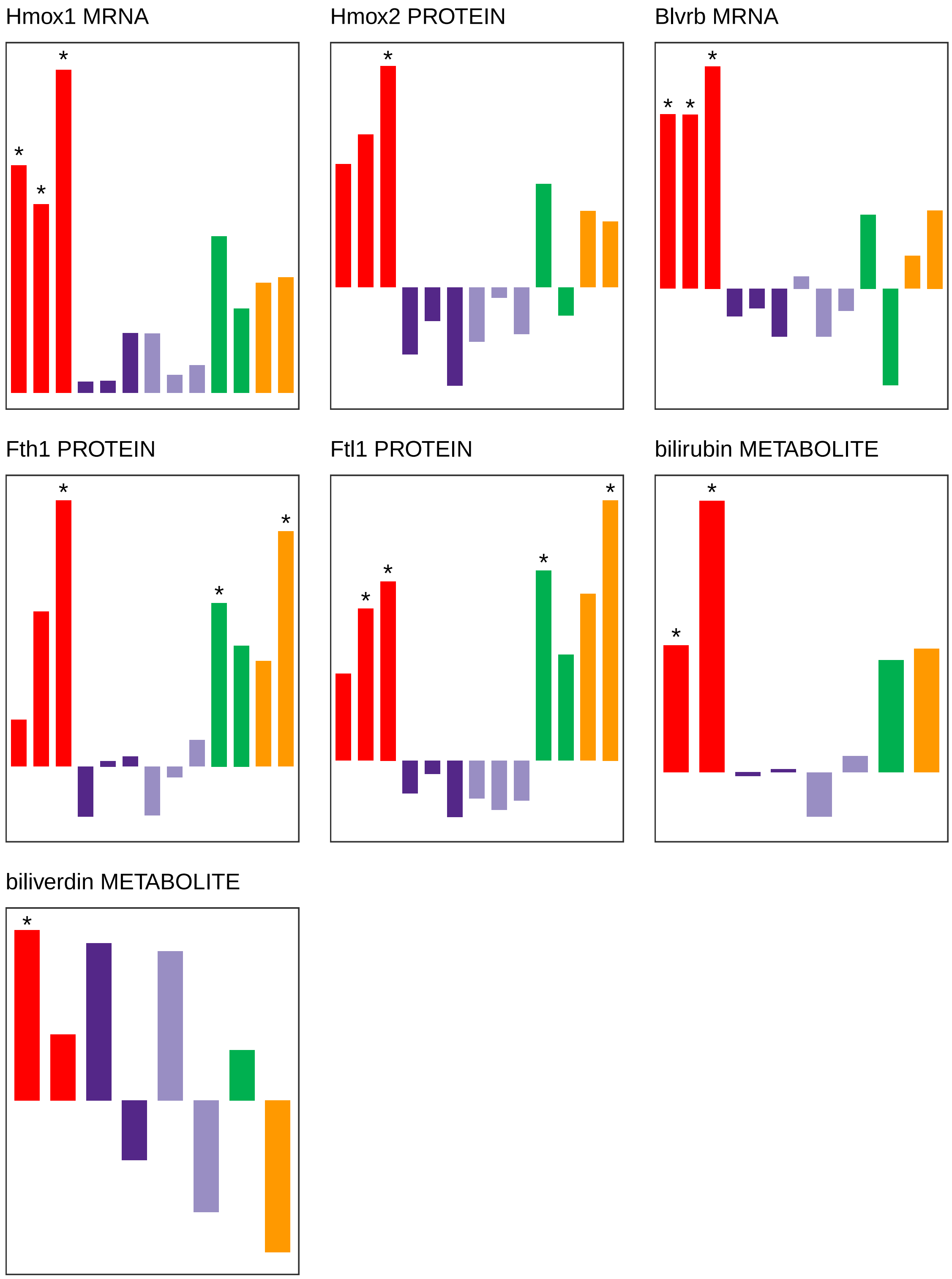

Figure 28: Hemoglobin–biliverdin–bilirubin pathway.

```
## pdf
##   2
```

```
# mRNAs
idmap <- idmaps[["MRNA"]]
sig_genes <- unique(unlist(lapply(getDEG(idmap), function(x) as.character(x$nodeLabel))))
sig_genes <- sig_genes[!is.na(sig_genes)]

gsc_h <- getGMT_GS("../DATA/EXTERNAL/h.all.v6.2.symbols.gmt")
gsc_c2 <- getGMT_GS("../DATA/EXTERNAL/c2.all.v6.2.symbols.gmt")

sel_genes <- gsc_h[["HALLMARK_REACTIVE_OXIGEN_SPECIES_PATHWAY"]]
sel_genes <- c(sel_genes, gsc_c2[["KEGG_METABOLISM_OF_XENOBIOTICS_BY_CYTOCHROME_P450"]])
sel_genes <- GetOrtholog(sel_genes, species_from = "Hs", species_to = "Mm")
```

```
## Loading objects:
##   HCOP_ORTHOLOGY_TABLE
```

```
sel_genes <- unique(sel_genes)
sel_genes <- sel_genes[!is.na(sel_genes)]

p <- getIDMAPheatmapGG(idmap, intersect(sel_genes, sig_genes), cluster.row = TRUE, cex.labx = 2,
                       cex.laby = 1.5, title = NULL, col.lab = "black", object.only = TRUE)
p_oxstress_mRNA <- p

pdf(file = file.path(reportDir, "P15038_LungManuscript_OxStressXeno_mRNAs_HM.pdf"), width = 5, height = 8)
print(p_oxstress_mRNA)
dev.off()
```

```
## pdf
##   2
```

```
nw_name <- "CST / Oxidative Stress"
sel_nw <- which(npa_list$networks() == nw_name)
npa <- subset(npa_list, sel_nw, NULL)

file <- file.path(reportDir, "P15038_LungManuscriptOxStress_NPA.pdf")
pdf(file = file, width = 5, height = 6, useDingbats = FALSE)
barplot(npa,
        legend.text = FALSE,
        col = attr(idmaps[["MRNA"]], "colors"),
        bg = "white",
        main = nw_name)
dev.off()
```

```
## pdf
##   2
```

Figure 29 shows the response profiles of oxidative-stress related metabolites, Figure 30 shows the response profiles of oxidative-stress related proteins, and Figure 31 shows the response profiles of oxidative-stress related mRNA transcripts. Figure 32 shows the perturbation of the oxidative stress network (based on transcriptomics data).

17 Effects on lipid metabolism

17.1 Lipid abundance for PEs and LPEs, glycerophospholipid metabolism, and surfactant

```
#  * Sub-Section *  FA metabolism
# ~~~~~~~~~~~~~~~~~~~~~~~~~~~~~~~~~~~~~~~~~~~~~~~~~~~~~~~~~~~~~~~~~~~~~~~~
# KEGG fatty acid biosynthesis pathway
# KEGG fatty acid degradation
idmap <- idmaps[["PROTEIN"]]

sig_genes <- unique(unlist(lapply(getDEG(idmap), function(x) as.character(x$nodeLabel))))
sig_genes <- sig_genes[!is.na(sig_genes)]

gsc_c2 <- getGMT_GS("../DATA/EXTERNAL/c2.all.v6.2.symbols.gmt")

sel_genes <- gsc_c2[["KEGG_FATTY_ACID_METABOLISM"]]
sel_genes <- GetOrtholog(sel_genes, species_from = "Hs", species_to = "Mm")
```

```
## Loading objects:
##   HCOP_ORTHOLOGY_TABLE
```

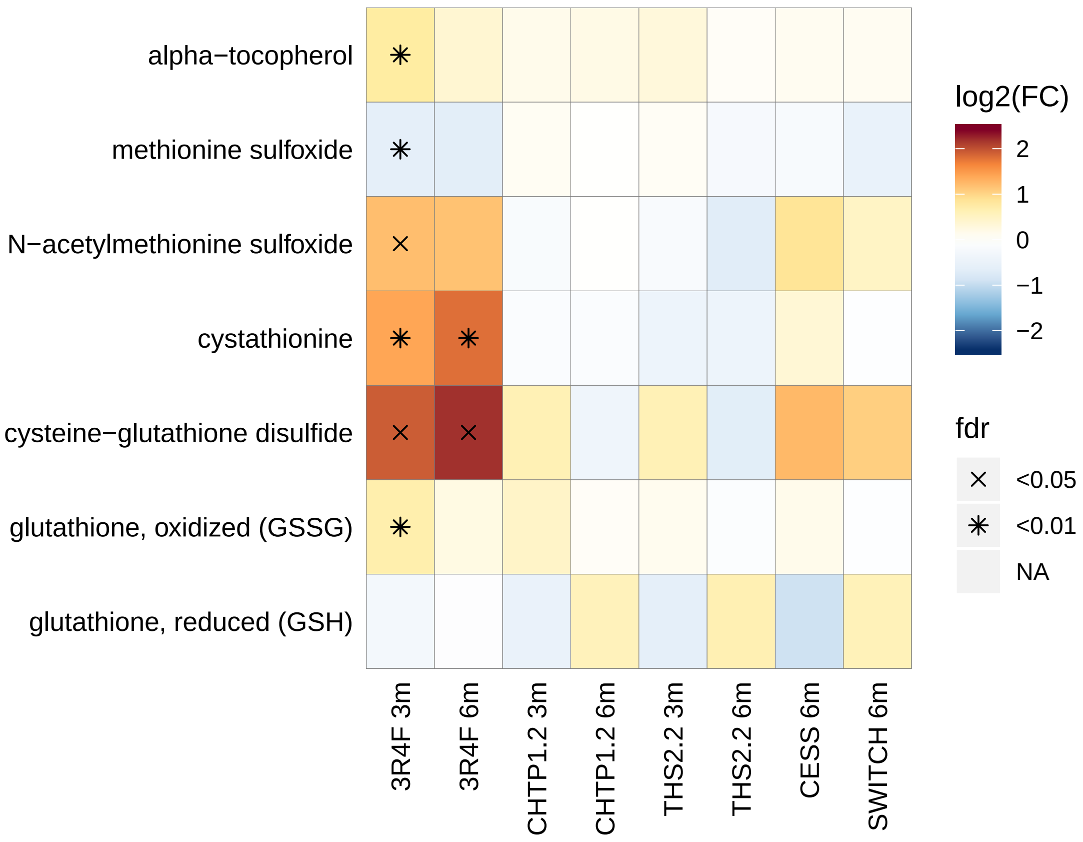

Figure 29: Oxidative stress-related metabolites.

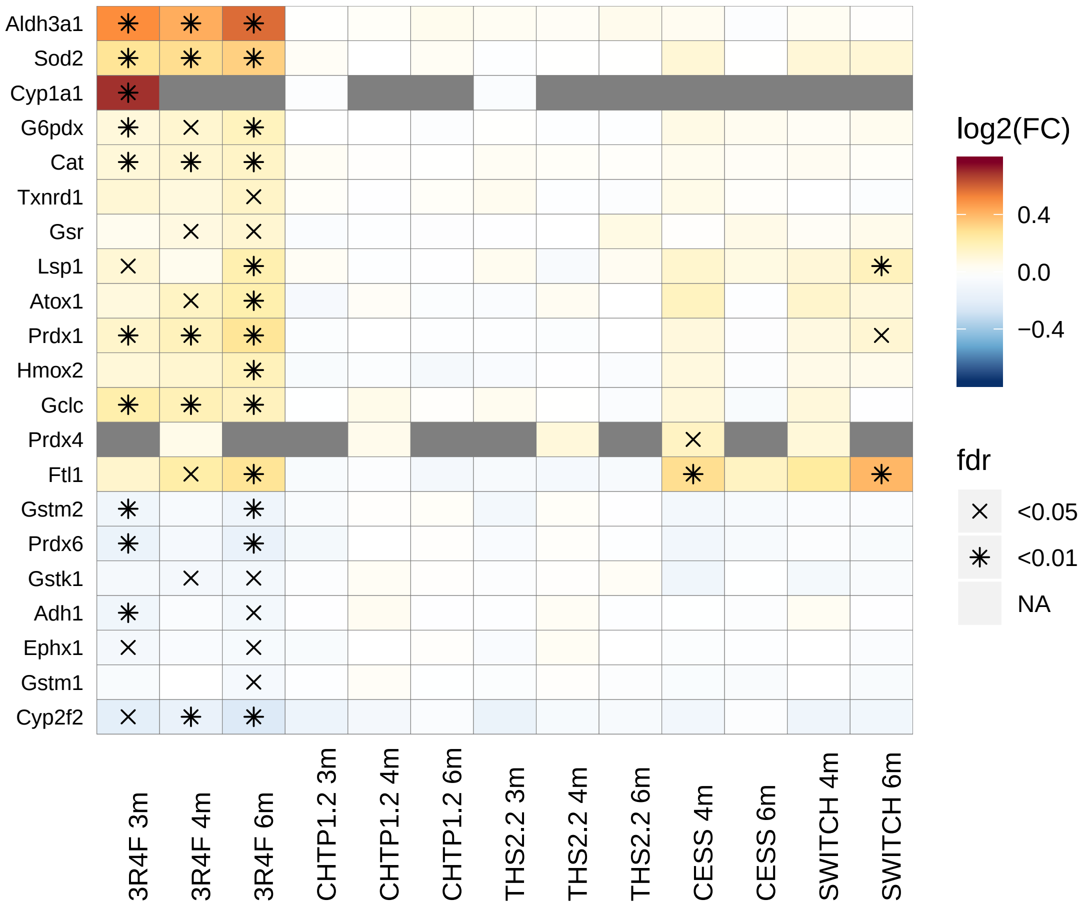

Figure 30: Oxidative stress-related proteins.

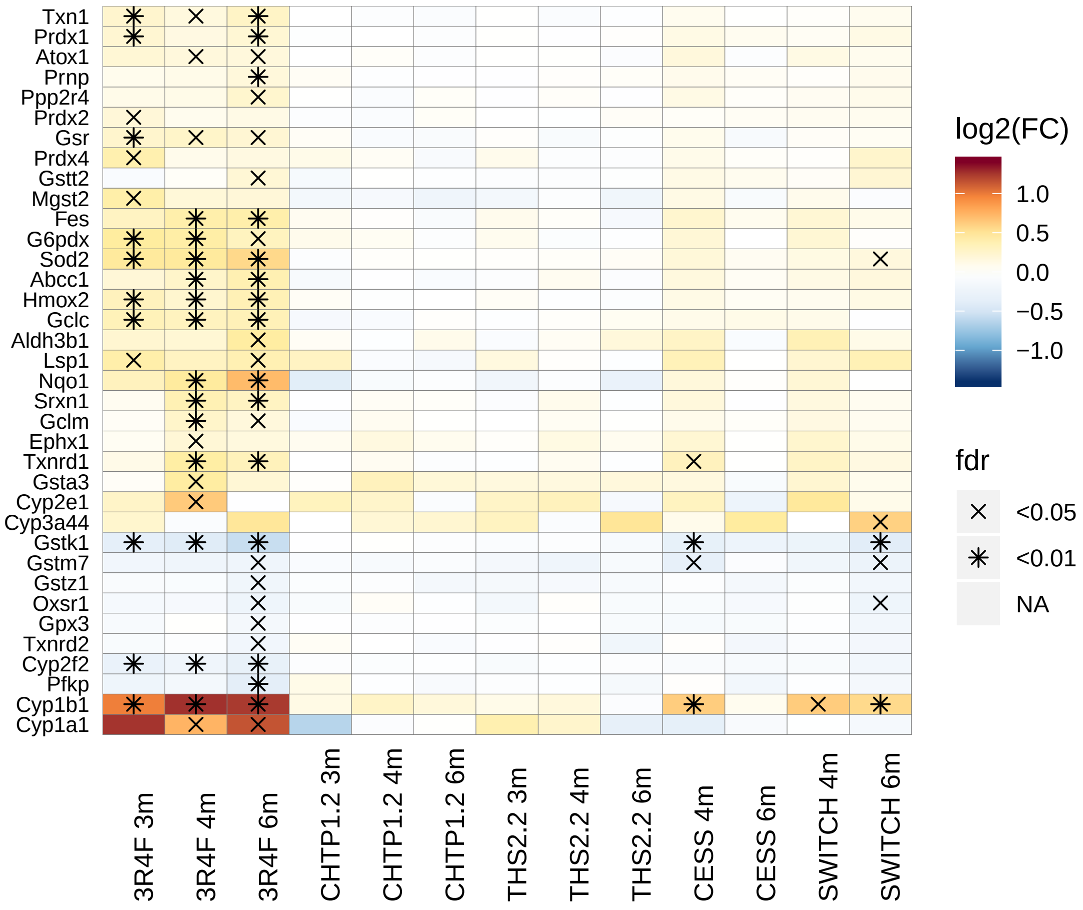

Figure 31: Oxidative stress-related mRNA transcripts.

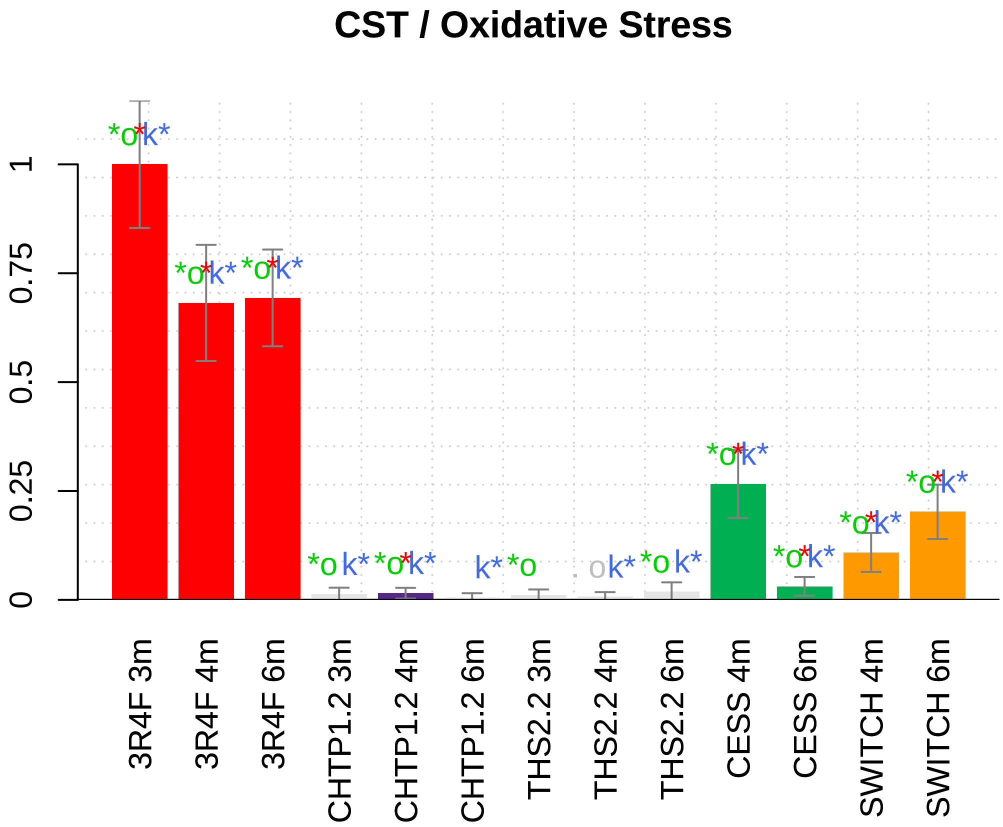

Figure 32: Perturbation of the oxidative stress network.

```r
sel_genes <- unique(sel_genes)
sel_genes <- sel_genes[!is.na(sel_genes)]

p <- getIDMAPheatmapGG(idmap, intersect(sel_genes, sig_genes), cluster.row = TRUE, cex.labx = 2,
                       cex.laby = 1.5, title = NULL, col.lab = "black", object.only = TRUE)
p_FAMet_proteins <- p

pdf(file = file.path(reportDir, "P15038_LungManuscript_FA-METABOLISM_Proteins_HM.pdf"), width = 5, height = 5)
print(p_FAMet_proteins)
dev.off()
```

```
## pdf
##   2
```

```r
#  * Sub-Section * surfactant
# ~~~~~~~~~~~~~~~~~~~~~~~~~~~~~~~~~~~~~~~~~~~~~~~~~~~~~~~~~~~~~~~~~~~~~~~~
idmap <- idmaps[["PROTEIN"]]
sel_surfactant_proteins <- c(
  "Sftpd", "Sftpa1", "Lpcat1", "Slc34a2", "Abca3", "Sftpc",
  "Sftpb", "Prdx6"
)

sig_genes <- unique(unlist(lapply(getDEG(idmap), function(x) as.character(x$nodeLabel))))
sig_genes <- sig_genes[!is.na(sig_genes)]

sel_genes <- sel_surfactant_proteins
sel_genes <- unique(sel_genes)
sel_genes <- sel_genes[!is.na(sel_genes)]

p <- getIDMAPheatmapGG(idmap, intersect(sel_genes, sig_genes), cluster.row = TRUE,
                       cex.labx = 2, cex.laby = 1.5, title = NULL, col.lab = "black",
                       object.only = TRUE)
p_surfactant_proteins <- p

pdf(file = file.path(reportDir, "P15038_LungManuscript_Surfactant_Proteins_HM.pdf"), width = 5, height = 3.7)
print(p_surfactant_proteins)
dev.off()
```

```
## pdf
##   2
```

```r
idmap <- idmaps[["MRNA"]]
sig_genes <- unique(unlist(lapply(getDEG(idmap), function(x) as.character(x$nodeLabel))))
sig_genes <- sig_genes[!is.na(sig_genes)]

sel_genes <- sel_surfactant_proteins
sel_genes <- unique(sel_genes)
sel_genes <- sel_genes[!is.na(sel_genes)]

p <- getIDMAPheatmapGG(idmap, intersect(sel_genes, sig_genes), cluster.row = TRUE, cex.labx = 2,
                       cex.laby = 1.5, title = NULL, col.lab = "black", object.only = TRUE)
p_surfactant_MRNAs <- p

pdf(file = file.path(reportDir, "P15038_LungManuscript_Surfactant_mRNAs_HM.pdf"), width = 5, height = 4.7)
print(p_surfactant_MRNAs)
dev.off()
```

```
## pdf
##   2
```

```r
# surfactant lipids
# PC 16:0/16:0
# PC 16:0/16:1
# PC 14:0/16:0

# PG 16:0/16:0
# PG 16:0/18:1
# PG 16:0/16:2

idmap <- idmaps[["LIPID"]]
sig_mets <- unique(unlist(lapply(getDEG(idmap), function(x) as.character(x$nodeLabel))))
sig_mets <- sig_mets[!is.na(sig_mets)]
sel_mets <- c("PC 32:0", "PC 32:1", "PC 30:0", "PG 32:0", "PG 34:1", "PG 34:2")

p <- getIDMAPheatmapGG(idmap, intersect(sel_mets, sig_mets), cluster.row = FALSE, cex.labx = 2,
                       cex.laby = 1.5, title = NULL, col.lab = "black", object.only = TRUE)
p_surfactant_lipids <- p

pdf(file = file.path(reportDir, "P15038_LungManuscript_Surfactant_Lipids_HM.pdf"), width = 5, height = 3)
print(p_surfactant_lipids)
dev.off()
```

```
## pdf
##   2
```

```r
#  * Sub-Section *  Lipid metabolism imbalance
# ~~~~~~~~~~~~~~~~~~~~~~~~~~~~~~~~~~~~~~~~~~~~~~~~~~~~~~~~~~~~~~~~~~~~~~~~~~~~
# https://journals.plos.org/plosone/article?id=10.1371/journal.pone.0092498
# http://erj.ersjournals.com/content/46/5/1451

# Lipids
compound_dat <- readr::read_delim("../DATA/EXTERNAL/cmp_extracted.table.txt", "\t", col_names = FALSE) %>%
    as.data.frame()
colnames(compound_dat) <- c("cpn_ID", "names")
compound_dat$label <- getsplit(compound_dat$names, ";", 1)
lipid2kegg <- read.csv("../DATA/P15038_LungManuscript_LipidMappingTable3_bt.csv")

idmap <- idmaps[["LIPID"]]
sig_mets <- unique(unlist(lapply(getDEG(idmap), function(x) as.character(x$nodeLabel))))
sig_mets <- sig_mets[!is.na(sig_mets)]

sel_mets_LPE <- grep("^LPE ", idmap[[1]]$nodeLabel, value = TRUE)
sel_mets_PE <- grep("^PE ", idmap[[1]]$nodeLabel, value = TRUE)
sel_mets <- c(sel_mets_LPE, sel_mets_PE)

p <- getIDMAPheatmapGG(idmap, rev(intersect(sel_mets, sig_mets)), cluster.row = FALSE, cex.labx = 2,
                       cex.laby = 1.5, title = NULL, col.lab = "black", object.only = TRUE)
p_lpe_pe_lipids <- p

pdf(file = file.path(reportDir, "P15038_LungManuscript_LPE_Lipids_HM.pdf"), width = 5, height = 7)
print(p_lpe_pe_lipids)
dev.off()
```

```
## pdf
##   2
```

```r
#  * Sub-Section *  LIPID CLASS SUMMARY STAT HEATMAP
# ~~~~~~~~~~~~~~~~~~~~~~~~~~~~~~~~~~~~~~~~~~~~~~~~~~~~~~~~~~~~~~~~~~~~~~~~~~~~
dat <- dats[["LIPID"]]
X <- (2^dat$data) - 1
X[is.na(X)] <- 0
lc <- getsplit(rownames(X), " ", 1)
X <- do.call(rbind, by(X, lc, function(x) apply(x, 2, sum, na.rm = TRUE)))
X <- log2(X)
idmap <- getLimmaResults(X, dat$L, dat$CO, covariates = NULL, include.ordinary.pvalues = TRUE)
```

```
## Computing Limma Models...
## ###### Estimating 3R4F_3m vs Sham_3m ...
## (Intercept) TTTB__3R4F_3m
## TTTB__3R4F_3m
##
## ###### Estimating 3R4F_4m vs Sham_4m ...
## (Intercept) TTTB__3R4F_4m
## TTTB__3R4F_4m
##
## ###### Estimating 3R4F_6m vs Sham_6m ...
## (Intercept) TTTB__3R4F_6m
## TTTB__3R4F_6m
##
## ###### Estimating CHTP1.2_3m vs Sham_3m ...
## (Intercept) TTTB__CHTP1.2_3m
## TTTB__CHTP1.2_3m
##
## ###### Estimating CHTP1.2_4m vs Sham_4m ...
## (Intercept) TTTB__CHTP1.2_4m
## TTTB__CHTP1.2_4m
##
## ###### Estimating CHTP1.2_6m vs Sham_6m ...
## (Intercept) TTTB__CHTP1.2_6m
## TTTB__CHTP1.2_6m
##
## ###### Estimating THS2.2_3m vs Sham_3m ...
## (Intercept) TTTB__THS2.2_3m
## TTTB__THS2.2_3m
##
## ###### Estimating THS2.2_4m vs Sham_4m ...
## (Intercept) TTTB__THS2.2_4m
## TTTB__THS2.2_4m
##
## ###### Estimating THS2.2_6m vs Sham_6m ...
## (Intercept) TTTB__THS2.2_6m
## TTTB__THS2.2_6m
##
## ###### Estimating CESS_4m vs Sham_4m ...
```

```
## (Intercept) TTTB__CESS_4m
## TTTB__CESS_4m
##
## ###### Estimating CESS_6m vs Sham_6m ...
## (Intercept) TTTB__CESS_6m
## TTTB__CESS_6m
##
## ###### Estimating SWITCH_4m vs Sham_4m ...
## (Intercept) TTTB__SWITCH_4m
## TTTB__SWITCH_4m
##
## ###### Estimating SWITCH_6m vs Sham_6m ...
## (Intercept) TTTB__SWITCH_6m
## TTTB__SWITCH_6m
```

```
idmap <- lapply(idmap, function(tmp) {
  tmp$moderated.adj.p.value <- tmp$adj.p.value
  tmp$moderated.p.value <- tmp$p.value
  tmp$moderated.t <- tmp$t
  tmp$adj.p.value <- tmp$ord.adj.p.value
  tmp$p.value <- tmp$ord.p.value
  tmp$t <- tmp$ord.t
  return(tmp)
})
names(idmap) <- gsub(" vs.+", "", names(idmap))

p <- getIDMAPheatmapGG(idmap,
                       idmap[[1]]$nodeLabel,
                       cluster.row = FALSE,
                       cex.labx = 2,
                       cex.laby = 1.5,
                       title = NULL,
                       col.lab = "black",
                       object.only = TRUE)
p_lipid_class_hm <- p

pdf(file = file.path(reportDir, "P15038_LungManuscript_LipidClass_HM.pdf"), width = 5, height = 4)
print(p_lipid_class_hm)
dev.off()
```

```
## pdf
##   2
```

```
#  * Sub-Section * phosphoglycerolipid pathway etc
# ~~~~~~~~~~~~~~~~~~~~~~~~~~~~~~~~~~~~~~~~~~~~~~~~~~~~~~~~~~~~~~~~~~~~~~~~~~
idmap <- idmaps[["MRNA"]]

sig_genes <- unique(unlist(lapply(getDEG(idmap), function(x) as.character(x$nodeLabel))))
sig_genes <- sig_genes[!is.na(sig_genes)]

gsc_c2 <- getGMT_GS("../DATA/EXTERNAL/c2.all.v6.2.symbols.gmt")

sel_genes <- gsc_c2[["KEGG_GLYCEROPHOSPHOLIPID_METABOLISM"]]
sel_genes <- GetOrtholog(sel_genes, species_from = "Hs", species_to = "Mm")
```

```
## Loading objects:
##   HCOP_ORTHOLOGY_TABLE
```

```
#complement
sel_genes <- c(sel_genes, "Pisd", "Pla2g7", "Lpcat1", "Pld3", "Pla2g2d", "Lpcat2b", "Pnpla7", "Lcat")
sel_genes <- unique(sel_genes)
sel_genes <- sel_genes[!is.na(sel_genes)]

p_glycerolipid_met <- getIDMAPheatmapGG(idmap, intersect(sel_genes, sig_genes), cluster.row = TRUE,
                                        cex.labx = 2, cex.laby = 1.5, title = NULL, col.lab = "black",
                                        object.only = TRUE)
pdf(file = file.path(reportDir, "P15038_LungManuscript_GLYCEROPHOSPHOLIPID_METABOLISM_mRNAs_HM.pdf"), width = 5, height = 4.9)
print(p_glycerolipid_met)
dev.off()
```

```
## pdf
##   2
```

```
sel_genes <- gsc_c2[["REACTOME_CHOLESTEROL_BIOSYNTHESIS"]]
sel_genes <- GetOrtholog(sel_genes, species_from = "Hs", species_to = "Mm")
```

```
## Loading objects:
##   HCOP_ORTHOLOGY_TABLE
```

```
sel_genes <- unique(sel_genes)
sel_genes <- sel_genes[!is.na(sel_genes)]

pdf(file = file.path(reportDir, "P15038_LungManuscript_REACTOME_CHOLESTEROL_BIOSYNTHESIS_mRNAs_HM.pdf"), width = 5, height = 4.7)
```

```r
getIDMAPheatmapGG(idmap, intersect(sel_genes, sig_genes), cluster.row = TRUE, cex.labx = 2,
                  cex.laby = 1.5, title = NULL, col.lab = "black", object.only = TRUE)
dev.off()
```

```
## pdf
##   2
```

```r
sel_genes <- gsc_c2[["KEGG_FATTY_ACID_METABOLISM"]]
sel_genes <- GetOrtholog(sel_genes, species_from = "Hs", species_to = "Mm")
```

```
## Loading objects:
##   HCOP_ORTHOLOGY_TABLE
```

```r
sel_genes <- unique(sel_genes)
sel_genes <- sel_genes[!is.na(sel_genes)]

p <- getIDMAPheatmapGG(idmap,
                       intersect(sel_genes, sig_genes),
                       cluster.row = TRUE,
                       cex.labx = 2,
                       cex.laby = 1.5,
                       title = NULL,
                       col.lab = "black")
```

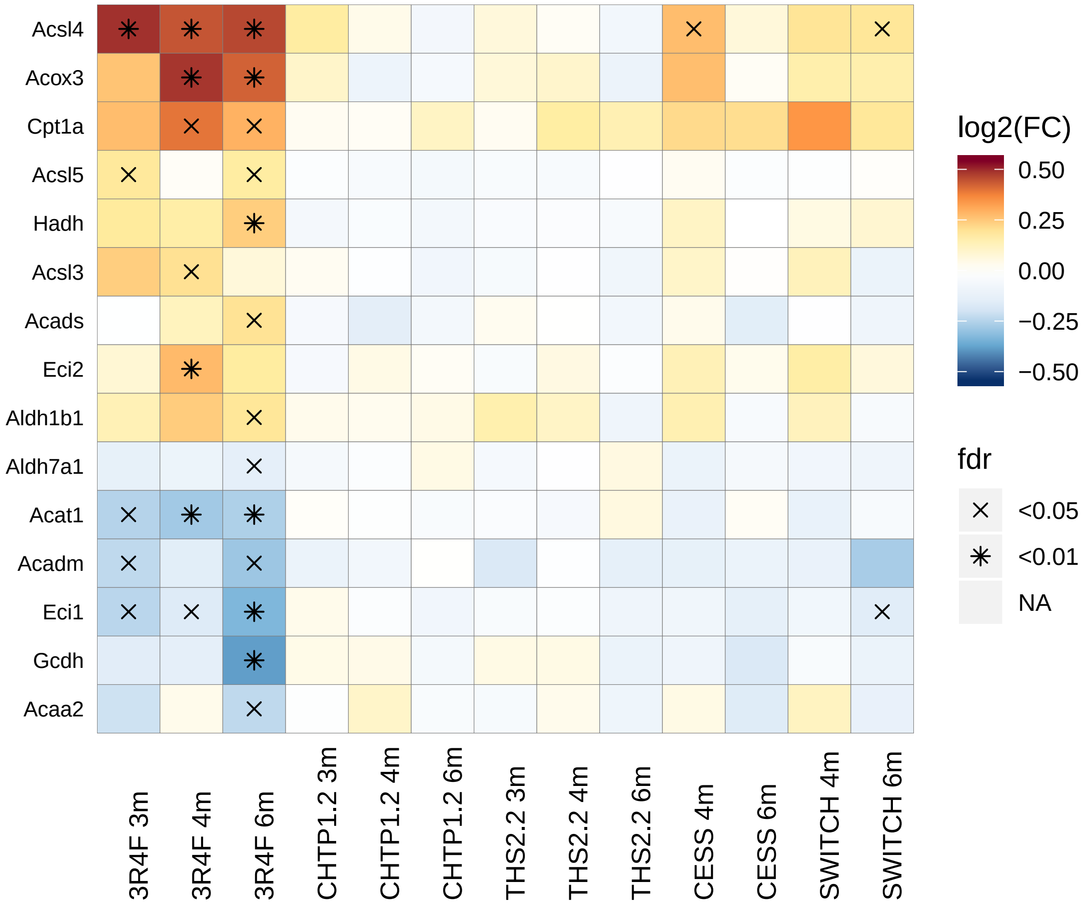


```r
p_fa_met_mRNAs <- p

pdf(file = file.path(reportDir, "P15038_LungManuscript_KEGG_FATTY_ACID_METABOLISM_mRNAs_HM.pdf"), width = 5, height = 4.7)
print(p_fa_met_mRNAs)
dev.off()
```

```
## pdf
##   2
```

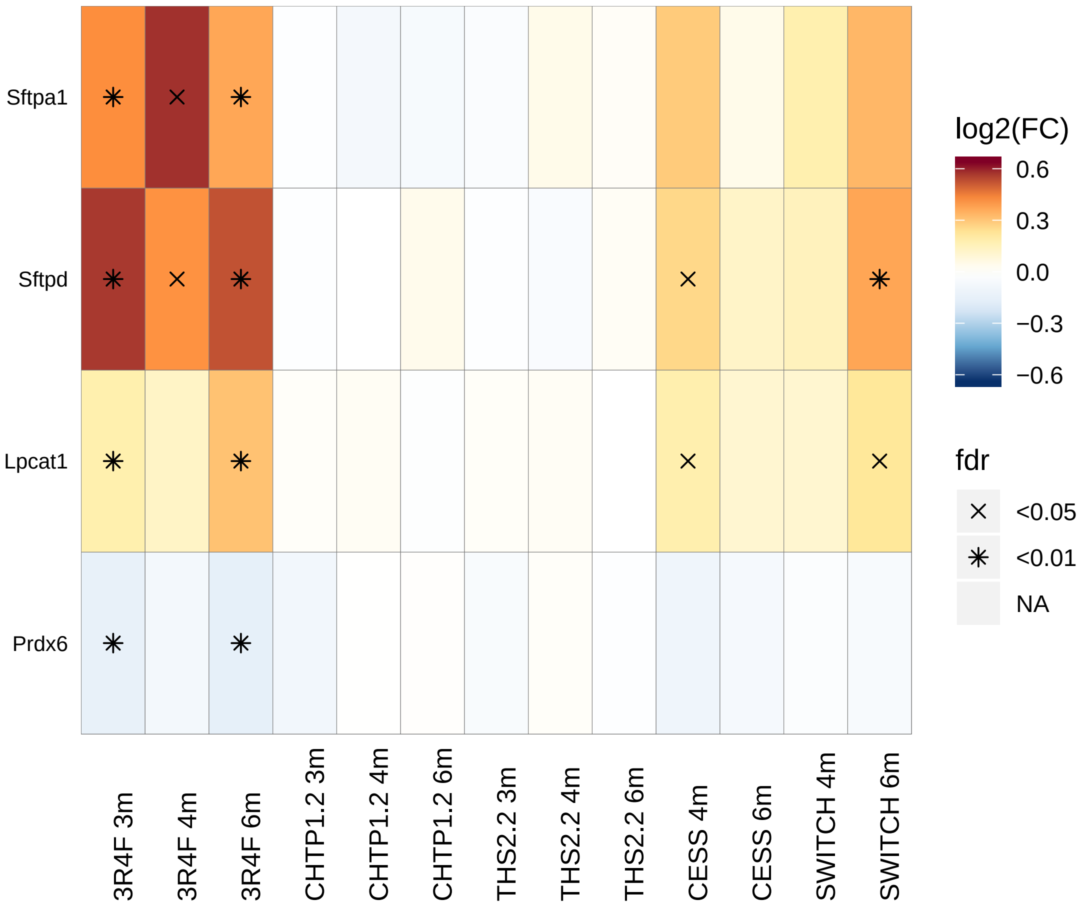

Figure 33: Abundance profiles of surfactant proteins.

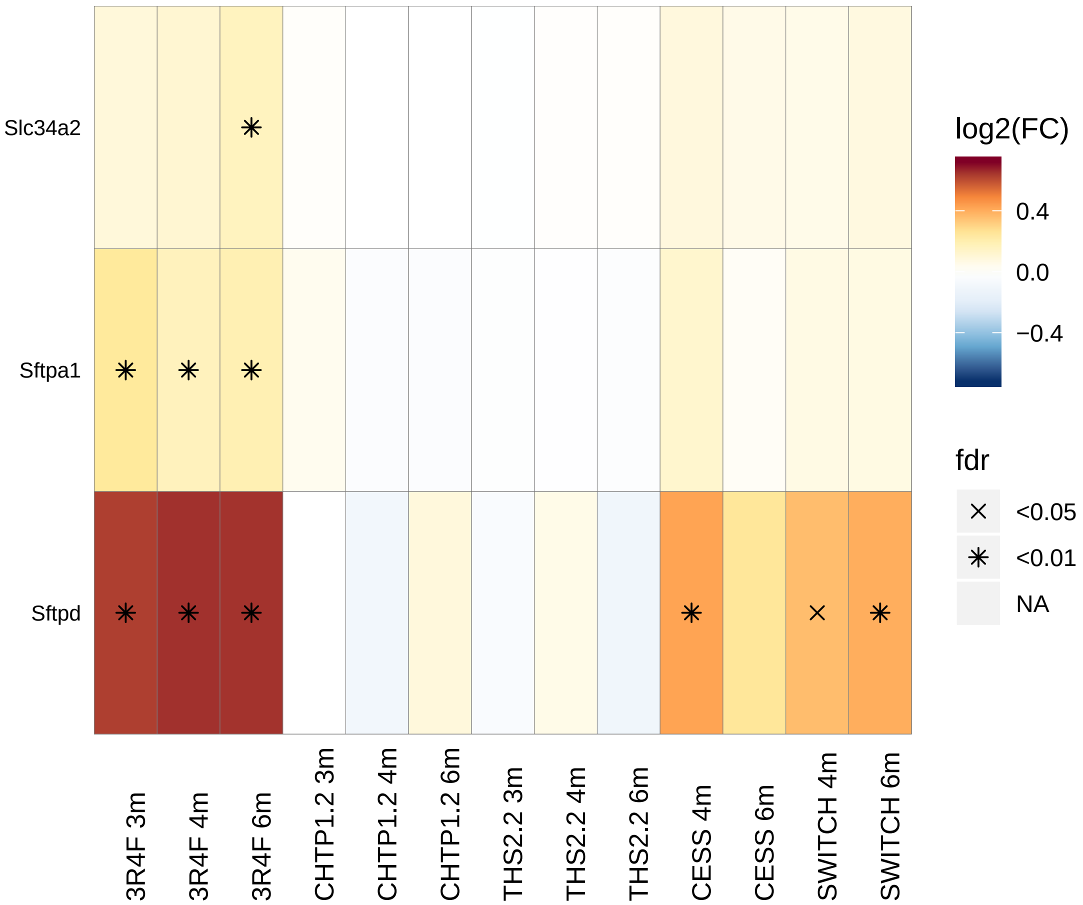

Figure 34: Expression profiles of surfactant mRNA transcripts.

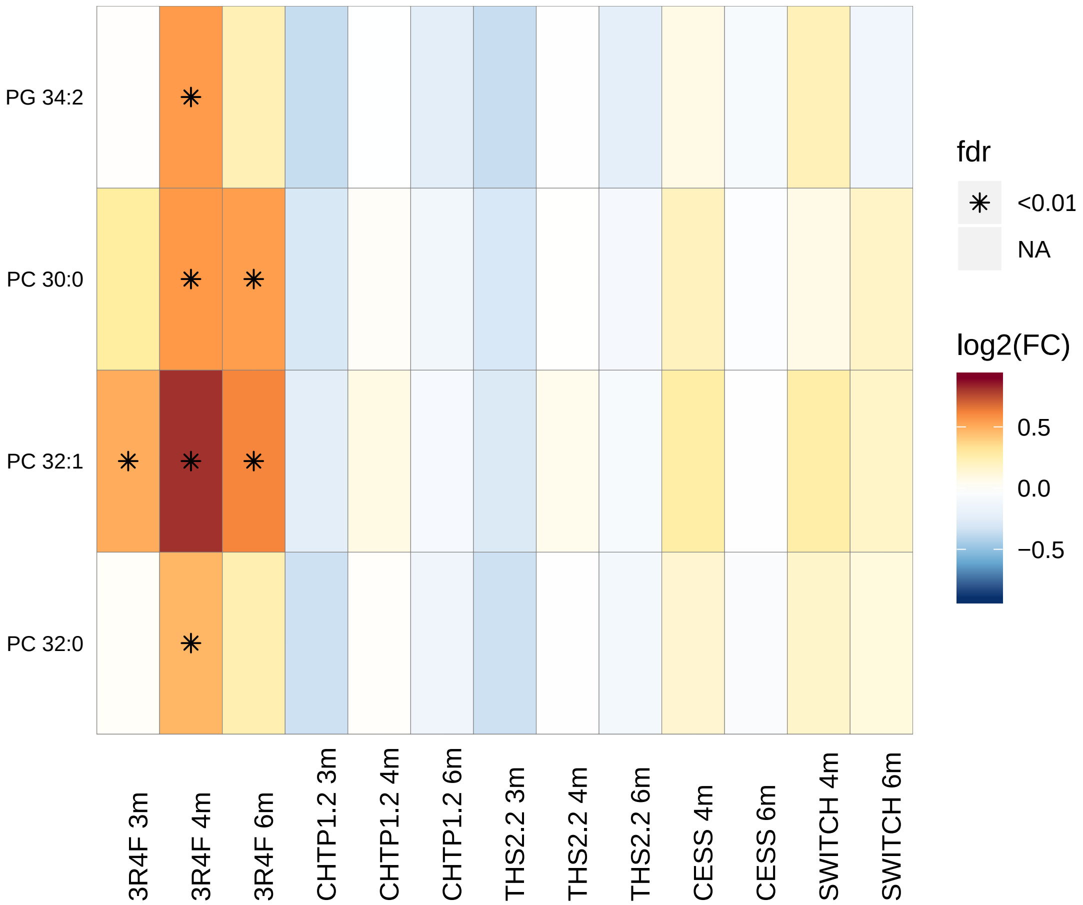

Figure 35: Abundance profiles of candidate surfactant lipids.

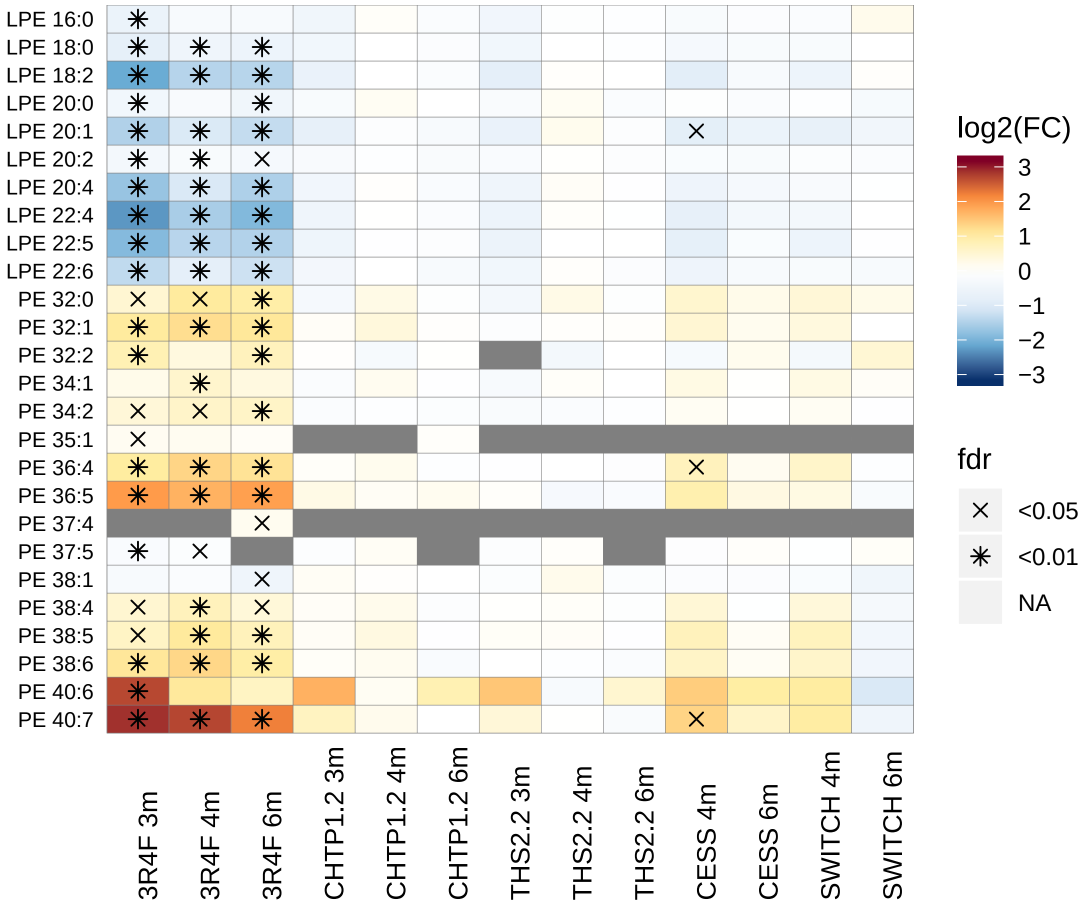

Figure 36: Oxidative stress-related mRNA transcripts.

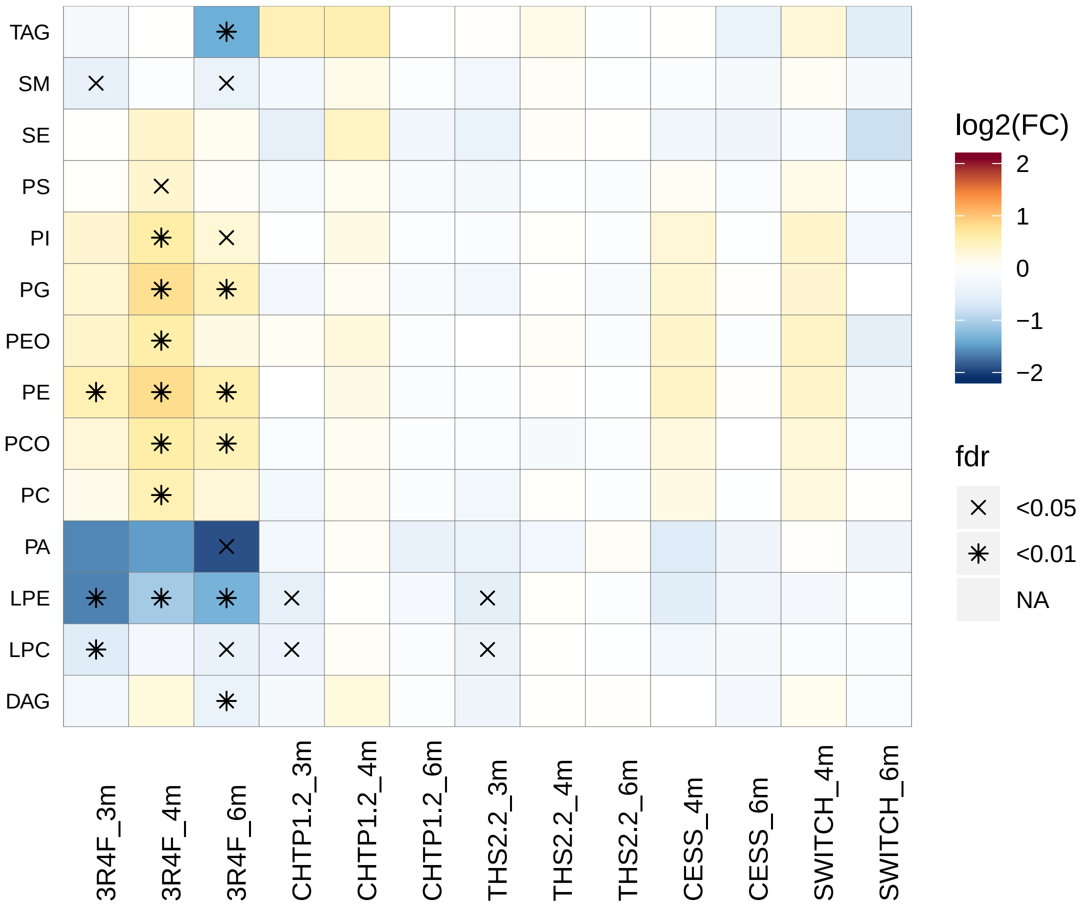

Figure 37: Lipid class heatmap.

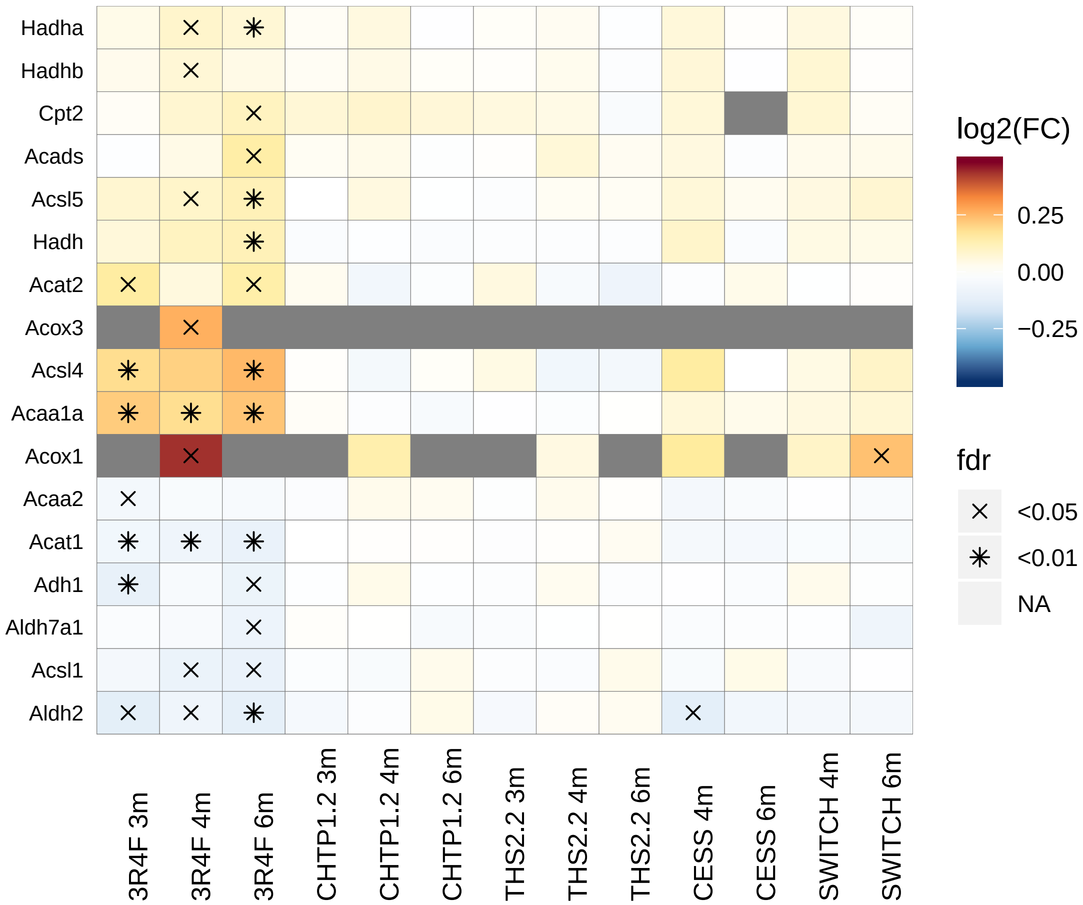

Figure 38: Expression of proteins involved in FA metabolism.

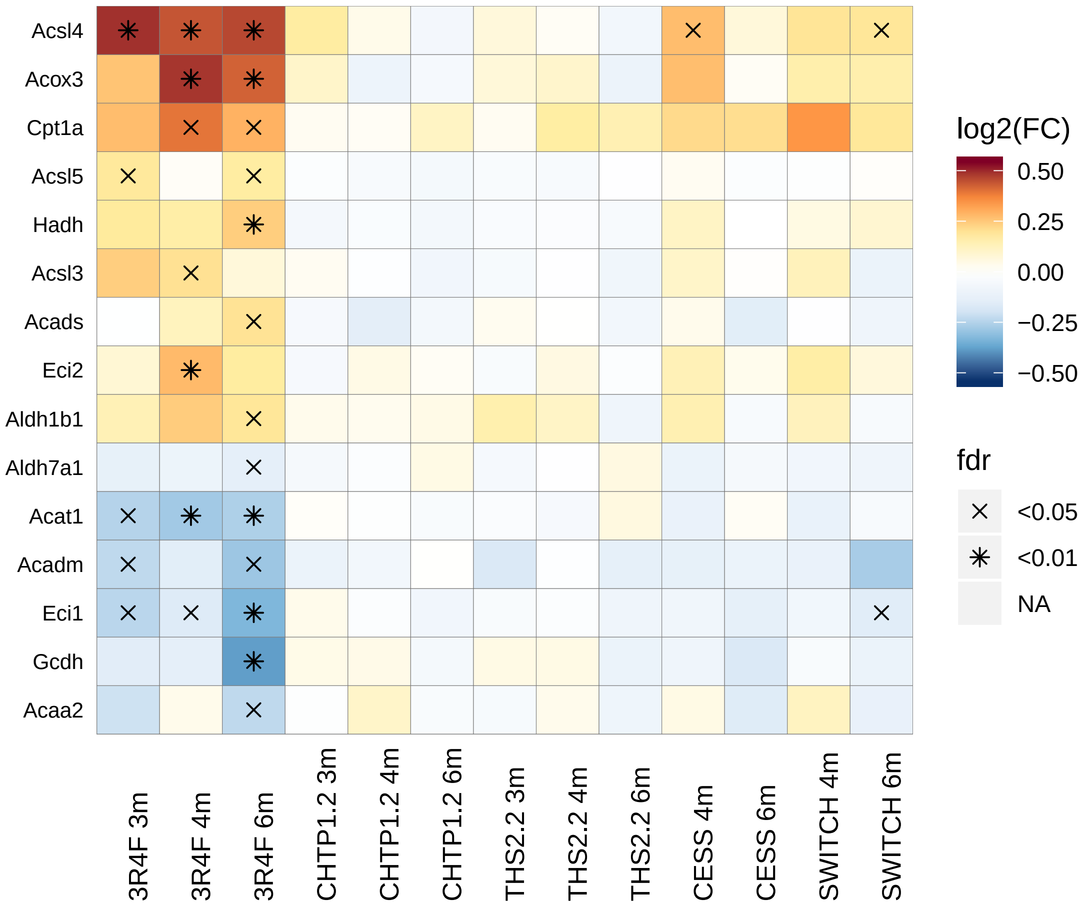

Figure 39: Expression of genes involved in FA metabolism.

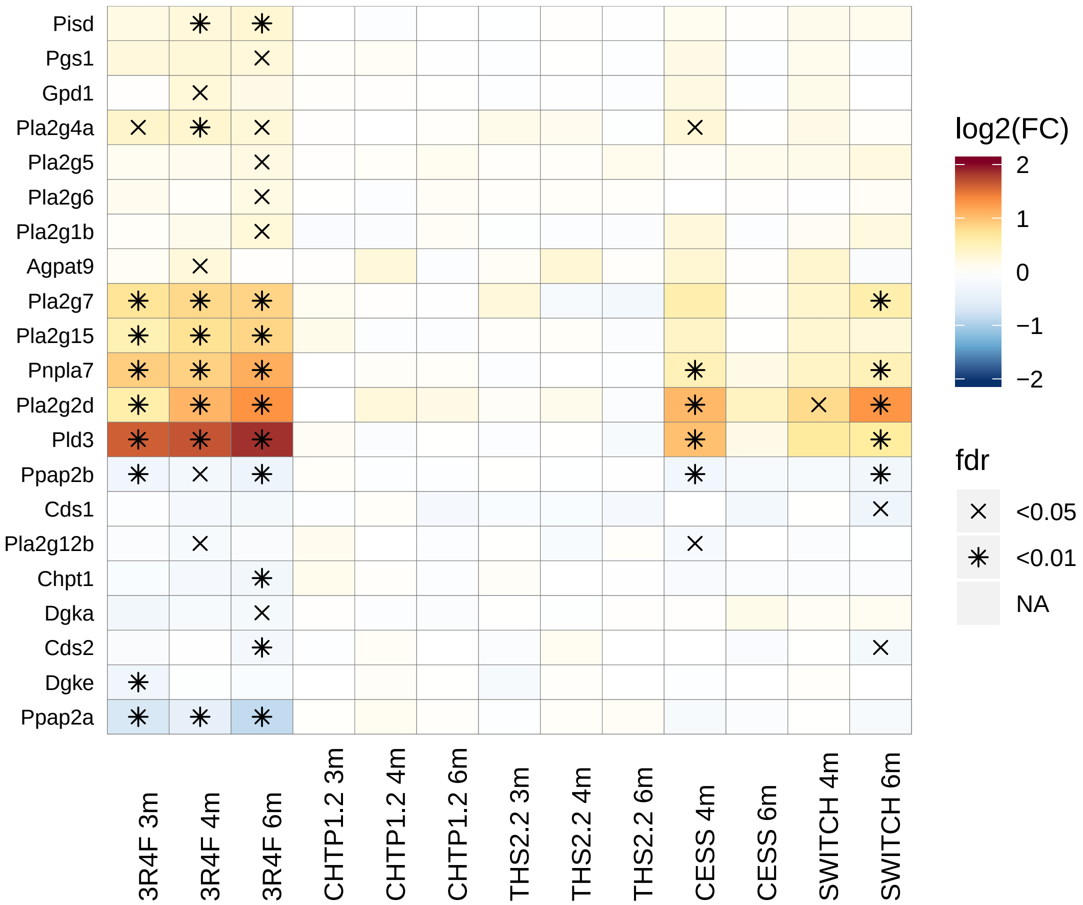

Figure 40: Expression of genes involved in glycerophospholipid metabolism.

18 Additional

```
idmap <- idmaps[["METABOLITE"]]
sig_mets <- unique(unlist(lapply(getDEG(idmap), function(x) as.character(x$nodeLabel))))
sig_mets <- sig_mets[!is.na(sig_mets)]

I_met <- dats[["METABOLITE"]]$I_metabolite
sel_mets <- I_met[I_met$SUPER_PATHWAY == "Nucleotide", "BIOCHEMICAL"]

pdf(file = file.path(reportDir, "P15038_LungManuscript_Nucleotides_HM.pdf"), width = 5, height = 5)
getIDMAPheatmapGG(idmap, intersect(sel_mets, sig_mets), cluster.row = FALSE, cex.labx = 2,
                  cex.laby = 1.5, title = NULL, col.lab = "black", object.only = TRUE)
dev.off()
```

```
## pdf
##   2
```

```
    # got question, whether C1qtnf4 detected by proteomics
    all_detected <- unique(unlist(lapply(idmaps[["PROTEIN"]], function(x) {
        as.character(x$nodeLabel)
    })))
    "Grb2" %in% all_detected
```

```
## [1] TRUE
```

```
    "C1qtnf4" %in% all_detected
```

```
## [1] FALSE
```

19 Competing Interests statement

The work reported in the associated publication involved candidate/potential modified risk tobacco products developed by PMI and was solely funded by PMI. Except for B.K., all authors are (or were) employees of PMI R&D or worked for PMI R&D under contractual agreements. B.K. is an employee of Metabolon Inc.

20 Version information

```
## R version 3.5.1 (2018-07-02)
## Platform: x86_64-pc-linux-gnu (64-bit)
## Running under: Red Hat Enterprise Linux Server 7.2 (Maipo)
##
## Matrix products: default
## BLAS: /gpfshpc/software/rhel7/R/R-3.5.1/lib64/R/lib/libRblas.so
## LAPACK: /gpfshpc/software/rhel7/R/R-3.5.1/lib64/R/lib/libRlapack.so
##
## locale:
##  [1] LC_CTYPE=en_US.UTF-8          LC_NUMERIC=C
##  [3] LC_TIME=en_US.UTF-8           LC_COLLATE=en_US.UTF-8
##  [5] LC_MONETARY=en_US.UTF-8       LC_MESSAGES=en_US.UTF-8
##  [7] LC_PAPER=en_US.UTF-8          LC_NAME=en_US.UTF-8
##  [9] LC_ADDRESS=en_US.UTF-8        LC_TELEPHONE=en_US.UTF-8
## [11] LC_MEASUREMENT=en_US.UTF-8    LC_IDENTIFICATION=en_US.UTF-8
##
## attached base packages:
## [1] tools     stats     graphics  grDevices utils     datasets  methods
## [8] base
##
## other attached packages:
##  [1] stringr_1.3.1      bindrcpp_0.2.2     PCSF_0.99.0
##  [4] igraph_1.2.2       visNetwork_2.0.5   stringi_1.2.4
##  [7] ggbeeswarm_0.6.0   mixOmics_6.6.1     lattice_0.20-38
## [10] MASS_7.3-51.1      MOFA_0.99.8        NPA_0.99.0
## [13] R6_2.3.0           limma_3.38.3       dplyr_0.7.8
## [16] plyr_1.8.4         gdata_2.18.0       plotrix_3.7-4
## [19] openxlsx_4.1.0     readxl_1.1.0       xlsx_0.6.1
## [22] reshape2_1.4.3     ggpubr_0.2         magrittr_1.5
## [25] egg_0.4.2          ggplot2_3.1.0      RColorBrewer_1.1-2
## [28] gridExtra_2.3      knitr_1.21
##
## loaded via a namespace (and not attached):
##   [1] reticulate_1.10            tidyselect_0.2.5
```

```
##   [3] robust_0.4-18              RSQLite_2.1.1
##   [5] AnnotationDbi_1.44.0       htmlwidgets_1.3
##   [7] grid_3.5.1                 BiocParallel_1.16.2
##   [9] devtools_2.0.1             munsell_0.5.0
##  [11] codetools_0.2-15           preprocessCore_1.44.0
##  [13] withr_2.1.2                colorspace_1.3-2
##  [15] Biobase_2.42.0             highr_0.7
##  [17] supraHex_1.20.0            rstudioapi_0.8
##  [19] stats4_3.5.1               robustbase_0.93-3
##  [21] RGraph2js_1.10.0           rJava_0.9-10
##  [23] labeling_0.3               slam_0.1-43
##  [25] GenomeInfoDbData_1.2.0     topGO_2.34.0
##  [27] bit64_0.9-7                pheatmap_1.0.10
##  [29] rhdf5_2.26.2               rprojroot_1.3-2
##  [31] xfun_0.4                   fastcluster_1.1.25
##  [33] sets_1.0-18                doParallel_1.0.14
##  [35] GenomeInfoDb_1.18.1        reshape_0.8.8
##  [37] bitops_1.0-6               fgsea_1.8.0
##  [39] DelayedArray_0.8.0         assertthat_0.2.0
##  [41] scales_1.0.0               nnet_7.3-12
##  [43] beeswarm_0.2.3             gtable_0.2.0
##  [45] processx_3.2.1             WGCNA_1.66
##  [47] rlang_0.3.0.1              splines_3.5.1
##  [49] lazyeval_0.2.1             acepack_1.4.1
##  [51] impute_1.56.0              hexbin_1.27.2
##  [53] checkmate_1.8.5            yaml_2.2.0
##  [55] backports_1.1.3            rsconnect_0.8.11
##  [57] Hmisc_4.1-1                relations_0.6-8
##  [59] usethis_1.4.0              gplots_3.0.1
##  [61] BiocGenerics_0.28.0        dynamicTreeCut_1.63-1
##  [63] sessioninfo_1.1.1          MultiAssayExperiment_1.8.1
##  [65] Rcpp_1.0.0                 progress_1.2.0
##  [67] base64enc_0.1-3            zlibbioc_1.28.0
##  [69] purrr_0.2.5                RCurl_1.95-4.11
##  [71] ps_1.2.1                   prettyunits_1.0.2
##  [73] rpart_4.1-13               cowplot_0.9.3
##  [75] S4Vectors_0.20.1           SummarizedExperiment_1.12.0
##  [77] ggrepel_0.8.0              cluster_2.0.7-1
##  [79] fs_1.2.6                   tinytex_0.9
##  [81] RSpectra_0.13-1            data.table_1.11.8
##  [83] dnet_1.1.4                 SparseM_1.77
##  [85] mvtnorm_1.0-8              whisker_0.3-2
##  [87] matrixStats_0.54.0         pkgload_1.0.2
##  [89] hms_0.4.2                  xlsxjars_0.6.1
##  [91] evaluate_0.12              IRanges_2.16.0
##  [93] testthat_2.0.1             compiler_3.5.1
##  [95] ellipse_0.4.1              tibble_1.4.2
##  [97] KernSmooth_2.23-15         crayon_1.3.4
##  [99] htmltools_0.3.6            corpcor_1.6.9
## [101] pcaPP_1.9-73               Formula_1.2-3
## [103] tidyr_0.8.2                rrcov_1.4-7
## [105] DBI_1.0.0                  corrplot_0.84
## [107] Matrix_1.2-15              readr_1.3.0
## [109] cli_1.0.1                  marray_1.60.0
## [111] parallel_3.5.1             bindr_0.1.1
## [113] GenomicRanges_1.34.0       pkgconfig_2.0.2
## [115] fit.models_0.5-14          foreign_0.8-71
## [117] piano_1.22.0               foreach_1.4.4
## [119] rARPACK_0.11-0             vipor_0.4.5
## [121] BH_1.66.0-1                XVector_0.22.0
## [123] callr_3.1.0                digest_0.6.18
## [125] graph_1.60.0               rmarkdown_1.11
## [127] cellranger_1.1.0           fastmatch_1.1-0
## [129] htmlTable_1.12             curl_3.2
## [131] gtools_3.8.1               rjson_0.2.20
## [133] nlme_3.1-137               jsonlite_1.6
## [135] Rhdf5lib_1.4.2             desc_1.2.0
## [137] pillar_1.3.1               GGally_1.4.0
## [139] httr_1.4.0                 DEoptimR_1.0-8
## [141] pkgbuild_1.0.2             survival_2.43-3
## [143] GO.db_3.7.0                glue_1.3.0
## [145] remotes_2.0.2              zip_1.0.0
## [147] iterators_1.0.10           bit_1.1-14
## [149] Rgraphviz_2.26.0           NPAModels_0.99.3
## [151] blob_1.1.1                 org.Hs.eg.db_3.7.0
## [153] latticeExtra_0.6-28        caTools_1.17.1.1
## [155] memoise_1.1.0              ape_5.2
```
